# The development of Euclidean axiomatics: The systems of principles and the foundations of mathematics in editions of the ***Elements*** in the Early Modern Age

**DOI:** 10.1007/s00407-015-0173-9

**Published:** 2016-02-24

**Authors:** Vincenzo De Risi

**Affiliations:** MPIWG, Boltzmannstraße 22, 14195 Berlin, Germany

## Abstract

The paper lists several editions of Euclid’s *Elements* in the Early Modern Age, giving for each of them the axioms and postulates employed to ground elementary mathematics.

In the following pages I give a list of the systems of principles adopted in the main editions of Euclid’s *Elements* in the Early Modern Age, as well as some information about the axioms in Antiquity and in the Middle Ages. The aim of such a list is to provide the reader with a quick survey of the development of mathematical axiomatics, stressing the most important turning points in the foundations of geometry.

As is well known, the *Elements* of Euclid represented for centuries the very model of scientific and deductive reasoning, and their diffusion and influence in Europe were only matched by the Bible and by a few other writings of the Fathers of the Church. They were translated, edited and commented upon hundreds of times, and these editions and commentaries shaped the scientific tools, the methodological standards and the mathematical language of many centuries. Euclid’s theorems were used to build further and more daring mathematical theories, or applied in the physical sciences, while the deductive structure of the proofs was studied by mathematicians, logicians and epistemologists as the ideal of reason itself. In this connection, a special historical role was played by the *principles* employed as grounds and foundations of the entire construction. Euclid had begun the *Elements* with a few unproven assumptions which had to be granted in order to establish his grand mathematical system. Since Antiquity, however, Euclid’s system of principles has been repeatedly discussed and challenged: A few gaps in the proofs were found and the missing arguments provided with additional axioms; other Euclidean principles were in turn proven from more elementary statements and thus removed from the list of axioms; still others were worded in simpler ways, or changed to accommodate philosophical qualms or to satisfy architectonic considerations; and a few more were added in order to extend the geometrical results beyond the boundaries fixed by Euclid. These changes in the system of principles grounding elementary mathematics were among the most important outcomes of the foundational studies carried out during the Early Modern Age. From the Middle Ages up to the beginning of the nineteenth century, hundreds of different editions of the *Elements* provided their own principles for the same corpus of mathematical theorems; while the statements of the Euclidean propositions remained more or less the same, and the proofs themselves were seldom changed, we witness, in this period, an remarkable explosion of new principles aimed at rigorizing, systematizing and improving Euclid’s work. The enormous creativity of these foundational attempts is attested by the invention of some 350 different axioms for elementary mathematics, with some works employing as many as fifty of them to ground the theorems of the *Elements* (Euclid himself probably only had ten principles). Many of these new axioms have great mathematical significance and introduced into the foundations of mathematics several important ideas that were to be given a full development only in the nineteenth and twentieth centuries. They concern continuity issues in the Euclidean plane, properties of incidence in two or three dimensions, Archimedean and non-Archimedean quantities, mereological relations, congruency and rigid motion, the Parallel Postulate and the Euclidean metric, or the first attempts at an axiomatization of arithmetic and algebra. In this connection, it is very important to stress that the best-known works on the foundations of mathematics produced in the modern age, such as Dedekind’s, Frege’s and Peano’s works on the principles of numbers, or Pasch’s, Pieri’s and Hilbert’s famous axiomatizations of geometry, by no means represent the sudden discovery of the need for rigorous foundations, but are rather the late and highly perfected outcomes of a long tradition that had been developing almost continuously from the time of Euclid onward. Even if it may be difficult to precisely ascertain a direct influence exerted by these early modern programs on, say, Hilbert’s *Grundlagen der Geometrie*, there can be no doubt that these important mathematical works of the previous centuries did indirectly affect modern, formal research on axiomatics. I will not pursue here, however, the search for the sources of nineteenth-century foundational studies, as this would require an altogether different approach. In any case, while the development of the foundations of mathematics in the last two centuries has already been studied in some depth, the history of axiomatics in the Renaissance and the Early Modern Age is still largely neglected, and I hope that the present essay can represent a beginning on, and an invitation to, this rich field of research, as well as a useful tool for further investigations.

I take this opportunity to express my heartfelt gratitude to Fabio Acerbi and Bernard Vitrac for their accurate reading of a draft of this paper that largely contributed to improve the present version. I greatly benefited from further suggestions offered by Niccolò Guicciardini, Jens Høyrup, Alexander Jones, John Mumma and Victor Pambuccian. I thank Sonja Brentjes for her invaluable help with the Arabic sources and for having helped me with the pseudo- Tūsī text, and Philip Beeley, who informed me about Wallis’s unpublished notes on Euclid and provided me with a copy of them. My special gratitude goes to the Library of the Max Planck Institute for the History of Science, for having provided me with several difficult-to-find editions of the *Elements*, and to Massimo Mugnai for his gift of a copy of Grandi’s version of Euclid. Any further suggestion will be very welcome and will improve future editions of this essay.

## Introduction

### The Euclidean tradition

In the present essay I consider the axiomatic systems of about a hundred editions of the *Elements*, ranging from Byzantine manuscripts down to early nineteenth-century printed volumes. In drawing up this list, I did not strive for completeness but rather for perspicuity, and I have selected a few important works among the enormous mass of manuscripts and books dedicated to the recension, translation and interpretation of Euclid. More exhaustive inventories of the editions of the *Elements* may be found in the bibliographical catalogues drawn up by Riccardi (1974) and Steck (1981); these catalogues are, however, themselves quite incomplete (in fact, a few of the books that I consider here are not even quoted in these accounts). I have mainly taken into consideration the early modern editions, although I have mentioned a few medieval manuscripts which are especially important for the history and the reception of the *Elements*. I also concentrated on Western mathematics, even though a few important developments in the Euclidean tradition first appeared in the medieval Islamic world; these works are only mentioned for the echoes that they have produced in the Latin tradition. Although a complete recension of the Arabic editions of Euclid is still missing, it should be noted, in any case, that it does not seem that they offered a great diversity of axiomatic systems (see for instance Brentjes 1997). Among the early modern editions, I have normally only listed the printed and published works. In this respect, however, I have made a few exceptions, including in my list Maurolico’s, Torricelli’s, Wallis’s, Pascal’s, Roberval’s and Newton’s handwritten work on the principles of geometry, which seemed to me too important to be ignored. Several other similar essays (such as Leibniz’s unpublished papers on the *analysis situs*) were not suitable to be easily included in the list and in any case did not exert any historical influence on the respective authors’ own contemporaries.

As a general rule, I have tried to include all those editions which proposed new axioms and principles, as well as those editions produced by mathematicians important in their own right (even if these editions did not represent any advancement in the axiomatic field), and the editions that possessed some special historical significance (by reason of their broad diffusion, for instance, or because they were the first editions in a particular modern language). I have only quoted the first edition of each work, unless the editions that followed introduced important changes in the system of principles (as is the case, for instance, of the second edition of Clavius’s commentary, or of Arnauld’s *Nouveaux Élémens*).

A serious limitation of the field examined here is the fact that I have only considered editions of the *Elements* themselves, leaving aside all other material on the foundations of geometry. It should, however, be borne in mind that several important changes in the axiomatic systems were proposed in monographic essays dealing with special problems of the Euclidean text, as is the case, for instance, with several Arabic treatises, Wallis’s or Saccheri’s studies on the Parallel Postulate, or Galileo’s new theory of proportions. A few more principles of mathematics were discussed in general mathematical works (such as certain treatises on algebra, or an essay on fluxions) which did not comment at all on Euclid. That development of the axiomatics, then, which is to be found in the editions of the *Elements* only gives a part of the whole story: and yet, this is (by far) the largest part, as the main discussions on the foundations of geometry in the Early Modern Age did indeed take place in the Euclidean commentaries. Some of these commentaries, moreover, introduced innovations and alterations with respect to the Greek text of the *Elements* to such an extent as to become almost new treatises on the foundations of geometry. There is, therefore, a certain degrees of arbitrariness in the decision to include certain works in the list of the Euclidean commentaries, even though they departed very far from the original structure of the *Elements*, while excluding certain others as moving really too far away from the original to be still considered discussions of Euclid’s work itself. This is especially true as regards eighteenth-century manuals on elementary mathematics, which often offered a complete reworking of the content of the *Elements* and no longer had the form of a commentary at all. I have reproduced here the systems of axioms contained in a few of these manuals, choosing the most important representatives of the age (such as Simpson’s or Kästner’s books); the reader should, however, be aware that dozens of such volumes were produced in Europe at this time—and in eighteenth-century Germany in particular.

It may perhaps be useful to provide, at this point, a short synoptic sketch of the general development of the systems of axioms employed in the various editions of the *Elements* from Antiquity up to the modern era.

#### Antiquity and the Middle Ages

Euclid’s *Elements* (in thirteen Books) were probably written in the third century BC, drawing together materials from a more ancient mathematical tradition, and surely underwent several changes and modifications already during the Hellenistic Age. Several centuries later, the mathematician Theon of Alexandria (fourth century AD) prepared an edition of the *Elements *which has had a great importance in the following tradition of the text. The oldest extant copies of the *Elements* are, however, Byzantine manuscripts dating back to the beginning of the ninth century; later Greek manuscripts were in circulation among scholars during the Renaissance and in fact provided the Greek text for the first editions of Euclid in the modern world. One further important manuscript was discovered only at the beginning of the nineteenth century by François Peyrard, and it apparently contained a version of the *Elements* older than any other, and possibly predating Theon’s editing of Euclid. At least, it seems that its author had access to both Theonine and non-Theonine manuscripts (it should be remarked that this statement, endorsed by many scholars, has been challenged by some others; see Knorr 1996). It is still considered the best guess which we possess regarding the original form of Euclid’s work, and has been employed by the Danish philologist Johan Ludvig Heiberg to prepare the only critical edition of the *Elements* (1883–1916) that we currently have. For a history of the Greek text of the *Elements*, see for instance Rommevaux et al. (2001).

All these Greek manuscripts (both those pertaining to the “Theonine” tradition, and Peyrard’s text) show many definitions prefacing several books of the *Elements*, and a set of fourteen of fifteen other principles (depending on the manuscripts) at the beginning of Book One. The latter principles were considered to be the only statements on which the mathematical construction of Euclid was to be based, and are divided into a first list of *postulates*  and a second list of *common notions*  or, as they were later to come to be called, *axioms*. The rationale behind this division of the principles into ‘postulates’ on the one hand and ‘common notions’ on the other was a subject of debate and controversy already in ancient times, and several philosophers and mathematicians advanced their own opinions on the epistemological status of these two distinct kinds of assumptions. We will not discuss this difficult topic here, but will note that all the editions of the *Elements* in the Early Modern Age, with no exception, were to preserve this distinction (even if they interpreted it in different ways).

Some principles among the fourteen or fifteen contained in the oldest manuscripts, however, came to be considered spurious already in Late Antiquity, and Proclus (fifth century, the author of a crucially important commentary on the First Book of the *Elements*) informs us that a number of them were in fact later interpolations. Modern philology inclines to accept Proclus’s opinion and attempts to prove (even if on different grounds) that some of the principles must have been added in the ancient transmission of the text. A largely (but not unanimously) endorsed opinion among scholars is that Euclid originally had only ten principles (five postulates and five common notions), while the other axioms are Hellenistic additions. Some further doubts are usually raised about two common notions (CN4 and CN5) that some scholars would like to regard as interpolations as well; if this latter view holds true, then Euclid only had eight principles. We know, in any case, that several ancient mathematicians discussed the Euclidean system of principles, and began to add, change or remove a number of axioms and postulates; this seems to have been the case, for instance, of Apollonius, Geminus, Ptolemy and Pappus. In particular, Heron of Alexandria (probably first century AD) wrote a commentary on the *Elements* in which he addressed a few gaps in the Euclidean proofs and tried to fill them introducing new assumptions (which he may, however, have regarded as provable). While Heron’s commentary is now lost, it was still known in the Islamic Middle Ages (see for instance Brentjes 1998, or the introductory essay to Acerbi and Vitrac 2014). A few fragments of it, moreover, circulated in the commentary to the *Elements* written by Proclus, whose text enjoyed only a very limited circulation in the Middle Ages, but was rediscovered in the Renaissance. Similarly, Simplicius’s commentary to the *Elements* (sixth century) was extensively read in the Arabic-speaking world, but was later lost. A few of the axioms added in the Middle Ages and the Renaissance, in fact, derive from these Greek commentaries, and from Heron in particular, as the philosophers Proclus and Simplicius did not dare to add their own principles to the “most perfect” work of Euclid.

During the early Middle Ages knowledge of Euclid disappeared from the West (except for a few fragments preserved in a very elementary mathematical work attributed to Boethius), but flourished in the Islamic world. The *Elements* were translated into Arabic twice in the early ninth century by Al-Hajjāj, and again a few years later by Isḥāq ibn Ḥunain (a better version than Al-Hajjāj’s, revised by the great mathematician Thābit ibn Qurra). These editions were translated from Greek manuscripts that were not very dissimilar to those which we still possess, and thus presented the extended system of principles (fourteen or fifteen of them) which were already common in Greek Late Antiquity. In the tenth century, the mathematician An-Nayrīzī wrote an important commentary on Euclid, drawing on Simplicius’s and Heron’s (and those by others) new additional assumptions. Even though this was not an edition of the *Elements* themselves, but rather a commentary thereon, it was clear that Euclid’s system of principles could, and probably should, be enlarged to fill the gaps present in several proofs. An-Nayrīzī’s commentary on Euclid survived as regards Books I–VI and X, with a very short fragment on Books VII and VIII and a longer fragment on Book IX.

The *Elements* of Euclid reappeared in the West in the first half of the twelfth century, when Adelard of Bath translated into Latin an Arabic manuscript containing (a modified version of) Al-Hajjāj’s translation. Adelard’s edition was followed by several other translations made from similar Arabic sources (and thus with similar principles), and by the different translation of the Isḥāq–Thābit’s version made by Gerardo da Cremona. Gerardo, however, also translated An-Nayrīzī’s commentary, and this paved the way for a reworking of the Euclidean axiomatics. Already in the edition of John of Tynemouth a few new principles were added at the beginning of Book VII of the *Elements*, which were partly drawn from An-Nayrīzī. In the thirteenth century, Campano da Novara edited a final version of the Euclid of medieval tradition, collating together several previous translations and even an independent work (the *Arithmetica* by Jordanus de Nemore) to fashion a highly successful edition of the *Elements*. It contained sixteen geometrical principles in Book I and fourteen arithmetical principles in Book VII. Campano’s version was widely used in the following centuries, and it was the first edition of the *Elements* to be printed, in 1482.

#### The Renaissance

Campano’s edition and its axiomatic system were used several times in the following century. Luca Pacioli produced an improved version of it, and Niccolò Tartaglia translated it into Italian in 1543 (altering a few axioms thereby). Tartaglia’s edition inaugurated the translations of Euclid into modern languages and was followed by Xylander’s translation into German (1562), Forcadel’s into French (1564), Billingsley’s into English (1570), Zamorano’s into Spanish (1576) and Dou’s into Dutch (1606). In the meantime, Greek manuscripts had begun to arrive in Europe, and Bartolomeo Zamberti had translated the *Elements* into Latin directly from the Greek (1505), thus opening a new era in the philological reception of the text. In 1533 Simon Grynaeus published the *editio princeps* of the Greek text. The latter editions produced on the basis of the Greek text once again proposed the ancient system of principles which featured no arithmetical axioms nor any of the further corrections made by Campano. Grynaeus’s edition, however, was based on a Greek manuscript that (probably following Proclus’s hint) changed the division between postulates and axioms, and only admitted three postulates (P1, P2, P3, i.e., the clearly constructive ones), listing the others (P4, P5, P6) among the axioms. Grynaeus, in fact, even appended an edition of Proclus’s commentary to Euclid’s text: so that the system of principles contained in the *editio princeps* of the *Elements* was in fact a Neoplatonic philosophical construction. In any case, Grynaeus’s edition of the Greek text remained the only one available for a very long time, and most of the “philological” editions of Euclid in Latin or in modern languages which were prepared in the following two centuries simply followed Grynaeus’s altered system of axioms and postulates (e.g., Caiani, Camerarius, Scheybl, Gracilis, Xylander, Dasypodius, Forcadel, Candale, Dou, Henrion, Keill).

A debate on the superiority of Zamberti’s and Grynaeus’s Greek sources over Campano’s Arabic ones then ensued; comparative editions were prepared; and even if, in the end, the Greek tradition prevailed in the hearts of the humanists and Campano’s version was abandoned by the end of the sixteenth century, this latter’s richer system of principles continued, nonetheless, to charm several geometers who prized mathematical perspicuity (and soundness) above philological exactitude.

A clearly recognizable line of French mathematicians dared to modify Euclid’s system of principles to comply with mathematical correctness. Jacques Lefèvre d’Étaples published a new, enlarged system of arithmetical principles (drawing on Campano); Oronce Fine began to add a few new geometrical axioms; and Jacques Pelletier showed no hesitation in criticizing Euclid for his use of rigid motion in geometrical proofs, thus changing the system of principles in such a way to be able to dispose of these kind of demonstrations. A few years later, the mathematician Pedro de Monzón, who had translated Fine into Spanish, collected the fruits of this innovative tradition and published an edition of Euclid that comprised forty-three principles (both geometrical and arithmetical). Among the studies on the foundations of geometry in the sixteenth century, we should also mention the edition of Euclid produced by Herlinus and Dayspodius in 1566, which rewrote all the proofs of the *Elements*, molding them into syllogistic form; this important advancement in the logical understanding of geometrical reasoning and the deductive structure of the *Elements*, however, simply accepted Grynaeus’s system of principles, and added nothing to the science of axiomatics in the strictest sense. The culmination of the sixteenth-century debates on the *Elements* was attained in the 1570s, when Commandino and Clavius printed their important editions of the *Elements*.

Federico Commandino published in 1572 his Latin (and Italian) version of Euclid, which was based on different Greek manuscripts and was in many respects more correct than Grynaeus’s *editio princeps* (even though Commandino produced no edition of the Greek text itself). Even if Commandino clearly had important philological aims, he was also an outstanding mathematician and endorsed several changes in the axiomatic system which had been found necessary by the preceding tradition, adding a few further modifications himself; he added for the first time certain axioms in Book V and in Book X of the *Elements*. The very remarkable outcome is an edition of Euclid which can certainly be trusted for its philological accuracy but which nonetheless appeared much richer in foundational discussion (with a total of thirty-nine axioms and postulates), and many subsequent editions, both in Latin and in other, modern languages, in the course of the seventeenth and eighteenth Centuries, were simply to be based on Commandino’s text.

In 1574 the Jesuit mathematician Christoph Clavius published another crucially important edition of Euclid in Latin. Clavius’s edition was by far the longest and most complete edition of the *Elements* ever produced, and was accompanied by a very long commentary that collected almost everything that had been published about the Euclidean text in the previous centuries. For this reason it quickly became the reference work for any further discussion on the foundations of geometry in the following two hundred years, and easily the most important edition of Euclid that was ever published. Clavius believed that the original Greek text was important and had to be preserved but that mathematical perspicuity was a higher aim; his compromise went further than Commandino’s in correcting Euclid. Clavius himself envisaged a few new axioms that were useful, in his view, in order to rigorize the proofs of the *Elements*; given the enormous success of this edition (and the fact that it immediately became the model for all the further Jesuit editions of Euclid), these new Clavian axioms were successively endorsed by almost the whole community of mathematicians. Another important aspect of Clavius’s work on elementary geometry is that, in the 1580s, he came into contact with an Arabic edition of the *Elements* attributed to Nasīr ad-Dīn at-Tūsī (thirteenth century), and here he discovered the great difficulties that the Arab mathematicians had faced in attempting to prove the Parallel Postulate. In the 1589 edition of his commentary Clavius added Nasīr ad-Dīn’s alleged proof of the Parallel Postulate, together with a few reflections of his own, and this episode marked the beginning of the rich epoch of the attempts to prove the Parallel Postulate in Europe—an epoch that culminated in the eighteenth century, with the subsequent discovery of non-Euclidean geometry. Clavius’s complete system of principles, which was to remain a milestone in the following developments, contained four postulates and twenty axioms in Book I (elementary plane geometry), one axiom in Book V (the theory of proportions), three postulates and twelve axioms in Book VII (arithmetic), and one postulate and three axioms in Book X (irrational magnitudes).

#### The seventeenth century

The seventeenth century was the most creative age in the invention and development of new principles for geometry and arithmetic, and in many respects the highest point of research into the foundations of mathematics in the Early Modern Age. Following an age in which the main goal seemed to be that of the restitution of an accurate text of the ancient mathematical work after the corruptions of the Middle Ages, the new century decidedly aimed at the reform and improvement of the *Elements* beyond what Euclid himself had been able to do. To this effect, several completely new systems of axioms were devised in order to ground elementary mathematics. This innovative line of attack to the ancient text was especially popular in France and Italy.

French mathematicians often endorsed an epistemology of Ramist origins (that was later contaminated by similar stances emerging out of the new Cartesian tradition) which was intent on designating as an “axiom” any clear and evident mathematical proposition, and in any case any proposition the proof of which could be considered more complicated, and thus less evident, than the statement itself. Such an epistemology, which was stated in its most classical form in the *Logique de Port-Royal* (1662), led to a flourishing of “evident” principles in this century’s editions of Euclid which was without precedent in previous ages. Several mathematicians simply wrote down dozens of axioms and postulates at the beginning of the *Elements* without even troubling themselves about whether they were actually needed for the proofs or whether they could be deduced one from the other. Into these highly pleonastic systems of principles, which were not inspired by any consideration of economy of means, a few good mathematicians also, indeed, inserted several important axiomatic principles which had been overlooked in the past and which were to play a central role in future axiomatizations. We may mention in this connection Herigone’s edition of the *Elements* (1634), which contains forty-nine axioms and postulates, the greater part of them being extensions of the Euclidean common notions (i.e., principles on mereology and equality), and also the edition by Claude Richard (1645), with forty-six principles mostly dealing with geometry and introducing a few “topological” axioms to guarantee the continuity of the figures and the existence of their intersection points (the so-called Line–Circle or Circle–Circle continuity axioms). Richard was a Jesuit, and, like him, other French Jesuit masters continued Clavius’s work on the foundations of mathematics, with the additional aim of producing pedagogically efficient handbooks of geometry; among their most remarkable outcomes, we may mention Tacquet’s radical reform of the *Elements* and their principles, as well as the work by Dechales and Fabri. On the other side of the political–religious divide of the day, the Jansenist theologian Antoine Arnauld wrote an important new reworking of elementary mathematics (1667, 1683), which also included many new axioms (forty-two in total) and was followed by several editions loosely inspired by it (by Lamy, Malézieu, Sauveur, and others). The most important breakthrough was probably achieved, however, by Pascal and Roberval, who wrote two very important essays on the foundations of geometry, both of which clearly show their mathematical genius. Neither of them, however, was published. Roberval’s work survived in its entirety, while we only have the introduction (and the axiomatic basis) of Pascal’s essay on elementary mathematics. The latter text was copied by Leibniz in Paris in 1675, and largely inspired Leibniz’s own work on the foundations of geometry (the *analysis situs*) which also offers a thoroughgoing discussion on axiomatics, but remained unpublished (and thus historically ineffectual) among Leibniz’s papers in Hannover (on Leibniz’s *analysis situs*, see De Risi 2007, 2015b).

Italian studies on Euclidean axiomatics were mostly carried on by the school of Galileo and concentrated especially on the reform of the theory of proportions, since Galileo himself had attempted such a construction (in the unpublished *Fifth Day* of the *Discorsi*) and the topic was central to his project of the mathematization of mechanics. It is to Torricelli (1647), Borelli (1658), Viviani (1674), Giordano (1680) and Marchetti (1709) that we mainly owe the progress made in this field. As compared to the French tradition, these Italian axiomatizations show less creativity in producing new axioms, but a greater attention to their systematic function and the economy of principles. From a purely logical point of view, some of these books may be considered to be the culmination of the logical reflection of the *Elements*, and Borelli’s *Euclides restitutus*, in particular, marks a high point in researches on the foundations of mathematics.

#### The eighteenth century

In the eighteenth century, the foundational discussion on mathematics shifted for the most part outside the scope of the *Elements*. Most efforts were now dedicated to the many issues raised by the new Calculus, and reflection on the foundations of mathematics was often aimed rather at justifying the employment of infinite or infinitesimal quantities; these discussions, however, are seldom to be found in an edition of Euclid. Even elementary mathematics is from time to time grounded on different principles, and algebra (for instance) offered the possibility of a dissimilar foundational approach, grounded in symbolic manipulations rather than simple geometrical constructions. The *Elements* were no longer regarded as the proper place for advancing a scientific discussion, but rather just as the most classical handbook for a basic schooling in mathematics. The outcome of this modified approach was, on the one hand, a growing attention to pedagogical improvements in the teaching of elementary geometry, and thus a proliferation of simplified editions for students, and on the other hand a renovated search for a more reliable and philologically accurate edition of the *Elements*.

The former attitude was especially widespread in France and Germany, where a large number of Euclidean textbooks were produced, often largely departing from the original text of the *Elements*. These systematizations of elementary mathematics do not offer much, however, as regards the foundational domain, and their axiomatizations are often oversimplifications of older editions. Whereas the epistemology of the “clearness and evidence” of mathematical principles had resulted in France, in the previous century, in an overproduction of axioms, the same epistemology in the eighteenth century often yielded axiomless editions of the *Elements*, which merely made mention of the fact that several evident assumptions were implicitly employed in the development of the proofs (this is the case, for instance, of the celebrated book on elementary geometry written by Clairaut in 1741); a few other editions listed a small number of axioms, claiming that these were just examples of an infinite array of principles that cannot but remain implicit (see Deidier, 1739; or Legendre, 1794). In Germany the surviving Scholastic idea that all the axioms should ultimately be proven from the definitions also engendered several axiomless textbooks which enjoyed a wide diffusion (the most important representatives of these being Christian Wolff’s, Andreas Segner’s and Wenceslaus Karsten’s German and Latin treatments of elementary mathematics). Nevertheless, there can be noted a few interesting developments in the area of axiomatization in this period, such as Samuel König’s and Abraham Kästner’s axiomatizations from 1758.

The philological attitude toward the *Elements*, on the other hand, was especially common in Britain, where David Gregory produced an emended edition of the Greek text (1703) which may be considered the first improvement on this latter since Grynaeus. Robert Simson, likewise, published a highly successful edition of the *Elements* (1756) which aimed at restoring the Euclidean text to the state in which it existed before the interventions of Theon of Alexandria; this was in fact later to be accomplished by Peyrard through his discovery of an actual pre-Theonine manuscript (or, at least, a manuscript in which non-Theonine elements are to be found); Simson’s attempt was based merely on some lucky guesses. If these philological editions offer nothing in the way of an actual development of axiomatics, the commentaries by Thomas Simpson (1747) and, to a lesser extent, John Playfair (1795), nonetheless added several important principles, and attempted a new foundation of elementary mathematics. It may be mentioned that the axiomless attitude toward elementary geometry is well attested in Britain as well, with John Leslie’s celebrated *Elements of Geometry* from 1809, and a famous *Geometry without axioms*, published by Perronet Thompson as late as 1833.

We end our survey with Peyrard’s edition of Euclid, published in 1814. This book, in which the Greek text was established from several manuscripts, marked the most relevant advancement in textual analysis so far and opened the rich era of nineteenth-century Euclidean philology which eventually found its culmination in Heiberg’s 1883 edition of Euclid (largely based on the manuscript found by Peyrard). The discussion of the system of principles in the nineteenth century mostly developed in the form of monographic essays or independent treatises (such as those by Pasch, Frege, Peano or Hilbert) which completely abandoned the literary form of commentary on the text of Euclid. In the same years in which Peyrard was searching for Greek manuscripts, and in the following decades, when his recension of the text begun to acquire authority and to be translated into several languages, Poncelet and Staudt were developing new foundations for geometry starting from the projective properties of space, and Lobachevsky and Bolyai were publishing their treatises on hyperbolic geometry. The gradual development of projective geometry and the non-Euclidean systems, as well as the subsequent birth of abstract algebra and set theory, shifted most of the foundational attention to these new fields. It became clear that the *Elements* of Euclid could no longer stand as the systematic ground of modern mathematics, since the ancient text could not bear the weight of these new axiomatizations. A completely different approach to the foundations of geometry was then in need.

### Axioms and postulates

I cannot attempt to present here a whole history of the epistemology of mathematics; nor to discuss of the meaning of the notions of axiom and postulate from Antiquity down to modern times; nor to give a list of the many and diverse goals that mathematicians may have aimed at in reshaping the system of the Euclidean principles (for a few contemporary suggestions, see for instance Schlimm 2013). It should at least be noted, however, that many mathematicians in the Early Modern Age believed that axioms and postulates were in fact provable from definitions. They were not so much considered to be statements which *admitted* *of* no proof as statements which *required* none (since they were sufficiently evident already in themselves). In fact, however, a few of the most important editions of Euclid *did* explicitly prove the axioms and postulates; the commentary by Clavius, for instance, gave demonstrations for all mathematical principles except definitions. It then became a point of controversy whether or not it was useful to prove the axioms at all; many French authors during the seventeenth century, for instance, claimed that Clavius’s exercise had plainly been futile. A further disagreement concerned the criterion which was to be applied in distinguishing a principle from a theorem (as both of these latter are provable); the most common (and quite generic) answer given here was that, whereas an axiom or a postulate is *immediately* proven from the definitions, a theorem is only *mediately* proven from these latter (and from other theorems, and from other axioms); the notion of “immediacy” evoked here, however, remained quite vague. As the number of principles grew their demonstration was often omitted, and in many cases it is not easy to understand how such principles could have been proven or explained away. It has already been noted that many authors also claimed that the proper number of axioms should be regarded as infinite, and that the explicitly stated principles should be considered as merely examples and instances of the innumerable statements which immediately follow from the definitions themselves (or from the “essences” of the geometrical entities defined). I have found the first trace of this claim in a few medieval manuscripts of the *Elements* in the tradition of Robert of Chester (even though previous, more subtle traces of this attitude may be found in Ibn al-Haytham and the Arabic tradition), from whom the idea passed to Campano, and thus on into the mainstream epistemology of geometry. In any case, it should be clear that there is a sense in which *definitions alone* were considered the proper principles of mathematics, while axioms and postulates were often regarded as derivative statements.

All this notwithstanding, I have, in the following pages, only listed axioms and postulates, leaving aside the definitions. The omission, however, is not as serious as it may at first appear. It should be remarked that the system of definitions is much more stable than the system of axioms and postulates, and the definitions found in the early modern editions of the *Elements* are quite close to the list found in the Greek manuscripts. When the moderns add or modify a few definitions, moreover, this seldom has any effect on the further mathematical development of the treatise. Some definitions were only changed in order to dispel certain purely epistemological qualms (for instance, some authors define a circle as the trace left by the endpoint of a rotating segment rather than as a line equidistant to the center, with the aim of giving an explicit construction and thus a “*real definition*” of the geometrical entity in question); other definitions were added in order better to explain a few passages in Euclid, or so as to be able to add a few theorems. In most cases, the original Euclidean definition is simply placed side by side with the new definition, which is then regarded as equivalent to this latter, without any proof being given for this assumption. In short, then, the system of definitions was usually only modified in respect of foundational preoccupations which did not affect the further mathematical treatment. The axioms and postulates which are derived (or which should be derived) from these definitions are the statements which are properly employed in the proofs and which therefore affect the deductive structure of the *Elements*.

Nevertheless, it should be noted that a number of new definitions had real consequences for the axiomatics: This is especially true for the definition of a straight line (which troubled several mathematicians over a period of many centuries), for the definition of parallel lines (as several such definitions were conceived in order to prove the Parallel Postulate) and for the whole definitional system of Book V of the *Elements* on the theory of proportions. It is still impossible, however, to account for them in a synoptic survey like this one, and the reader is simply warned that definitions may play a supplementary role in the system of principles that does not always appear in an entirely evident manner in the list of axioms and postulates. In order, then, to follow the developments consisting in the important attempts to prove the Parallel Postulate or to reform the Eudoxian theory of proportions, further references may be required.

I have also omitted a small number of principles which are to be found in a few encyclopedic works on mathematics which also contain a section on the *Elements* inasmuch as these principles are irrelevant from a foundational point of view. Some of them are simply logical or metaphysical statements, and have no mathematical meaning. For instance, it is not uncommon in some Scholastic works to find stated, as an axiom, the Principle of Contradiction, or the Aristotelian claim that mathematics is a discipline abstracted from sensible magnitudes, or the claim that it is possible to consider the surface of a thing while abstracting from its body. Some other principles are properly mathematical, but have no foundational meaning. For instance, a few handbooks list, among the arithmetical axioms, certain principles explaining the working of the positional notation of numbers. These kinds of principle may be important for the history of notations and mathematical culture, but their relevance is one that lies far wide of the aim of the present essay. An example of both kinds of principles is to be found in the *Arithmetica perfecta* by Georg Henisch (1609, not included in the following list of mathematical editions), which contains sixteen axioms and seven postulates for arithmetic, none of which has anything to do with the theory of numbers in the modern sense; the first axiom states that numbers are potentially but not actually infinite, the third that a number cannot perform any action, the seventh that the natural order of numbers is from the left to the right, the ninth that zero means nothing in itself but adds much value to another number if it is placed at the end of it, while the second postulate states that it is possible to distinguish a whole number from its decimal part by placing a comma in the right place. Interesting as they are, I have excluded these kinds of principle from my list. It should always be remembered, in any case, that a mathematical treatise of the Renaissance or of the Early Modern Age (even an edition of Euclid) may include an assortment of topics which look very odd from a contemporary perspective.

In listing the various principles, I have divided them into thirteen different classes, in accordance with their different mathematical meanings. It should be noted that this division was only carried out in order to facilitate the comparison between principles and has no roots in the real historical development: The actual classification and grouping of principles in the various Euclidean editions is given under each entry, and this classification and grouping is normally only concerned with a division in books and between postulates and axioms. My own arrangement is loosely based on Hilbert’s work on the foundations of geometry (which offers a classification of principles that is still useful and fully perspicuous), but I was obliged to incorporate a few changes to Hilbert’s list in order to accommodate the incredible variety of ancient axioms. In particular, it will be noted that Hilbert’s “axioms of order” are absent from our list: This is an important feature of ancient and early modern mathematics which never thematized relations of order as such. These began to be studied in projective geometry and had to wait until a still later period to be incorporated into an axiomatic system. Hilbert himself attributed the introduction of this kind of axioms to Pasch (1882), and this event should be regarded as an important breakthrough in the history of the foundations of geometry; Pasch’s axiom is nonetheless hinted at in a few early modern axiomatizations (see below). To facilitate a comparison with the modern discussion on axiomatics, I include Hilbert’s system of axioms in the *Grundlagen der Geometrie* (1899, with a few revisions in the following editions) after the other lists of principles. For Hilbert’s own historical sources, see Hallett and Majer (2004), and Volkert (2015) (which also offers a short history of geometrical axiomatics).

A few remarks are in order to explain some of the principles and their classification.

#### Ancient principles (Groups 1 and 2)

The Euclidean list of five Postulates and five Common Notions follows Heiberg’s edition and finds its main foundation and justification in a number of comments made by ancient scholars, who had in their hands editions of the *Elements* with lengthier systems of principles but claimed that some of them had been added in the centuries following Euclid. The additional principles are here listed as P6* and CN6*–CN9*. Axiom CN8*, in particular, is attributed by Proclus (*In Euclidis* 197) to Pappus (along with M1 and M2, see below). Besides the principles which can be found in the Greek manuscripts of the *Elements*, we also mention a few other axioms and postulates which are explicitly stated in ancient commentaries and which clearly influenced the following editions, as well as a few important variants of these. Among these axioms and postulates, the extra assumptions EX1, EX2 and EX3 are attributed by Proclus (*In Euclidis* 198) to Pappus as well, and traces of them are evident in several further axioms, as well as in the definitions of a straight line and a plane; they are, however, nowhere formulated as axioms in a system of principles (even though Albert the Great mentioned them as possible additional axioms in his commentary to Euclid, see Tummers 2014). Other ancient assumptions which *did* find their way into medieval or early modern axiomatizations will be listed under the respectively appropriate category.

A few postulates changed their formulation in different editions of the *Elements*. In particular, Postulate 2 on the extendibility of a straight line states, in its original form, that the straight line may be produced *continuously* . Already in the translation of Adelard of Bath (from the Arabic), however, the postulate is altered so as to make it into a statement about the extendibility of a straight line to *an arbitrary length* (“*assignatam lineam rectam quantolibet spacio directe protrahere*”), dropping the aspect of continuity and stressing rather the length of the extension. In Gerardo da Cremona we also find a version stating the *indefinite* extendibility of the line, the original meaning of which may have been the same as that conveyed by Adelard’s translation. Both versions of the axiom were to be much used in the Renaissance and the Early Modern Age. The modified statement was employed by Campano, and it is common in the tradition stemming from him (in Tartaglia, for instance, or in some Jesuit textbooks), while the Greek formulation is to be found in Zamberti, Grynaeus, Commandino, Clavius and other authors more attentive to philological qualms. This notwithstanding, the two traditions mixed very soon, and it is not uncommon to find the statement about indefinite extension also in editions translated from the Greek; early instances of this attitude are the *Elements* edited by Xylander, Forcadel and Grienberger (who was normally copying his principles from Clavius), but many more may be found in the seventeenth and eighteenth Centuries. I have not signaled these oscillations, as the various spelling of the principle were clearly considered as equivalent, and in a few volumes their equivalence is in fact explicitly stated. It should be remarked that the version of P2 stating the *indefinite* extendibility of the line was sometimes transformed into a claim regarding the *infinite* extendibility of it (instances are Herlin & Dasypodius, Richard, Arnauld and others). Even though the latter spelling may have played a role in establishing the modern non-constructivist theory (i.e., that straight lines are just infinite), the early modern authors that employed the word “infinite” in this connection always added that it was a potential infinite (or a “syncategorematic infinite,” following Richard’s Scholastic wording), and therefore it could be equated with the standard notion of an “indefinite” extension.

The most important geometrical principle added in Antiquity, however, was the one stating that two straight lines do not enclose a space (P6*). This axiom is often stated in this form, even though it is also found from time to time (and more frequently in the Early Modern Age) as the simpler statement that two straight lines have at most one point in common (I17; the first instance of it seems to be in Arnauld, 1663), or a variation of the latter (e.g., I31, I32). While principles P6* and I17 are not equivalent in general (in elliptic geometry I17 obtains and P6* does not), the counterexamples were too abstract to be made specific note of by early modern mathematicians, who regarded the two axioms as interchangeable.

#### The theory of magnitudes (Groups 3 and 4)

I include under this heading several principles which are obvious extensions of the Euclidean Common Notions, and which deal with mereology, mereological addition and subtraction, equality, and relations of order among magnitudes. These principles were often looked upon as the main axioms of the *mathesis universalis*, that is to say, of a mathematical theory which aspired to encompass both geometry and arithmetic (or, in other words, a theory of both continuous and discrete quantities). Many of them were later to be applied in the theory of proportions, which has also been conceived by some authors as a form of universal mathematics. A number of them are especially connected with the arithmetical axioms and may in fact be conflated with them; the reason is that numbers themselves were often conceived of as collections of unities (this is the Euclidean definition in *Elements* VII, def. 2), and therefore the rules of composition and division of numbers were stated as mereological operations (cf. *Elements* VII, def. 3–4). The axioms which I have listed under the category of the theory of numbers (Group 7) should therefore be integrated with the principles regarding sum, subtraction and multiplication of magnitudes which are also presented here.

Many of the principles listed here concern the transitivity of some relation or operation (taking as a model CN1), and usually aim at establishing an equivalence relation of some kind. Other principles are directly about the substitutivity of equivalent magnitudes in several contexts (see especially A18, A19). Still other principles are aimed at providing an ordering structure for magnitudes (although these should not be confused with the missing *geometrical* axioms of order), and, to this effect, there were also conceived and stated certain axioms on trichotomy (see A3, A21, A24).

A corruption of the medieval text appears to be the insertion of the Common Notion A1, which seems to be a useless reduplication of CN1. This first appeared in Adelard’s version, which, in turn, lacks axiom CN8*; it is very likely that a trivial translation slip from the Greek into the Arabic, to be found in the Al-Hajjāj tradition, changed the original CN8* into A1 (such a mistake is probably absent from the Isḥāq ibn Ḥunain translation, and therefore also from Gerardo da Cremona’s Latin version, which was drawn from this Arabic source). The mistake, however, spread in the whole tradition following Adelard (Robert of Chester, John of Tynemouth, Campano da Novara). The spelling of CN1 and A1 is in fact slightly different, but it is hard to figure out a proper difference in meaning: in Adelard’s prose, CN1 reads “*Si fuerint alique due res alicui rei equales, unaqueque earum erit equalis alteri*,” while A1 reads “*Si fuerint due res alicui uni equales, unaqueque earum equalis erit alteri*”; Campano changed the formulation, and his CN1 is “*Que uni & idem sunt equalia, & sibiinvicem sunt equalia*,” while his A1 is “*Si fuerint due res uni equales ipse sibiinvicem erunt equales*.” Luca Pacioli, in 1509, tried to preserve the received text and explained the difference between the two axioms in terms of their being an abstract and a concrete version of the same principle. In 1543, Niccolò Tartaglia simply eliminated A1 from the list of axioms, reintroducing CN8* (following, in this one instance, Bartolomeo Zamberti’s translation from the Greek, which had not suffered from the Arabic textual corruption). On the other hand, Pierre Herigone’s principle A15, stating that things equal to equal things are also equal to one another, may be regarded as a axiom different from CN1, as it expresses a more complex relation than simple transitivity (i.e., if $a=b$ and $c=d$ and $b=d$, then $a=c$). The same holds for Herigone’s M20 and M21 compared with CN8*, and M23 with CN9*.

Axioms M1 and M2, added by Gerardo da Cremona, are taken from An-Nayrīzī’s commentary; Proclus (*In Euclidis* 197) attributes them to Pappus. Principle A4 is almost classical and will be repeated very often in the Euclidean tradition, but it is first stated as an axiom in Bradwardine’s geometry.

It may be remarked that many of the principles under this heading were introduced during the Renaissance (by Lefèvre, Commandino, and Clavius), and that they were in fact among the first newly conceived axioms for the *Elements*. Herigone also contributed much to this category, in an attempt to completely formalize the relations of equivalence. In many respects these principles may be considered to constitute the prehistory of the attempts later made toward an axiomatization of algebraic relations.

#### The theory of proportions and the Axiom of Archimedes (Groups 5 and 6)

The theory of proportions, to be found in Book V of the *Elements*, is probably that part of elementary mathematics which has undergone, in the history of the Euclidean corpus, the greatest amount of modification. It was entirely based on a complex system of definitions, possibly conceived by Eudoxus, which suffered serious textual corruptions during the Middle Ages, and which affected all the medieval Latin translations depending on Adelard (including Campano): see Murdoch (1963) and Molland (1983) for a first survey. On the other hand, an entire new theory was developed in the Arabic world (from a few hints in classical sources), the so-called anthyphairetic theory of ratios, which however (as far as I know) was never axiomatized and reduced to principles (see, for instance, Itard 1961). The original meaning of the theory expounded in Book V of the *Elements* was recovered during the Renaissance, and the restitution of Euclid on this topic was in fact the aim of many philological editions. Once the text was established, several criticism were raised against it, as its meaning remained for a long time quite obscure. Later on, it was widely accepted and began to be regarded as the highest point of the mathematical construction of the *Elements*. In fact, the theory of proportion was adopted as the main mathematical tool to express the laws of Nature at the dawn of the scientific revolution. This, in turn, produced several attempts to reform the Eudoxian theory so as to adapt it to the needs of the new science, and in the first half of the seventeenth century many new principles were envisaged in order to fashion theories of ratios which were grounded on very different foundations. The theory gradually waned in the second half of the century, when algebra and analysis took its place as the tools with which Nature might be mathematized.

Given that the theory was originally founded on a system of definitions, many of the further efforts were also aimed at a reformation of the Euclidean definitions, and the list of axioms presented under this heading do not faithfully represent the actual debates on the matter. The first mathematician to substitute axioms for definitions in the theory of proportions was Francesco Maurolico (in 1567), whose work remained unpublished but probably exerted a certain influence on further studies thanks to its manuscript circulation. We know, for instance, that Clavius studied Maurolico’s notes (even though this is not apparent in Clavius’s own work on the theory of proportions), while we find traces of Maurolico’s axiomatization in Giovan Battista Benedetti (1585), who in turn had a strong influence on Galileo and his school. Borelli himself gained access to Maurolico’s manuscripts before writing his *Euclides restitutus* (1658). It will be noted, in any case, that most of the axioms in the theory of proportions come from editions produced in the second half of the seventeenth century (i.e., after Galileo’s death), when the theory itself had but very little foundational significance as it had been already superseded by more advanced developments in analysis and algebra. These axiomatizations may nonetheless stand as testimony to the fact that the axiomatic approach (here contrasted with the definitional approach) to the foundations of mathematics was, by the end of the seventeenth century, on the point of triumphing. It should also be noted that many among the first propositions of Book V of the *Elements* were employed as axioms; the general idea was that they were immediate consequences of the difficult, or debatable, Eudoxian definitions and (in this case) could perhaps be substituted for them as more perspicuous principles. Thus, even if we may regard some of the new foundations of the theory of proportions as very remarkable logical accomplishments, it is difficult to find any single axiom of the theory which might be considered as a substantial progress as regards formalization. We should, however, mention the axiom of the existence of the fourth proportional (R1, R17, R25), which was implicitly employed by Euclid in proving *Elements* V, 18, and engendered a wide debate on the very notion of mathematical existence and existential assumptions (for instance, Clavius claimed that a fourth proportional to three given magnitudes has to exist, since its existence does not entail any contradiction—thus equating mathematical existence and logical consistency). The missing principle had been already noticed in the Middle Ages, and Khayyām, for instance, had attempted to prove it (see Rashed and Vahabzadeh 1999, pp. 350–351).

Strictly connected with the theory of magnitudes and the theory of ratios is the so-called Axiom of Archimedes, and the notion of Archimedean quantities, which was first formulated by Euclid (or by Eudoxus) as a foundational definition in the theory of ratios (*Elements* V, def. 4), and later also stated, in a different version, as an assumption  of Archimedes’s *De sphaera et cylindro*. This principle was introduced among the axioms of the *Elements* by Commandino (axiom AA1) and was re-stated in several other editions. Its immediate application in a geometrical context was the debate on the angle of contact that had classical sources (Proclus, Simplicius) but especially flamed in the Renaissance and the Early Modern Age, when Clavius, Wallis and many others discussed whether a curvilinear angle (which has no finite ratio with a rectilinear one) might still be considered an angle (and an array of related questions). A few decades after Commandino’s axiomatization, in any case, the theory of indivisibles was developed in Italy (by Cavalieri, Torricelli and others), and these non-Archimedean quantities gave rise to a further, wider debate on their admissibility in mathematics; still later, similar concerns were raised about the infinitesimals of the new Calculus. The debate itself, then, on the Archimedean Axiom is mainly to be found outside of the domain of elementary mathematics, and many eighteenth-century treatises on analysis deal with the topic. Nevertheless, it continued to contribute to enlarging the number of the axioms in the *Elements* in various forms. Indeed, among the editions of the *Elements*, the only (very partial) axiomatization of a theory of non-Archimedean quantities is to be found in the edition of the *Elements* produced by Bernard Lamy (1685), and perhaps in the unpublished papers by Roberval, whose axioms I18–I21 (that I have arranged in Group 9 following their most immediate meaning) might also be read as a foundation of a theory of non-Archimedean magnitudes.

#### The principles of arithmetic (Group 7)

As is well known, Euclid included no principles at all (except definitions) in the arithmetical books of the *Elements*. The reasons for his treating arithmetic differently from geometry in this way are hard to envisage precisely. My guess is that the epistemology of Euclid’s age neither required nor allowed the use of subject-specific axioms in any form. The notion of this age was that a perfect science ought to be grounded in definitions and general logical laws alone (that may include laws on quantity, i.e., “common notions”) without recurring to any further specific principle. Postulates were allowed in the geometrical books to answer a few sophistical objections and were intended to warrant those auxiliary constructions which were needed in the proofs, but since the arithmetical proofs were considered to have no need of any preparatory , and to be susceptible of being carried out in a purely demonstrative way (that is, through a simple ), no postulates were added in Book VII (note, however, that a few propositions of this book require, indeed, that the mathematician is allowed to *take* or *construct* certain numbers; from these few instances, the notion of an arithmetical postulate will later arise). In sum, the very possibility of arithmetic-specific axioms was inconceivable to Euclid (or was, at least, considered by him to be simply wrong), and their first appearance became a possibility only in the late Hellenistic age, if only in the form of assumptions that should (and could) be proven through a further analysis of the matter. In any case, it must be stressed once again that the ancient conception of a system of axioms was very different from our own, and that the need for principles in a given domain was not seen, in Antiquity, as a logical necessity (to rule and define such domain), but rather as a shortcoming characteristic of an imperfect science which needed to make assumptions in order to justify its development; from this point of view, it seems clear that no Greek could ever have had the sense that anything was missing from the arithmetical books of the *Elements* simply by reason of the fact that they contained neither axioms nor postulates.

Be it as it may, however, the conspicuous absence of arithmetical principles in the *Elements* had paramount consequences for the later history of mathematics and epistemology. Whereas the axiomatization of geometry was, in fact, discussed for centuries and hundreds of new principles were invented and tested for it, the foundations of arithmetic remained an issue which was almost entirely neglected until the great formal constructions of the nineteenth century (by Dedekind, Frege and Peano, for instance), with geometry remaining during all this preceding period the grounding discipline for the whole of mathematics. We may recall that Kant himself, in the *Critique of Pure Reason*, still attempted (at the very end of the eighteenth century) a philosophical justification of the fact that geometry has axioms, while arithmetic has no proper principles of its own (*KrV*, A163–165/B204–206). These common epistemological views, which in many respects were just the outcomes of a mathematical tradition (possibly consolidated by the loss of the arithmetical axiomatizations of Late Antiquity), were a further cause of the long delay in the discovery and outline of a transparent structure for the theory of numbers.

It should not be thought, however, that attempts at moving toward an axiomatization of arithmetic were completely absent from the Early Modern Age or in the tradition of Euclid’s *Elements*. In fact, the first printed volume of the *Elements* (1482) was based on the medieval edition of Campano da Novara (thirteenth century), who had, in his time, depended on several other Arabic sources. Campano’s crucially important edition prefaced Book VII of the *Elements* with four postulates and ten common notions bearing on arithmetic, thus constituting a first attempt at an axiomatization of the theory of numbers. When, a few years later, a sounder text of Euclid’s *Elements* was translated into Latin by Bartolomeo Zamberti—which translation, of course, being based this time on a Greek original, presented no principles in Book VII—the new humanistic environment of the Renaissance was quick to reckon the interpolated arithmetical axioms as examples of the barbarisms of a dark age; the greater part, therefore, of the subsequent editions of Euclid (up to the age of Kant) simply stuck to philology and ignored these principles. This notwithstanding, Campano’s edition, which was so important in the mathematical studies of sixteenth century, proved able to assert itself in foundational research. The important editions of Euclid by Commandino and Clavius followed the Greek text (rather than Campano’s Arabized Latin) but found it useful, for foundational purposes, to interpolate Campano’s arithmetical axioms into Book VII. Even if their choice was not followed by several other important editions (which eschewed any textual addition), a minority tradition regarding the *Elements* continued to include arithmetical axioms, and these were sometimes discussed, modified, extended or reduced depending upon the particular foundational opinions of the mathematical editor. In this way, a persistent practice of axiomatic discussion on the theory of numbers was established and, although this discussion remained far less developed than that regarding the foundations of geometry, an unbroken line may be observed to run from Campano down to those important contributions of the nineteenth century which, in many respects, grounded contemporary foundational research.

We may well ask, however, what were Campano’s own sources. It is easy enough to establish that Campano, who was more a compiler than a translator, took his fourteen principles of Book VII from two different sources: the *Arithmetica* of Jordanus de Nemore (a treatise which is not immediately connected with the Euclidean tradition) and the translation of the *Elements*, from the Arabic, of John of Tynemouth (both thirteenth century).

Jordanus’s life, education and culture (even his nationality) are unknown, and so are his sources (on him, see Høyrup 1988). He was, however, undoubtedly a very good mathematician and may have invented his system solely with a view to providing principles for arithmetic. Two of his principles are already to be found in Proclus (whose work Jordanus probably could not have known, since Proclus’s commentary to Euclid is believed to have been rediscovered in the West only at the end the fifteenth century, and to have not been know in its entirety to the Arabic world); these, however, are just interpolations from the text of Euclid (see below), and Jordanus may have re-interpolated them as Proclus (or someone else) did before him. A few others of Jordanus’s principles have undoubtedly a Greek flavor, but some others are decidedly formulated in medieval terms (one of them, for instance, refers to the medieval notion of *denominatio*). In any case, it is very unlikely that Jordanus could have had access to any Greek text, and we are obliged to hold that, if he had sources at all, they were probably Arabic. But whatever Jordanus’s sources were (if any), it seems likely that he had modified and refashioned them so as to better fit his own use of them and his own conceptual instruments; we cannot say for certain whether he himself conceived the idea of introducing axioms into the domain of arithmetic, or whether found it in an older text. It should lastly be noted that Jordanus fashioned a few arithmetical definitions as well that Campano added in his *Elements*.

Tynemouth’s sources, on the other hand, are relatively clear: The arithmetical axioms given by him all come from the Arabic commentary on the *Elements* (which was not itself an edition of Euclid) authored by the Persian mathematician and astronomer An-Nayrīzī (tenth century), who had had, in turn, some access to Heron of Alexandria’s lost commentary on Euclid. We do not know whether Heron had provided a proper set of axioms for arithmetic: From An-Nayrīzī’s wording, it seems rather that he simply took note of a few assumptions which seemed necessary in order to complete this or that proof of Euclid’s, and he may even have proven these further assumptions (see Vitrac 1990–2001, vol. 2, pp. 292–296); from Proclus (*In Euclidis* 196), we know that Heron had in fact *reduced* the number of the Euclidean common notions to three, and it would be strange if Proclus did not mention in this connection Heron’s addition of several arithmetical axioms, if indeed this had really occurred. It is possible, but unlikely, that an Arabic edition of the *Elements* had already inserted these assumption into the Euclidean text labeling them as axioms of Book VII. In any case, An-Nayrīzī’s commentary had already been translated into Latin by Gerardo da Cremona in the twelfth century, and Tynemouth may have been able to interpolate these Heronian assumptions, as proper arithmetical *axioms,* into his edition of the *Elements*—something that had not been done by either An-Nayrīzī, or Gerardo. In this way, Tynemouth inaugurated the modern practice of regarding the Euclidean theory of numbers as an axiomatic domain.

The question of a possible Greek axiomatization of arithmetic is, in any case, relevant indeed. No Greek arithmetical work (such as Diophantus’s, for instance) has come down to us which explicitly mentions any principle besides the definitions. Even if, of course, the greater part of the ancient mathematical texts are lost, this may at first appear to be a confirmation that Euclid’s practice in Book VII was followed by the entire Greek tradition. There has come down to us, however, a (highly problematic) contrary instance in Proclus. The Neoplatonic philosopher, in his commentary on Book One of Euclid’s *Elements*, discussed several opinions regarding the basis of the distinction between axioms and postulates. One of them consisted simply in the contention that axioms (or “common notions”) are principles shared by all mathematical sciences, and thus by both geometry and arithmetic in particular (that is to say, they form the principles of what will be later called the *mathesis universalis*). Another way of spelling out the difference between axioms and postulates, however, is that axioms express states of affairs (e.g., “all right angles are equal”), while postulates license constructions (“to draw a straight line between two points”). If the latter distinction is accepted, Proclus said, we may still conceive that there are general principles of a *mathesis universalis*, and these will be, in their turn, distinguished into general axioms and general postulates, and we will also have specific geometrical postulates and axioms, and specific arithmetical postulates and axioms. Proclus (*In Euclidis* 184) gave as examples of these arithmetical principles such propositions as “Every number is measured by the number one” (axiom), and “A number can be divided into least parts” (postulate). This is possibly the only mention of arithmetical principles that is to be found in the whole Greek mathematical corpus.

It is not easy to understand the actual historical relevance of Proclus’s remark, or whether it should be considered as a piece of evidence for the existence of a well-developed (but now lost) system of axioms for arithmetic already in ancient times. On a first reading, this seems not to be the case: Proclus’s argument here is clearly dictated by a concern for symmetry (if geometry has postulates and axioms, then arithmetic must have its own as well) and may just represent the philosophical hobby-horse of a metaphysician trying to introduce some order into certain disordered mathematical writings which did not fit well with his elegant epistemological design. If this is the case, we should not expect to find that any Greek mathematician ever developed an axiomatic approach to arithmetic. And yet the examples that Proclus gives are grounded in real mathematical needs: His axiom is an assumption (explicitly) taken for granted by Euclid in *Elements* VIII, 6, while his postulate is assumed in the proof of *Elements* VII, 31. They thus appear to enjoy the same status as those other principles which, already in ancient times, were extrapolated from Euclid’s demonstrations, where they appeared as unwarranted, and transformed into axioms (for example, the axiom that “two straight lines do not enclose a space”, which Proclus himself considered spurious, seems to have been taken from a similar statement appearing in the proof of *Elements* I, 4). It would be odd if Proclus, when inventing two principles exemplifying the existence of arithmetical axioms and postulates, had happened to hit upon exactly these propositions; it seems more likely that he was taking them from an existing edition of the *Elements*, or a commentary thereon, where they had already been inserted as principles of the arithmetical books. It is hard to guess at Proclus’s own source (if any) in this respect: It might have been Heron himself, but several other sources, Hellenistic, Roman, or belonging to Late Antiquity, are also possible. Odd as it may seem that we find no further trace of arithmetical principles in any of the extant mathematical works of Antiquity, we do at least find, for the reasons given, a hint in Proclus that an axiomatization of arithmetic (be it in a systematic or unsystematic form) had been attempted already in ancient Greece. We know that Proclus had planned, and perhaps realized, a commentary on the other books of the *Elements*, and it is possible that much more information about these arithmetical principles was offered in these projected or lost commentaries, but we cannot say more (we have no extant commentary dating from antiquity on *Elements* VII, 31 and VIII, 6). But it should be clear, in any case, that in Proclus’s times (and possibly already in the age of Heron) mathematical epistemology had already changed, with respect to Euclid, to such an extent as at least to allow the possibility of an arithmetical axiomatization.

As for those principles themselves which were envisaged in the centuries after Euclid, their relevance is very obvious, and even if they do not attain to (nor aim to attain to) the simplicity and effectiveness of Peano’s axioms, they nonetheless formalize a vast array of those properties involved in basic operations on numbers, often with a very high degree of logical abstraction. It has already been noted that they should be read together with most of the general principles on the mereology of magnitudes (Groups 1–4).

#### Principles on space and situation (Group 8)

I have placed under this heading a few very general principles which are more philosophical than mathematical but which nonetheless enjoy an important geometrical significance. Many of them come from Francesco Patrizi’s *Della nuova geometria* (1587) and Pascal’s *Introduction à la géometrie* (1655). The importance of these two works consists in their claim that geometry should be regarded as the science of space (S1, S7), a contention which represented a rather new epistemological position in the sixteenth and seventeenth Centuries. Since the days of Eudoxus and Aristotle, throughout antiquity and for a long time afterward, the science of geometry had tended to identify its proper and specific object rather as the notion of *magnitude,* or continuous quantity. As a consequence, spatial notions tended not to be represented in classical mathematical works and are not employed in the *Elements*. This situation persisted on into the Middle Ages, and indeed even on into the Renaissance and the Early Modern Age; the axioms of Groups 2–6 bear witness in fact, to the preeminence of the notion of magnitude over any other general concept. The new view that it was the proper and essential task of geometry to investigate space and spatial relations is a view which is first to be found in Patrizi’s work (a book on elementary geometry which is otherwise unremarkable from the mathematical point of view); several decades later it resurfaces in Pascal’s unpublished introduction to geometry, which had been, in its turn, inspired by the introduction of a few spatial terms into the mathematical treatises on perspective. Pascal’s work influenced Leibniz’s attempt to establish a new geometry of space, which the latter named *analysis situs*; finally, this Leibnizian project was among the motivating factors behind a proper development of spatial notions in late-eighteenth- and nineteenth-century geometry. For a survey of this important transformation of geometry, see the collection of essays published in De Risi (2015a); on Patrizi’s new geometry, see in particular (De Risi 2014).

It should be noted that projective geometry and non-Euclidean geometry are simply inconceivable without the notion of a geometrical space, and several principles grounding modern geometry in the nineteenth century cannot but refer to spatial or positional concepts (including the above-mentioned axioms of order). The editions of the *Elements* which we consider below do not show evidence of these later outcomes, which fell outside of the domain of commentaries on Euclid, and were largely devised at periods situated some years beyond the chronological boundary we have set. Nevertheless, it is important to observe how spatial notions were indeed employed in a number of works on the foundations of geometry well before the nineteenth century. In particular, we may note that the notion of *situation* (Latin: *situs*), meaning the basic spatial relation obtaining between two objects, is the main tool used by Pascal to build up his own axiomatics (Patrizi also employed it in his definitions of geometrical entities). This notion was derived from the Euclidean treatise on *Data*, that is, from one of the very few essays belonging to ancient mathematics which (very briefly) discusses a spatial concept (i.e., *position*, ). The latter work had been translated into Arabic during the Middle Ages, and into Latin (from the Arabic sources) by Gerardo da Cremona; another Latin translation, made directly from the Greek, circulated in the Middle Ages, and was later superseded by Zamberti’s translation; the first important editions, though, came only with Hardy (1625) and Gregory (in his Euclidean edition from 1703).

We may also note that, in this Group of axioms, a number of principles also state the tridimensionality of space, an axiom which had been never explicitly assumed before the late Renaissance.

#### Principles of incidence and continuity (Groups 9 and 10)

Dimensional considerations are nonetheless central in the axioms of incidence, since these latter deal with the intersections of various geometrical figures. The axioms in this category are among the most interesting of our survey, and have survived in more intact forms into modern axiomatizations (such as Hilbert’s). Their importance lies in their characterizing very basic geometrical entities, such as a straight line and a plane, the geometrical behavior of which had hardly been adequately captured by ancient or early modern definitions; in fact, in the absence of exact definitions of these notions, the axioms here in question formed the true principles employed in the proofs. While the origin of a number of these axioms is to be found in the Greek commentaries, many more were added by Clavius (who achieved important advances in this connection), and by Borelli, who introduced several axioms which were later to be employed by many other editions.

We may note that Clavius’s axioms I5 (“*Two straight lines do not have a common segment*”) is from time to time also spelled as “*Two straight lines which coincide in a segment, will coincide in their entirety (if extended far enough)*,” which is, however, an equivalent formulation of the former.

It should also be noted that, contrary to Hilbert’s neater axiomatization, the present category has no clear boundaries, and a few principles classed here might well also have been classed among the principles of congruence: see, for instance I10, I24, or the series of axioms by Lamy regarding perpendicular lines; postulate I4, which is a simple extension of P2 to a higher dimension, is not, properly speaking, a principle of incidence either; nevertheless, it may legitimately be placed here since it expresses the indefinite (or infinite) extension of planes (it may also be regarded as a specification of EX1). The lack of sharp boundaries is especially evident with respect to the group formed by the principles of continuity, some of which can be distinguished only with difficulty from those of incidence; my basic criterion of distinction here was to include all principles which dealt with intersections with bounded figures among those of continuity (the only exception being C11).

Rather than assuming a single all-encompassing continuity principle (as did, for example, Hilbert) early modern axiomatizations attempted to solve the problem of spatial connection by means of a vast array of properly geometrical principles. Several of them were developed in the course of discussing the proof of *Elements* I, 1, in which a number of mathematicians believed they could identify a demonstrative gap, since Euclid did not prove that two drawn circumferences actually meet in two points. Borelli is, once again, a good example of this kind of discussion, which fills some pages of his *Euclides restitutus*; the first mathematician to conceive an axiom to fill this gap, however, had been Richard in 1645. Other principles were conceived for broader purposes, and in fact they may be considered topological axioms in the proper sense. A French line in the evolution of the continuity axioms started with Fine and was further developed by Richard, Pascal and Roberval. Many axioms deal with the intersections between the boundary of a closed figure and a line which has its extremities inside and outside of the latter figure; some of them may be regarded as early modern forms of the so-called Line–Circle continuity principle, which states that an (infinite) line with a point in a circle cuts the circle in two points. This principle is, in fact, equivalent to the Circle–Circle continuity axiom which is tacitly employed in *Elements* I, 1 in order to establish the assumption that the two circles actually meet (cf. C13). The latter principles are both much weaker than Hilbert’s full continuity axiom, but are still demonstrably strong enough to ground all theorems of elementary geometry, as far as continuity claims are concerned. A perspicuous and historically sensible proof that Hilbert’s axioms of incidence, order and congruence, together with the Parallel Postulate and Line–Circle continuity, are a viable basis for the performance of all the demonstrations of Euclid’s *Elements* is given in Hartshorne (2000). I have placed among the principles of continuity also Roberval’s axiom C18, which is a fairly good statement of that famous Axiom of Pasch (1882) which was employed by Hilbert to complete his axioms of order. Even if it is stated by Roberval himself to be a proposition about continuity and intersections (and already hinted at by Richard in his C9, a version of the axiom more similar to the so-called “Crossbar Principle”), this may well be the only instance of a deep principle of order making its appearance before the nineteenth century. It should also be noted that Pasch’s Axiom is itself equivalent to the plane separation axiom, stating that an (infinite) straight line cuts a plane into two disjoint parts; the axiom was in fact stated in this form by Kästner: see his I38 (which I have listed among the principles of incidence). Note that Kästner also provides a very neat statement of the Line–Circle continuity axiom C20, thus forming a quite remarkable system of principles in this respect.

#### Principles of congruence and equality (Groups 11 and 12)

These axioms are concerned with the equality of measure of different figures and thus with the notion of congruence.

The axioms of Group 11 are especially concerned with the rigid motion of figures and provide a (very partial) view on those innumerable debates regarding the use and abuse of movement in geometry which had already begun in the Arabic Middle Ages but which found their official inception in the West with Pelletier’s criticism (1557) of Euclid’s proof of *Elements* I, 4 by superposition. The group also includes a few postulates which directly allow the reproducibility of a given figure in another place, and may therefore be closely related to the axioms on situation (Group 8), as the latter may also concern the different spatial positions of congruent figures (cf. T5). I have listed principle T15 in this group as well, which states that one may perform constructions keeping the opening of the compass—something that was not allowed by Euclid, but does not increase the deductive power of the *Elements*, as it amounts to assume *Elements* I, 2–3 as an axiom (cf. T13, T22, T26). It may be noted in this connection that I have omitted from the list of Euclidean editions a few geometrical works dealing with issues on constructability and geometrical instruments, such as Giovan Battista Benedetti’s *Resolutio omnium Euclidis problematum* (Venezia, 1553), Georg Mohr’s *Euclides danicus* (Amsterdam, 1672, in both Danish and Dutch; with the addition of the essay *Euclides curiosus*, Amsterdam, 1673), or Lorenzo Mascheroni’s *La geometria del compasso* (Pavia, 1797). All these treatises show how to perform classical Euclidean constructions by means of a compass alone (without straightedge), or with a compass with fixed opening. Despite their clear foundational significance, however, none of them lists any axiom, and their approach to the subject is more practical than axiomatic.

The axioms in Group 12 do not employ constructions or displacements but rather establish abstract criteria for the equality of measure. Clearly, the divide between the two groups is rather artificial, and, looked at from an abstract point of view, they be considered to state very similar propositions; nonetheless, their foundational strategies were clearly different in the Early Modern Age, and I have therefore distinguished between them.

#### The parallel postulate (Group 13)

The last group of principles lists a few alternatives to the Parallel Postulate which were conceived to replace the infamous Euclidean principle (P5). It should be noted that the list is far from complete, as many attempts to demonstrate the Euclidean Postulate were undertaken by making use of different definitions of parallel lines, the most famous of which is that two straight lines are parallel if they are equidistant (a statement itself equivalent to the Postulate, if one assumes the existence of these equidistant lines). Moreover, several other attempts to prove P5 are contained in monographic essays, such as Wallis’s *De postulato quinto* (1663), Saccheri’s *Euclides vindicatus* (1733) or Lambert’s *Theorie der Parallellinien* (1766). Such works are not included in our list of editions of the *Elements*, and often offered still more and different statements equivalent to P5. The present list is thus quite insufficient even to sketch a short history of the attempts to prove the Parallel Postulate. A few considerations on the axioms are nonetheless in order. Axiom PP1 may be regarded as a principle stating the existence of equidistant straight lines, and it shows a good understanding (by Fine) of the fact that the definition alone would be insufficient to this effect unless one could prove (or assume) that equidistant straight lines are possible (something that is equivalent to the acceptance of P5). Axiom PP2 is a restatement of *Elements* I, 30 on the transitivity of parallelism, a property of parallels also depending on P5 and only true in Euclidean geometry. It was first stated by Proclus (*In Euclidis* 371) and later employed many times in the history of the attempts to prove the Postulate. Axiom PP3 excludes asymptotic parallel lines in hyperbolic geometry (which do not share a common perpendicular), thus establishing Euclidean geometry. Axiom PP4, which originates in the Arabic treatises on this topic, establishes the Euclidean character of a (so-called) Saccheri quadrilateral (these quadrilaterals in hyperbolic or elliptical geometry have the base shorter or longer than the opposite side). Axiom PP5 is taken from Clavius’s proof of the Parallel Postulate, which assumed this statement to show that equidistant straight lines may be constructed, and thus Euclidean geometry be true, arguing that the present proposition about the flow of a point was a self-evident proposition. This argument was employed already in the Middle Ages by Arabic-writing authors (and Ibn al-Haytham in particular) and exerted a lasting influence on the attempts to prove P5; explicitly assuming it as an equivalent statement represented a significant step forward toward a full understanding of the subject. Axiom PP6 is a simple restatement of the Parallel Postulate itself, based on the fact that it is possible to prove that, if two straight lines cut by a third line form angles lesser than two rights, the lines will *locally* approach one another; it is thus possible to state P5 in the form of the proposition that, in the latter situation, the lines will also meet. The confusion between the local approach of the two lines (in a neighborhood of the intersection with the transversal) with their global approach was a serious hindrance to the proper understanding of the behavior of hyperbolic lines. Axiom PP7 is a restatement of PP4 on Saccheri’s quadrilaterals in a different form. Axioms PP8, PP10 and PP11 are further variations on the theme of equidistance. Mercator’s Axiom PP9 had some historical relevance, as it may be regarded as the first instance of the so-called direction theory of parallelism stating that parallel lines share the same direction (here: they do not “incline” toward one another) and therefore the property of parallelism has to be transitive (a theory often employed at the end of the eighteenth century and the beginning of the nineteenth to establish Euclidean geometry). Axiom PP12 is a property which had been implicitly employed by the German mathematician Andreas Segner to prove the Parallel Postulate (as it is false in hyperbolic geometry), and which had been explicitly stated as a principle by his disciple Johann Friedrich Lorenz. It may easily be regarded as an alternative statement of PP2 or PP9 on transitivity. “Playfair’s Axiom” PP13 was also well known many centuries before Playfair’s edition of Euclid, but it had been never stated as such in substitution of P5 in a version of the *Elements*. Given the immediate perspicuousness of the statement, it enjoyed an enormous success in the following axiomatizations.

I have also included in this category two ancillary principles, PP14 and PP15, which are *not* equivalent to the Parallel Postulate. The latter is a restatement of Ptolemy’s proof of *Elements* I, 28 (a converse statement of the Parallel Postulate, which is provable without it) based on symmetry, which Richard found to be so general in its application as to deserve to be included among the axioms. The important axiom PP14 is the so-called Principle of Aristotle (since it was vaguely stated in *De Caelo* A 5, 271$^{\mathrm{b}}$30–32), which Proclus employed in his commentary on the *Elements* (*In Euclidis* 371) to prove the Parallel Postulate, and which was later included by Commandino (1572) in the axiomatic system of the *Elements* with the purpose of replacing the latter postulate by a simpler statement. Proclus’s and Commandino’s proofs were, however, flawed, and in fact it is impossible to prove P5 from PP14 alone. Clavius later (1589) attempted to prove PP14 but gave an insufficient demonstration of it, and only Saccheri (1733) provided a complete and direct proof of the validity of PP14 in absolute geometry. This notwithstanding, Aristotle’s Principle may play an important role in the foundations of geometry, as it may be proven that, if one accepts some very basic statements about geometrical continuity (the Circle–Circle continuity axiom, for instance), the acceptance of Aristotle’s Principle is in fact enough to grant the full Axiom of Archimedes (AA1) for geometrical magnitudes; see Greenberg (1988, 2010), and Pambuccian (1994). Lastly, it may be remarked that the Axiom of Archimedes is also directly involved in the equivalence of the above-mentioned principles, which aim to substitute for P5; for instance, PP4 and PP7 on Saccheri’s quadrilaterals are only equivalent to P5 if AA1 (or PP14 and Circle–Circle continuity) are also assumed; the latter fact was, however, only discovered by Dehn at the beginning of the twentieth century and was never referred to in early modern treatments of this topic.

In translating the principles into English, I generally followed the particular formulation which was given to them in their first historical occurrence. This may result in some slight deviation in terminology in related principles introduced by different authors (for instance, someone may say that a magnitude *measures* another magnitude, while someone else may say, in a related principle, that a magnitude *divides* another magnitude, or *is a part of* this latter; the meaning is, however, the same). I have not differentiated between principles which only differ from one another as regards the terminology employed but which agree with one another as regards their mathematical meaning. Thus, for instance, I have sometimes conflated “magnitude” and “quantity” when these two terms were used by different authors to express the same mathematical notion. These terminological differences may have, in some cases, important epistemological consequences and are very useful for understanding the general philosophical outlook of a mathematician, or the geometrical tradition to which he belongs. For these further and deeper studies, therefore, the original works should always be consulted.

The principles are listed in the various editions in the same order in which they occur, separated by a comma. A dash means that a single principle in the work is in fact composed of two different principles in my list (e.g., Adelard of Bath has a First Postulate stating that a straight line can be drawn between two points (P1) and can be further extended (P2); this postulate is thus mentioned as P1–P2). Sometimes, a number of principles are added in later sections of an edition of Euclid (for instance, since the Parallel Postulate is only used to prove *Elements* I, 29, many editions do not list it at the beginning, but rather in the middle of the First Book, i.e., after *Elements* I, 28), and these are separated from the others by a semicolon. The symbol (...) after the list of principles means that the author explicitly states that the principles are infinite in number, and that he is only giving a few instances of them. The intended meaning of a few more obscure principles is given in the short commentary to the edition in which they occur for the first time. I have signaled in square brackets those principles which are somehow problematic within the context of the work in which they appear; a fuller explanation is given in the commentary.

## The principles

### The principles of Euclid

*Let the following be postulated:*

1. *To draw a straight line from any point to any point.*
2. *To produce a straight line continuously in a straight line.*
3. *To describe a circle with any center and distance.*
4. *That all right angles are equal to one another.*
5. *That, if a straight line falling on two straight lines make the interior angles on the same side less than two right angles, the two straight lines, if produced indefinitely, meet on that side on which are the angles less than the two right angles.*

1. *Things which are equal to the same thing are also equal to one another.*
2. *If equals be added to equals, the wholes are equal.*
3. *If equals be subtracted from equals, the remainders are equal.*
4. *Things which coincide with one another are equal to one another.*
5. *The whole is greater than the part.*

### Further principles in the Greek tradition

P6*: *Two straight lines do not enclose a space.*

CN6*: *If equals be added to unequal things, the wholes are unequal.*
CN7*: *If equals be subtracted from unequal things, the remainders are unequal.*
CN8*: *The doubles of the same thing are equal.*
CN9*: *The halves of the same thing are equal.*

1. *Magnitudes are susceptible of the unlimited both by way of addition and by way of successive diminution, but in both cases potentially only.*
2. *All parts of a plane, and all parts of a straight line, coincide with one another.*
3. *A point divides a line, a line a surface, and a surface a solid.*

### Additional common notions and the foundations of the theory of magnitudes

1. *If two things are equal to another thing, they are equal to one another.* (Adelard of Bath, twelfth century) [cf. CN1]
2. *Any part is lesser than its whole.* (Jordanus de Nemore, thirteenth century) [cf. CN5]
3. *If two equal quantities are compared to a third of the same genus, they will both be equally greater than the third, or equally lesser, or both equal to the third.* (Campano da Novara, thirteenth century)
4. *The whole is equal to all the parts taken together.* (Bradwardine, fourteenth century)
5. *Things which are different from the same thing are also different from one another.* (Bradwardine, fourteenth century)
6. *The container is greater than the contained.* (Maurolico, 1567) [cf. CN5]
7. *Given any magnitude, it is possible to take a magnitude greater or lesser than it.* (Clavius, 1574)
8. *First is what precedes everything else in its order.* (Patrizi, 1587)
9. *Whole is what has parts*. (Patrizi, 1587)
10. *Every whole is divided into parts.* (Patrizi, 1587)
11. *Every quantum can be divided, i.e., is divisible.* (Patrizi, 1587)
12. *Every divisible thing can be divided into lesser things.* (Patrizi, 1587)
13. *If a thing is greater, or lesser, than another thing, it is also greater, or lesser, than a thing equal to the latter.* (Clavius, 1589)
14. *If a thing is greater, or lesser, than another thing, something equal to the former is also greater, or lesser, than the latter.* (Clavius, 1589)
15. *Things which are equal to equal things are also equal to one another.* (Herigone, 1634) [cf. CN1]
16. *If a magnitude is greater than another magnitude, and the latter is greater than a third magnitude, the first magnitude is also greater than the third.* (Herigone, 1634)
17. *If a magnitude is lesser than another magnitude, and the latter is lesser than a third magnitude, the first magnitude is also lesser than the third*. (Herigone, 1634)
18. *The substitution of equals does not change the equality.* (Herigone, 1634)
19. *The interpretation does not change the equality.* (Herigone, 1634)
20. *The measure is not greater than the measured thing.* (Herigone, 1634)
21. *Every magnitude is such as it is said to be, if it cannot be said otherwise.* (Herigone, 1634)
22. *Parts which are identical with other parts are also identical among themselves.* (Herigone, 1634) [cf. CN1]
23. *If the same quantity, or equal quantities, are divided into a smaller or a larger number of equal parts, any one of the parts taken in larger number will be lesser than any one of the parts taken in smaller number.* (Richard, 1645)
24. *A magnitude which is not greater or lesser than another magnitude is equal to it.* (Schott, 1661) [cf. the intended meaning of A21].
25. *A part of a greater magnitude is equal to a given lesser magnitude.* (Roberval, 1675)
26. *Every magnitude is equal to itself.* (Prestet, 1675) [cf. also A21]
27. *A proposition, taken in one of its meanings, cannot be both true and false.* (Prestet, 1689)
28. *If the legitimate and natural consequence of a proposition is false, the proposition itself is false, and its contradictory proposition is true.* (Prestet, 1689)
29. *A quantity is equal to another quantity when it is neither greater nor less than that quantity.* (Hill, 1726)
30. *A quantity is greater than another quantity when it is neither equal nor less than that quantity.* (Hill, 1726)
31. *A quantity is less than another quantity when it is neither equal nor greater than that quantity.* (Hill, 1726)
32. *If a magnitude is smaller than another magnitude, it is possible to take in the latter one or more parts equal to the former.* (König, 1758) [cf. A25, unpublished at the time]

### Principles on mereological composition and multiples of magnitudes

1. *If unequal things are added to equals, the wholes will exceed one another as the added unequal things.* (An-Nayrīzī, transl. by Gerardo da Cremona, twelfth century) [cf. Pappus in Proclus, *In Euclidis* 197]
2. *If equals are added to unequal things, the wholes will exceed one another as the initial unequal things.* (An-Nayrīzī, transl. by Gerardo da Cremona, twelfth century) [cf. Pappus in Proclus, *In Euclidis* 197]
3. *The equimultiples of the same thing, or of equal things, are equal to one another.* (Jordanus de Nemore, thirteenth century) [cf. CN8*]
4. *Things the equimultiples of which are equal are also equal to one another.* (Jordanus de Nemore, thirteenth century) [cf. CN9*]
5. *If two equals are the halves of two things, the two things are equal.* (Lefèvre d’Étaples, 1496)
6. *If unequal things be added to equals, the wholes are unequal.* (Lefèvre d’Étaples, 1496)
7. *If unequal things be subtracted from equals, the remainders are unequal.* (Lefèvre d’Étaples, 1496)
8. *If two greater things are added to one another, and two lesser things are added to one another, the sum of the greater things will be greater than the sum of the lesser things.* (Lefèvre d’Étaples, 1496)
9. *Any magnitude that measures another magnitude also measures any magnitude that is measured by it.* (Commandino, 1572) [cf. N3]
10. *If a magnitude measures both another magnitude and a magnitude that is subtracted from it, it will also measure the remainder.* (Commandino, 1572) [cf. N1]
11. *Any magnitude that measures two or more magnitudes also measures the magnitude composed by them.* (Commandino, 1572) [cf. N2]
12. *If unequal things are subtracted from equals, the differences will exceed one another as the subtracted unequal things.* (Clavius, 1574)
13. *If equals are subtracted from unequal things, the differences will exceed one another as the initial unequal things.* (Clavius, 1574)
14. *If a whole is the double of another whole, and the subtracted part is the double of the corresponding subtracted part, the remainder will also be the double of the other remainder.* (Clavius, 1574)
15. *If unequal things are added to unequal things, the greater to the greater and the lesser to the lesser, the wholes will be unequal, and in fact the former greater than the latter.* (Clavius, 1589)
16. *If unequal things are subtracted from unequal things, the lesser from the greater and the greater from the lesser, the remainders will be unequal, and in fact the former greater than the latter.* (Clavius, 1589)
17. *If two equals are the doubles of two things, the two things are equal.* (Clavius, 1589) [cf. M4, M5]
18. *If a half is subtracted from a whole, the remainder will be a half; if more than a half is subtracted, the remainder will be less than a half; if one third is subtracted, the remainder will be the two thirds, etc.* (Herigone, 1634)
19. *The double of the greater is greater than the double of the lesser.* (Herigone, 1634)
20. *If a thing is the double of a thing equal to another thing, it is also the double of the latter thing.* (Herigone, 1634)
21. *If a thing equal to another thing is the double of a magnitude, the latter thing will also be the double of that magnitude.* (Herigone, 1634)
22. *The half of the greater is greater than the half of the lesser.* (Herigone, 1634)
23. *If a thing is the half of a thing equal to another thing, it is also the half of the latter thing.* (Herigone, 1634)
24. *If a thing equal to another thing is the half of a magnitude, the latter thing will also be the half of that magnitude.* (Herigone, 1634)
25. *If the parts of a whole are equal to one another, the whole will be a multiple of each part as many times as there are parts.* (Herigone, 1634)
26. *If each part of a whole is the double of each part on another whole, the former whole will be the double of the latter.* (Herigone, 1634) [cf. M14]
27. *Equimultiples of equimultiples are equimultiples to one another.* (Herigone, 1634) [*Elements* V, 3]
28. *A multiple of a multiple of a magnitude is also a multiple of that magnitude.* (Richard, 1645)
29. *If a magnitude measures two other magnitudes, it also measures their sum and their difference.* (Borelli, 1658) [cf. N2 and N1]
30. *If a magnitude is contained a certain number of times in another magnitude, a magnitude equal to the former will be contained the same number of times in a magnitude equal to the latter.* (Borelli, 1658) [cf. N30 and N31]
31. *Equimultiples of two magnitudes to which other equimultiples of the same magnitudes are added or subtracted, are still equimultiples of the two magnitudes.* (Arnauld, 1667) [*Elements* V, 5 and 6]
32. *If a magnitude is subtracted from itself, the result is nothing.* (Prestet, 1675)
33. *Many nothings are equal to nothing.* (Prestet, 1675)
34. *A multiple of a greater magnitude is greater than the same multiple of a lesser.* (Simson, 1754)
35. *That magnitude whose multiple is greater than the same multiple of another is greater than that other magnitude.* (Simson, 1754)
36. *To take any multiple of a given magnitude.* (König, 1758)
37. *The sum of the equimultiples of the parts of a magnitude are equal to the equimultiple of that magnitude.* (Simpson, 1760) [cf. *Elements* V, 1]

### The theory of ratios and proportions

1. *As a certain quantity is to any other quantity of the same genus, so will any third quantity be to a certain fourth quantity of the same genus.* (Campano da Novara, thirteenth century) [existence of the fourth proportional]
2. *Ratios resulting from equal ratios are equal.* (Lefèvre d’Étaples, 1496)
3. *Equal magnitudes have the same ratio to the same magnitude.* (Maurolico, 1567) [cf. *Elements* V, 7]
4. *If two magnitudes have the same ratio to a third magnitude, they are equal to one another.* (Maurolico, 1567) [cf. *Elements* V, 9]
5. *A magnitude has the same ratio to two equal magnitudes.* (Maurolico, 1567) [cf. *Elements* V, 7]
6. *If a magnitude has the same ratio to two magnitudes, the latter are equal to one another.* (Maurolico, 1567) [cf. *Elements* V, 9]
7. *If two magnitudes are equal to two other magnitudes, their ratios are equal.* (Maurolico, 1567)
8. *A magnitude greater than another magnitude has a greater ratio to a third magnitude; and vice versa.* (Maurolico, 1567) [*Elements* V, 8]
9. *Of magnitudes which have a ratio to the same, that which has a greater ratio is greater; and that to which the same has a greater ratio is less.* (Maurolico, 1567) [*Elements* V, 10]
10. *Two ratios which are equal to a third ratio are equal to one another.* (Maurolico, 1567) [*Elements* V, 11]
11. *If equal ratios are composed with equal ratios, the composed ratios will also be equal.* (Maurolico, 1567) [cf. *Elements* V, 22]
12. *If equal ratios are taken away from equal ratios, the remaining ratios will be equal.* (Maurolico, 1567)
13. *Equimultiples of two magnitudes are to one another as the magnitudes themselves are to one another.* (Maurolico, 1567) [*Elements* V, 15]
14. *If unequal ratios are added to equal ratios, the composed ratios will be unequal.* (Maurolico, 1567)
15. *If unequal ratios are subtracted from equal ratios, the remaining ratios will be unequal.* (Maurolico, 1567)
16. *Equal ratios are both equal, lesser, or greater than another ratio.* (Maurolico, 1567)
17. *The ratio that holds between two magnitudes will also hold between a given magnitude and some other magnitude. Moreover, the same ratio will also hold between some magnitude and the given magnitude.* (Clavius, 1574) [a different version of R1]
18. *The ratio of a magnitude to another magnitude is composed by the ratios of the parts of the first magnitude to the second magnitude.* (Benedetti, 1585) [cf. *Elements* V, 12]
19. *If a first magnitude have to a second the same ratio as a third to a fourth, and the third have to the fourth a greater ratio than a fifth to a sixth, the first will also have to the second a greater ratio than the fifth to the sixth.* (Benedetti, 1585) [*Elements* V, 13]
20. *The multiple of a magnitude is to this magnitude as the equal multiple of another magnitude is to the latter magnitude.* (Benedetti, 1585)
21. *The aggregate of all magnitudes in a continued proportion is a multiple of each of the ratios.* (Benedetti, 1585)
22. *Any ratio, divided in any way, is composed by the parts in which it is divided.* (Benedetti, 1585)
23. *If two magnitudes have a different ratio to a third, the greater among them will have a greater ratio.* (Torricelli, 1647)
24. *A ratio is greater than another ratio if the first magnitude of the first ratio is greater than it had to be to have the same ratio to the second as the third to the fourth.* (Torricelli, 1647) [from Galileo]
25. *Given three magnitudes A, B and C, a fourth one, Z, can be given, that has with C the same ratio as A to B.* (Tacquet, 1654) [a different version of R1 and R17]
26. *Two ratios in proportion may have (at least) three terms: if one of them is taken twice as an antecedent, or as a consequent, or one time as an antecedent and the other time as a consequent.* (Borelli, 1658)
27. *If four quantities are such that the first is greater than the second, but the third is not greater than the fourth, the ratio of the first to the second will be greater than the ratio of the third to the fourth.* (Borelli, 1658)
28. *Ratios which have a common consequent, are to each other as are the antecedents.* (Arnauld, 1667)
29. *Different multiples of a magnitude are to one another as the equal multiples of any other magnitude.* (Arnauld, 1667)
30. *Equimultiples of two magnitudes are to one another as different equimultiples of the same magnitudes.* (Arnauld, 1667)
31. *If two ratios are equal, the inverse ratios are also equal.* (Arnauld, 1667)
32. *Two continuous magnitudes F and G have the same ratio as the ratio F to H to the ratio G to H, with a common consequent H. * (Gottignies, 1669)
33. *The double is to the double as the half is to the half, and so for the other multiples and submultiples; and vice versa.* (Fabri, 1669) [cf. R13]
34. *If a magnitude has some ratio to another magnitude, the latter also has some ratio to the former.* (Fabri, 1669)
35. *If a magnitude is substituted in a ratio with an equal magnitude, the ratio does not change; and vice versa.* (Fabri, 1669) [*Elements* V, 7]
36. *If a magnitude is substituted in a ratio with an equal magnitude, but the other magnitude is changed to a different magnitude, the ratio also changes.* (Fabri, 1669)
37. *If a third ratio is greater or lesser than one of two equal ratios, it is also greater or lesser than the other equal ratio.* (Viviani, 1674)
38. *If four magnitudes are in proportion, an equimultiple of the first magnitude is to the second as the same equimultiple of the third magnitude is to the fourth.* (Viviani, 1674) [cf. *Elements* V, 4]
39. *If four magnitudes are in proportion, the first magnitude is to an equimultiple of the second as the third magnitude is to the same equimultiple of the fourth.* (Viviani, 1674) [cf. *Elements* V, 4]
40. *If a ratio is lesser than another ratio, which is itself lesser than a third, the first will also be much lesser than the third.* (Viviani, 1674)
41. *A magnitude has a greater ratio to a certain magnitude than to a magnitude greater than it.* (Mercator, 1678) [*Elements* V, 8]
42. *If magnitudes be proportional separando, they will also be proportional componendo.* (Mercator, 1678) [*Elements* V, 18]
43. *If four magnitudes are in proportion, any equimultiples of the first and the third are both greater, or both equal, or both lesser than any equimultiples of the second and the fourth.* (Rohault, 1682) [cf. *Elements* V, def. 5]
44. *If four magnitudes are such that any equimultiples of the first and the third are both greater, or both equal, or both lesser than any equimultiples of the second and the fourth, they are proportional.* (Rohault, 1682) [*Elements* V, def. 5]
45. *If four magnitudes are such that for some equimultiples of the first and the third and some equimultiples of the second and the fourth it may happen that the equimultiple of the first is greater than the equimultiple of the second but the equimultiple of the third is equal or lesser than the equimultiple of the fourth, the four magnitudes are not proportional, and the first ratio is greater than the second.* (Rohault, 1682) [*Elements* V, def. 7]
46. *All ratios of a magnitude to a magnitude equal to it are equal to one another.* (Arnauld, 1683)
47. *The ratio of a magnitude to another magnitude is equal to the sum of the ratio of a magnitude smaller than the first to the second and the ratio of their difference to the second.* (Arnauld, 1683)
48. *The ratio of a magnitude to the part of another magnitude is greater than the ratio of the first magnitude to the whole second magnitude.* (Arnauld, 1683)
49. *Ratios which have a common antecedent, are to one another as is the inverse ratio of the consequents.* (Arnauld, 1683)
50. *Given four magnitudes in two ratios, three equalities are possible: the equality of the antecedents, the equality of the consequents, and the equality of the ratios. If any two of these equalities are given, the third is also given.* (Arnauld, 1683)
51. *Given four ratios in two proportions, the first ratio of the first proportion is to the second ratio of the first proportion as the second ratio of the first proportion is to the second ratio of the second proportion.* (Arnauld, 1683)
52. *A proportion does not change if the ratios are exchanged among them.* (Arnauld, 1683)
53. *Lines and surfaces the ratio of which is equal to a ratio between numbers are commensurable; otherwise they are incommensurable.* (Lamy, 1685)
54. *If a whole is to another whole as a part of the former is to a part of the latter, the remaining part of the former has the same ratio to the remaining part of the latter.* (Newton, 1706)
55. *Given four magnitudes, if the first is the same equimultiple, or the same part, or the same parts of the second, as the third is to the fourth, the ratio between the first and the second will be equal to the ratio between the third and the fourth.* (Marchetti, 1709) [*Elements* VII, def. 20]
56. *If equal ratios are added to unequal ratios, the composed ratios will be unequal.* (Marchetti, 1709)
57. *If equal ratios are subtracted from unequal ratios, the remaining ratios will be unequal.* (Marchetti, 1709)
58. *Ratios that are double, triple,&c., a half, a third,&c. of equal ratios (or of the same ratio) are equal.* (Marchetti, 1709)
59. *Given four magnitudes in proportion, the first and the third magnitudes will both be greater, or will both be lesser, or will both be equal, to the second and the fourth.* (Marchetti, 1709)
60. *Quantities having the same ratio to another quantity are equal to one another.* (Simpson, 1747) [together with R62, a reformulation of R4]
61. *Equal quantities have the same ratio with the same quantity.* (Simpson, 1747) [together with R63, a reformulation of R35]
62. *Quantities to which another quantity has the same ratio are equal to one another.* (Simpson, 1747) [together with R60, a reformulation of R4]
63. *The same quantity has the same ratio with equal quantities.* (Simpson, 1760) [together with R61, a reformulation of R35]
64. *If two magnitudes are divided into equal parts, the first is to the second as the number of equal parts of the first is to the number of equal parts of the second.* (Simpson, 1760).

### The Axiom of Archimedes and the theory of indivisibles

1. *Any magnitude may be multiplied as many times as to exceed any given homogeneous magnitude.* (Commandino, 1572)
2. *Every solid is formed by an infinite number of parallel planes which have an insensible thickness.* (Lamy, 1685)
3. *If two solids have the same height and the planes that compose them have the same thickness, the two solids are formed by the same number of planes.* (Lamy, 1685)
4. *If a straight line arbitrarily small, and a straight or curved line arbitrarily long (but finite), are given, it is possible to take the first line a number of times such that it will exceed the second line.* (Roberval, 1675)
5. *If two or more quantities differ by a quantity less than any assignable quantity, these quantities will be perfectly equal to one another.* (Deidier, 1739)
6. *It is possible to take a straight line so small that its square is less than any assigned surface.* (Simpson, 1760)

### Principles of arithmetic and number theory

1. *A number that divides a whole and the part which is subtracted from it also divides the difference.* (John of Tynemouth, twelfth century)
2. *Any number that divides two numbers also divides their sum.* (John of Tynemouth, twelfth century)
3. *Any number that divides something also divides everything that is divided by it.* (John of Tynemouth, twelfth century)
4. *To take any quantity of numbers equal to any given number.* (Jordanus de Nemore, thirteenth century)
5. *To take a number however greater than any given number.* (Jordanus de Nemore, thirteenth century)
6. *The series of numbers can be extended to infinity.* (Jordanus de Nemore, thirteenth century)
7. *That part is lesser which has a greater denomination; and that part is greater which has a lesser denomination.* (Jordanus de Nemore, thirteenth century)
8. *The unity is a part of any number, and denominated by it.* (Jordanus de Nemore, thirteenth century)
9. *Every number is a multiple of the unity as many times as the unity is part of it.* (Jordanus de Nemore, thirteenth century)
10. *Every number multiplied by the unity results in itself. The unity multiplied by any number results in this number.* (Jordanus de Nemore, thirteenth century)
11. *The difference of the extremes is composed of the differences of the same extremes to the mean term.* (Jordanus de Nemore, thirteenth century)
12. *To take any quantity of numbers equal to, or multiples of, any given number.* (Campano da Novara, thirteenth century) [cf. N4]
13. *No number can be diminished to infinity.* (Campano da Novara, thirteenth century)
14. *If a number is greater than another, and their difference is added to the lesser, or subtracted from the greater, the resulting numbers will be equal.* (Lefèvre d’Étaples, 1496)
15. *Two numbers that have the same ratio to the same number are equal.* (Lefèvre d’Étaples, 1496) [cf. *Elements* V, 9]
16. *A number may be subtracted from another number as many times as the former divides the latter.* (Lefèvre d’Étaples, 1496)
17. *The greater number does not divide the lesser.* (Lefèvre d’Étaples, 1496)
18. *If a given number is multiplied by another number and then is divided by it, the result is again the given number. If a given number is divided by another number and then is multiplied by it, the result is again the given number.* (Lefèvre d’Étaples, 1496)
19. *If a number measures a part* [i.e., a submultiple]*, it also measures the whole.* (Candale, 1566)
20. *The sum of a given number with a number that is greater than another one is greater than its sum with the lesser number.* (Pedro de Monzón, 1569)
21. *Numbers which are made by the same quantity of unities are equal.* (Pedro de Monzón, 1569)
22. *Two number are equal if their parts with equal denomination are in equal quantity.* (Pedro de Monzón, 1569)
23. *If two number are multiplied, the product is to the first as the second is to the unity.* (Pedro de Monzón, 1569)
24. *If a number divides another number, the divided number is to the divisor as the quotient is to the unity.* (Pedro de Monzón, 1569)
25. *If two numbers are added together which are greater than two other numbers, the sum of the greater ones is greater than the sum of the lesser ones.* (Pedro de Monzón, 1569)
26. *The ratio of a number with another number is the same as the ratio of the parts of the two numbers with the same denomination.* (Pedro de Monzón, 1569)
27. *If a unity is added to an odd number, the result is an even number.* (Pedro de Monzón, 1569)
28. *If a unity is added to an even number, the result is an odd number.* (Pedro de Monzón, 1569)
29. *If several ratios among numbers are all equal to the same ratio, they will also be equal to one another.* (Billingsley, 1570) [cf. *Elements* V, 11; and R10]
30. *Those numbers which are the same part (or the same parts) of the same number (or of equal numbers) are equal to one another.* (Commandino, 1572)
31. *Those numbers, which have the same (or equal) number of the same part (or parts) are equal to one another.* (Commandino, 1572)
32. *The unity divides any number.* (Commandino, 1572)
33. *Every number divides itself.* (Commandino, 1572)
34. *If two numbers are multiplied, the one measures the product by the other and the latter by the former.* (Commandino, 1572)
35. *If a number measures another number by a third one, the third also measures the latter by the former.* (Commandino, 1572)
36. *If a number measures another number by a third one, the product of the first by the third, or of the third by the first, results in the second.* (Commandino, 1572) [cf. N18]
37. *It is possible to make additions, subtractions, multiplications, divisions, extractions of roots, and squares and cubes of numbers.* (Herigone, 1634)
38. *Every property of a number is also a property of a number equal to it.* (Herigone, 1634)
39. *It is the same to multiply a whole with a whole, or a whole with each of the parts, or each of the parts with a whole.* (Arnauld, 1667) [cf. *Elements* V, 1]
40. *No magnitude has a divisor greater than itself.* (Prestet, 1675)
41. *The greatest common divisor of two parts composing a whole is also the greatest common divisor of one part and the whole.* (Prestet, 1675)
42. *The greatest common divisor of a whole and one of its two parts is also the greatest common divisor of the whole and the other part.* (Prestet, 1675)
43. *If a magnitude with the sign + is joined to the same magnitude with the sign –, the result is equal to nothing.* (Lamy, 1685)
44. *Two numbers are always commensurable the one another.* (Lamy, 1685)
45. *If a number is subtracted from another number, the result is a number.* (Lamy, 1685)
46. *It is the same thing to multiply 12 times 8, or 8 times 12.* (Malézieu, 1705) [cf. *Elements* VII, 16]
47. *It is the same thing to multiply 12 times 8, or 12 times many parts which, taken together, are equal to 8.* (Malézieu, 1705) [cf. N39]
48. *If equal quantities are multiplied by equal quantities, their products will be equal.* (Malézieu, 1705)
49. *If equal quantities are divided by equal quantities, their quotients will be equal.* (Malézieu, 1705)
50. *Given a number, to add a unity to it.* (Newton, 1706)
51. *Given a number, to subtract a unity from it.* (Newton, 1706)
52. *If the roots of any two quantities are equal, their squares are also equal.* (Hill, 1726)
53. *If the squares of any two quantities are equal, their roots are also equal.* (Hill, 1726)
54. *If unequal quantities are multiplied by equal quantities, their products will be unequal.* (Deidier, 1739)
55. *If unequal quantities are divided by equal quantities, their quotients will be unequal.* (Deidier, 1739)

### General principles of space, situation, and givenness

1. *Mathematical sciences are not abstracted from natural things; nor are they in the imagination, nor are they in the intellect; but rather, space is their subject.* (Patrizi, 1587)
2. *Geometry takes into consideration the point, the line, the surface, and the body, in this natural order.* (Patrizi, 1587)
3. *Space is extension, and extension is space.* (Patrizi, 1587)
4. *Every space is a minimum, a maximum, or in the middle of these.* (Patrizi, 1587)
5. *Every space is long; or long and wide; or long and wide and deep.* (Patrizi, 1587)
6. *Given a thing, to take in it a point or a straight line.* (Ricci, 1651)
7. *The object of pure geometry is space, which is considered as extended in three dimensions.* (Pascal, 1655) [cf. S1 and S5]
8. *Space is infinite in each dimension*. (Pascal, 1655)
9. *Space is immovable as a whole and its parts are also immovable.* (Pascal, 1655)
10. *Points may only differ in situation.* (Pascal, 1655)
11. *Lines may differ in situation, magnitude and direction.* (Pascal, 1655)
12. *Surfaces may differ in situation, length, breadth, content, and direction.* (Pascal, 1655)
13. *Equal straight lines only differ in situation.* (Pascal, 1655)
14. *Equal circles only differ in situation.* (Pascal, 1655)
15. *Equal arcs of the same circle only differ in situation*. (Pascal, 1655)
16. *Cords of equal arcs of equal circles only differ in situation.* (Pascal, 1655)
17. *If an end of a straight line is given in position, and the straight line is given in magnitude, the other end of the straight line will fall on the circumference of the circle with that given center and radius.* (Arnauld, 1667)
18. *To assume a figure, whose properties are looked for in a theorem, even though it is not constructed.* (Fabri, 1669)
19. *There exists a solid body.* (Roberval, 1675)
20. *A straight line is given in position and magnitude when its endpoints are given.* (Kästner, 1758) [Euclid, *Data* 26; cf. also I30].

### Principles of intersection, incidence and connection

1. *A surface cuts a surface in a line.* (An-Nayrīzī, transl. by Gerardo da Cremona, twelfth century) [cf. EX3]
2. *If two surfaces which cut one another are planes, they cut one another in a straight line.* (An-Nayrīzī, transl. by Gerardo da Cremona, twelfth century)
3. *A line cuts a line in a point.* (An-Nayrīzī, transl. by Gerardo da Cremona, twelfth century) [cf. EX3]
4. *To indefinitely extend any plane.* (An-Nayrīzī, transl. by Gerardo da Cremona, twelfth century)
5. *Two straight lines do not have a common segment.* (Clavius, 1574) [from Proclus, *In Euclidis* 214; cf. I3]
6. *Two straight lines converging into a point will necessarily intersect one another at that point when produced.* (Clavius, 1589)
7. *If a point lies on two straight lines, it will be at their intersection or meeting.* (Herigone, 1634)
8. *If two points are on the same plane, the straight line joining them will be on the same plane.* (Herigone, 1634)
9. *Two concurrent and intersecting straight lines have no common part.* (Grienberger, 1636) [another version of I6]
10. *From any point of a circular arc that is not one of its two ends, two straight lines may be drawn to the ends of the arc, forming a triangle with the chord between the ends.* (Richard, 1645)
11. *Given two points, to draw a circle having one of the points as its center and passing through the other point.* (Ricci, 1651)
12. *Through any line, to draw a plane.* (Borelli, 1658)
13. *Two planes do not have a common* [*plane*]*segment.* (Borelli, 1658)
14. *Two planes do not enclose a solid figure.* (Borelli, 1658)
15. *The common section of two planes is a line, and if a line lies on two planes it is their common section.* (Borelli, 1658) [cf. I2]
16. *Two intersecting straight lines lie on the same plane.* (Borelli, 1658) [*Elements* XI, 2]
17. *Two intersecting straight lines may have at most one point in common.* (Arnauld, 1667) [cf. P6*]
18. *A bounded solid body can be divided into two parts in any direction. Taking together all the divisions, it will be divided into an innumerable number of parts.* (Roberval, 1675)
19. *The division of a body may be physical, thus disconnecting the parts of the body, or mathematical, thus preserving its continuity.* (Roberval, 1675)
20. *Besides the surfaces that bound a solid body, innumerable other surfaces may be conceived of which bound its parts.* (Roberval, 1675)
21. *A bounded surface can be divided into an innumerable number of lines, and a bounded line into an innumerable number of points.* (Roberval, 1675)
22. *The existence of a bounded solid body implies the existence of surfaces, the existence of bounded surfaces that of lines, the existence of bounded lines that of points.* (Roberval, 1675)
23. *However big a bounded solid body is posited, a bigger one may be conceived of that contains the former as a proper part. The included body may be conceived of as separated from the bigger one by some space.* (Roberval, 1675)
24. *A solid body revolved around two fixed points has an entire line fixed.* (Roberval, 1675) [cf. T2]
25. *If a line is perpendicular to another line, its extension is also perpendicular to it.* (Lamy, 1685)
26. *If a line is perpendicular to another line, the latter is also perpendicular to the former.* (Lamy, 1685)
27. *If a straight line perpendicular to a plane moves uniformly in a straight line on the plane, its flow will produce a plane perpendicular to the first plane.* (Lamy, 1685)
28. *If a straight line is perpendicular to a plane, a plane passing through the straight line is also perpendicular to the first plane.* (Lamy, 1685)
29. *The extremities of a line are points.* (Lamy, 1685) [*Elements* I, def. 3]
30. *A straight line is determined by two points only.* (Lamy, 1685)
31. *Only one straight line may be drawn between two points.* (Lamy, 1685) [cf. I17]
32. *Two straight lines that have two points in common coincide.* (Lamy, 1685) [cf. I17]
33. *A straight line cannot be partly on one plane and partly on another plane.* (Lamy, 1685) [*Elements* XI, 1]
34. *Every triangle lies on a plane.* (Lamy, 1685) [*Elements* XI, 2]
35. *If a part of a plane is congruent to a part of another plane, the two planes are congruent to one another.* (Marchetti, 1709) [cf. I13]
36. *A straight line* [i.e., a segment] *may only be bisected in one point.* (Rondelli, 1719)
37. *To draw a straight line perpendicular to any given straight line in any given point.* (Simpson, 1747) [*Elements* I, 11 and 12].
38. *An infinite straight line divides the infinite plane in two parts*. (Kästner, 1758).
39. *It is possible to extend a plane through two intersecting lines.* (Simpson, 1760)

### Principles of continuity

1. *If a straight or curved line is drawn from a point which is within a figure to another point in the same plane which is outside the figure, it will intersect the sides or boundary of the figure.* (Fine, 1532)
2. *If a straight line drawn from any angle of a rectilinear figure meets the opposite side or angle of the figure, it also intersects this latter side or angle.* (Fine, 1532)
3. *If a circumference with a given center and a radius of a given length is produced, and the radius is extended beyond the center to infinity, it will intersect the circumference.* (Richard, 1645)
4. *Only two points on a circumference are on one straight line. That is: if a straight line intersects a circle entering in its area and leaving it, it will intersect the circle at two points only, the one when it enters it and the other when it leaves it.* (Richard, 1645)
5. *If a circumference is described which has an end of a given segment as its center and passes through the other end of the segment, it will also pass through the ends of all segments equal to the given one that are produced from the given center.* (Richard, 1645)
6. *If a circumference is described which has its center on the side or boundary of a given figure, and whose radius falls inside the figure (not on its boundary), it will intersect the figure at least once.* (Richard, 1645)
7. *If a circumference is described which has its center outside a given figure, and whose radius falls inside the figure (not on its boundary), it will intersect the figure at least once.* (Richard, 1645)
8. *If a straight line drawn inside a figure is produced to infinity (if needed) in both directions, it will intersect the boundary of the figure.* (Richard, 1645)
9. *If a straight line divides a rectilinear angle and is produced to infinity, it will intersect a given straight line which is applied* [*with its ends*] *to the straight lines forming the angle. And a straight line drawn from a rectilinear angle to a point inside its base will divide the angle. And a straight line drawn from a point of the base of a triangle to the opposite angle will divide the angle.* (Richard, 1645)
10. *If a point is taken inside a space which is everywhere bounded by lines, and an infinite straight line passes through the point, it will cut the boundary of the space at at least two points.* (Pascal, 1655)
11. *If two points are taken on the different sides of a straight line, a straight line going from one point to the other will intersect the first line at exactly one point.* (Pascal, 1655)
12. *An infinite straight line passing through a point inside a circle intersects the circumference at exactly two points.* (Pascal, 1655)
13. A *circumference passing through a point inside a circle and a point outside it intersects the circumference of the circle at exactly two points.* (Pascal, 1655)
14. *If two circumferences both have some points inside one another, they will intersect one another at exactly two points.* (Pascal, 1655)
15. *If a circumference has a point on one side of an infinite straight line and the center on the other side, it intersects the straight line at exactly two points.* (Pascal, 1655)
16. *If the same straight line is inside two figures, the two figures will have a common part and will intersect one another.* (Borelli, 1658)
17. *If two* *equal* *circles have their centers on the ends of a segment taken as their radius, they meet.* (Schott, 1661)
18. *If a point F is taken in the base BD of a triangle BCD and a straight line FG is drawn inside the triangle and extended enough, it will intersect one of the other sides BC or CD at a point G, or both of them at their common point C (and C is then the same point as G).* (Roberval, 1675)
19. *If an infinite straight line passes inside a plane or a solid figure, it will intersect the boundaries of this figure.* (Roberval, 1675)
20. *If a segment has one end inside a circle and one end outside it, it intersects the circumference at one point.* (Kästner, 1758)

### Principles of congruence, motion, and transportation

1. *Quantities and their properties may be superposed by an intellectual act.* (Richard, 1645)
2. *Quantities may be conceived to move completely or around something fixed.* (Richard, 1645)
3. *Quantities the divisible boundaries of which are congruent are themselves congruent and equal to one another. Equal straight lines are congruent with one another. Equal rectilinear angles are congruent with one another. Equal circles are congruent with one another. [Equal] arcs of equal circles are congruent with one another. Equilateral and equiangular rectilinear figures are congruent with one another, and equal.* (Richard, 1645)
4. *If the ends of two straight lines are congruent with one another, the straight lines are themselves congruent and equal.* (Richard, 1645)
5. *The mere change of position of a triangle or any other plane and rectilinear figure does not change anything in its sides or angles.* (Richard, 1645)
6. *Given a thing, to reproduce it.* (Ricci, 1651)
7. *Given two things, to superpose them.* (Ricci, 1651)
8. *If the boundaries of two plane figures are congruent, the two figures are themselves congruent.* (Ricci, 1651)
9. *If two plane figures are congruent, their boundaries are congruent as well.* (Ricci, 1651)
10. *Equal straight lines are congruent with one another.* (Tacquet, 1654) [cf. T3]
11. *Equal circles are congruent with one another.* (Tacquet, 1654) [cf. T3]
12. *Equal angles are congruent with one another*. (Barrow, 1655) [cf. T3, and E4]
13. *To draw a straight line equal to a given straight line*. (Arnauld, 1667) [*Elements* I, 2]
14. *Things with equal motions traverse equal spaces in equal times.* (Fabri, 1669)
15. *To move the compass keeping its opening.* (Fabri, 1669)
16. *To bend a straight line through the movement of the sides.* (Fabri, 1669)
17. *If a rectilinear angle is congruent with another rectilinear angle and it is reflected without changing its magnitude or width, it will be still congruent with the second angle.* (Roberval, 1675)
18. *To put two straight lines directly one to the other.* (Scarburgh, 1705)
19. *Any extended entity you please may move with any given law whatever.* (Newton, 1693–1706)
20. *A moving body may halt in any given position whatever.* (Newton, 1693–1706)
21. *Extended entities may describe the paths marked out through the courses of moving things or by the position of stationary ones.* (Newton, 1693–1706)
22. *To draw through two given points a line congruent to a given line at a distance no less than that of the points, an ordained point of which shall coincide with one or other given point.* (Newton, 1693–1706)
23. *To draw any line on which there shall always fall a point which is given according to a precise rule by drawing from points through points lines congruent to given ones.* (Newton, 1693–1706)
24. *To extend from any given point a straight line equal to a given straight line.* (Marchetti, 1709) [*Elements* I, 2; cf. T13]
25. *To cut from any given straight line a portion equal to a shorter given straight line.* (Marchetti, 1709) [*Elements* I, 3]
26. *To reproduce any straight line or rectilinear angle.* (Simpson, 1747) [*Elements* I, 2–3 and *Elements* I, 23].
27. *The magnitudes that are congruent with one another are similar and equal, and the magnitudes that are similar and equal are congruent with one another.* (König, 1758)

### Principles of the equality and the comparison of measure

1. *The shortest line between two points is the straight line*. (Bradwardine, fourteenth century) [Archim. *De sphaera et cylindro* I, Ass. 1]
2. *If two more sides are drawn on the base of a triangle, they will be greater or lesser than the previous sides.* (Vögelin, 1528) [*Elements* I, 7]
3. *If two triangles have the two sides equal to two sides respectively, and have the angles contained by the equal sides equal, they will also have the base equal to the base, the triangle will be equal to the triangle, and the remaining angles will be equal to the remaining angles respectively.* (Pelletier, 1557) [*Elements* I, 4]
4. *If one of several equal angles is a right angle, all angles will be right angles.* (Herigone, 1634) [cf. Proclus, *In Euclidis* 189]
5. *The distance between two points is the straight line.* (Pascal, 1655) [cf. Archim. *De sphaera et cylindro* I, Ass. 1; cf. E1]
6. *The diameter bisects the circle.* (Pascal, 1655) [*Elements* I, def. 18].
7. *All lines drawn from the center to the circumference of a circle are equal to one another.* (Borelli, 1658)
8. *Two circles that have equal radii are equal.* (Borelli, 1658)
9. *Two equal circles have equal radii.* (Borelli, 1658)
10. *If, from the two ends of a straight line, there is drawn an arc of a circle, as well as two straight lines meeting at a point outside of the circle and not intersecting the circle, and a curve entirely outside the circle, the latter curve and the two straight lines will both be longer than the circle, and the circle will be longer that the initial straight line.* (Borelli, 1658)
11. *If two lines on the same plane have their ends in common and are both convex toward the same part, the one which is contained by the other is shorter than it.* (Arnauld, 1667) [Archim. *De sphaera et cylindro*, Ass. 2]
12. *The degrees of equal circumferences are equal*. (Arnauld, 1667)
13. *Among all straight lines drawn from the center of a circle, those smaller than the radius have their other end inside the circle, those equal to the radius have their other end on the circumference, and those longer than the radius have their other end outside of the circle*. (Arnauld, 1667)
14. *If two straight lines intersect, and two points of the cutting line are singularly equidistant from two points of the other line, then all other points in the cutting line will also be equidistant from those two points of the other line.* (Arnauld, 1667)
15. *Squares with equal sides are equal to one another.* (Arnauld, 1667)
16. *Rectangles with equal bases and heights are equal to one another.* (Arnauld, 1667)
17. *Similar segments of circles on equal straight lines are equal to one another.* (Arnauld, 1667) [*Elements* III, 24].
18. *A line between two points that deviates toward one part of the other from the straight line between the same points is longer than the straight line.* (Lamy, 1685)
19. *A polygon is larger than the circle inscribed in it.* (Lamy, 1685)
20. *A polygon is smaller than the circle in which it is inscribed.* (Lamy, 1685)
21. *If one end of a straight line lies on a plane and is conceived as the center of a circle in the plane, the straight line is perpendicular to the plane if and only if the other end is equidistant from the circumference of the circle.* (Lamy, 1685)
22. *A solid figure is bigger than the one to which it is circumscribed and smaller than the one to which it is inscribed.* (Lamy, 1685)
23. *Among prisms with equal height, the one with the smaller base is smaller.* (Lamy, 1685)
24. *In equal circles, arcs of equal chords are equal and chords of equal arcs are equal.* (Lamy, 1685) [*Elements* III, 23 and 24; cf. E17]
25. *The space ABC formed by the straight lines running from a point A to all the points of a line BC is the smallest space bounded by AB, BC, and AC.* (Prestet, 1689)
26. *Equal circles have equal circumferences.* (Marchetti, 1709)
27. *If two circles have equal circumferences, they are equal.* (Marchetti, 1709)
28. *A greater circle has a greater circumference and greater radii.* (Marchetti, 1709)
29. *If a circle has a greater circumference or radius than another circle, it is greater than the latter.* (Marchetti, 1709)
30. *In a triangle two sides taken together in any manner are greater than the remaining one.* (Marchetti, 1709) [*Elements* I, 20]
31. *If two straight lines forming an angle have the same length as two other straight lines forming an equal angle, the segment joining the ends of the former lines will be equal to the segment joining the ends of the latter lines.* (Simpson, 1747) [*Elements* I, 4; cf. E3].
32. *If two solids of the same height are cut by planes parallel to their bases and the sections at equal distance from the bases are either equal or in constant ratio, the solids themselves are equal or in the same constant ratio.* (Simpson, 1747)
33. *Arcs of a circle that contain equal angles are similar to one another.* (König, 1758)
34. *Upright prisms with equal bases and heights are equal to one another.* (Simpson, 1760) [cf. *Elements* XI, 29]
35. *The circumference of a circle is greater than the perimeter of any inscribed polygon, and lesser than the perimeter of any circumscribed polygon.* (Simpson, 1760) [cf. E19 and E20]

### The parallel postulate

1. *If two straight lines lying on the same plane and extended to infinity in both directions do not meet, they are equidistant.* (Fine, 1532)
2. *If a straight line meets one parallel, it will also meet the other parallels.* (Ricci, 1651)
3. *Parallel lines have a common perpendicular.* (Tacquet, 1654)
4. *Two perpendiculars cut off equal segments from each of two parallel lines.* (Tacquet, 1654)
5. *If a straight line perpendicular to a given straight line is translated into a plane remaining with one of its ends on the given straight line, the other end of it will draw with its flow another straight line.* (Borelli, 1658)
6. *If two straight lines extended toward one side come closer and closer, they will eventually meet.* (Arnauld, 1667)
7. *If two lines are parallel, all the perpendiculars from one to the other are equal to one another.* (Dechales, 1672)
8. *If two straight lines diverge more and more when they are extended in one direction, they will converge and meet in the other direction.* (Roberval, 1675)
9. *Two straight lines do not incline toward one another if one of them does not incline toward a third, unless the other also inclines toward it.* (Mercator, 1678)
10. *Two non-equidistant straight lines lying on the same plane, if produced far enough, will meet on one side.* (Rondelli, 1719) [cf. PP6]
11. *If two points on a straight line have different distances to another straight line, the two straight lines, infinitely produced, will meet.* (Simpson, 1747) [cf. PP10]
12. *A straight line passing through a point that is inside an angle cuts at least one of the sides of the angle.* (Lorenz, 1773)
13. *Two straight lines cannot be drawn through the same point, parallel to the same straight line, without coinciding with one another.* (Playfair, 1795)
14. *If two straight lines forming an angle are infinitely produced from a point, their distance will exceed any finite magnitude.* (Commandino, 1572)
15. *If one assumes that two straight lines which form internal angles equal to two right angles when intersected by a third straight line meet in that direction, one should also grant that the two lines always meet when they form the same angles with a third line.* (Richard, 1645)

### Hilbert’s Axioms of geometry

#### Axioms of incidence

1. *For every two points A, B there exists* *a line* a *that contains each of the points A, B.*
2. *For every two points A, B there exists no more than one line that contains each of the points A, B.*
3. *There exist at least two points on a line. There exist at least three points that do not lie on a line.*
4. *For any three points A, B,* C *that do not lie on the same line there exists a plane* $\alpha$ *that contains each of the points A, B,* C. *For every plane there exists a point which it contains.*
5. *For any three points A, B,* C *that do not lie on one and the same line there exists no more than one plane that contains each of the three points A, B,* C.
6. *If two points A, B of a line* a *lie in a plane* $\alpha$ *then every point of* a *lies in the plane* $\alpha$.
7. *If two planes* $\alpha , \beta$ *have a point A in common then they have at least one more point B in common.*
8. *There exist at least four points which do not lie in a plane.*

#### Axioms of order (or Betweenness)

1. *If a point B lies between a point A and a point* C *then the points A, B,* C *are three distinct points of a line, and B then also lies between* C *and A.*
2. *For two points A and* C, *there always exists at least one point B on the line AC such that* C *lies between A and B.*
3. *Of any three points on a line there exists no more than one that lies between the other two.*
4. *Let A, B, C be three points that do not lie on a line and let* a *be a line in the plane ABC which does not meet any of the points A, B, C. If the line* a *passes through a point of the segment AB, it also passes through a point of the segment AC, or through a point of the segment BC.* (Pasch’s Axiom)

#### Axioms of congruence

1. *If A, B are two points on a line* a*, and A* $^{\prime }$ *is a point on the same or another line* a$^{\prime }$ *, then it is always possible to find a point B* $^{\prime }$ *on a given side of the line* a$^{\prime }$ *through A* $^{\prime }$ *such that the segment AB is congruent or equal to the segment* $A^{\prime }B^{\prime }$. *In symbols* $AB \cong A'B'$.
2. *If a segment A* $'B'$ *and a segment* $A''B''$, *are congruent to the same segment AB, then the segment A* $'B'$ is *also congruent to the segment* $A''B''$, *or briefly, if two segments are congruent to a third one they are congruent to each other.*
3. *On the line a let AB and BC be two segments which except for B have no point in common. Furthermore, on the same or on another line* a$'$ *let A* $'B'$ *and B* $'C'$ *be two segments which except for B* $'$ *also have no point in common. In that case, if* $AB \cong A^{\prime }B^{\prime }$ *and* $BC \cong B^{\prime }C^{\prime }$, *then* $AC \cong A^{\prime }C^{\prime }$.
4. *Let* $\angle$ *(h,k) be an angle in a plane* $\alpha$ *and* a$'$ *a line in a plane* $\alpha '$ *and let a definite side of* a$'$ *in* $\alpha '$ *be given. Let h* $'$ *be a ray on the line* a$'$ *that emanates from the point O* $'$. *Then there exists in the plane* $\alpha '$ *one and only one ray k* $'$ *such that the angle* $\angle$ *(h,k) is congruent or equal to the angle* $\angle$ *(h* $'$, $k'$ *) and at the same time all interior points of the angle* $\angle$ *(h* $'$, $k'$ *) lie on the given side of* a$'$. *Symbolically* $\angle$ *(h,k)* $\cong \angle$ *(h* $'$, *k*’*). Every angle is congruent to itself, i.e.,* $\angle$ *(h,k)* $\cong \angle$ *(h,k) is always true.*

#### Axiom of parallels

1. *Let* a *be any line and A a point not on it. Then there is at most one line in the plane, determined by* a *and A, that passes through A and does not intersect* a. (Playfair’s Axiom)

#### Axioms of continuity

1. *If AB and CD are any segments then there exists a number n such that n segments CD constructed contiguously from A, along the ray from A through B, will pass beyond the point B.* (Axiom of Archimedes)
2. *An extension of a set of points on a line with its order and congruence relations that would preserve the relations existing among the original elements as well as the fundamental properties of line order and congruence that follow from Axioms I–III and from V-1 is impossible.* (Axiom of Linear Completeness)

## The editions of the *Elements*

### Greek manuscripts of the *Elements* used by Heiberg

- **P** (Vatican, Early ninth century; partially belonging to a non-Theonine tradition)
: P1, P2, P3, P4, P5, P6*.
: CN1, CN2, CN3, CN6*, CN7*, CN8*, CN9*, CN4, CN5.

- **B** (Oxford, 888 AD)
: P1, P2, P3, P4, P5.
: CN1, CN2, CN3, CN6*, CN8*, CN9*, CN4, CN5, P6*.

- **F** (Firenze, tenth century)
: P1, P2, P3, P4, P5, P6*.
: CN1, CN2, CN3, CN6*, CN8*, CN9*, CN4, CN5.

- ***b*** (Bologna, eleventh century)
: P1, P2, P3, P4, P5.
: CN1, CN2, CN3, CN6*, CN8*, CN9*, CN4, CN5, P6*.

- **V** (Wien, twelfth century)
: P1, P2, P3, P4, P5, [P6*].
: CN1, CN2, CN3, CN6*, CN7*, CN8*, CN9*, CN4, CN5, P6*.

- ***p*** (Paris, twelfth century)
: P1, P2, P3, P4, P5.
: CN1, CN2, CN3, CN6*, CN8*, CN9*, CN4, CN5, P6*.

- ***q*** (Paris, twelfth century)
: P1, P2, P3, P4, P5, P6*.
: CN1, CN2, CN3, CN6*, CN7*, CN8*, CN4, CN5.

### Medieval translations in Latin manuscripts

- [Boethius], *Geometria*.

- Book I
- *Petitiones, sive Postulata*: P1, P2, P3, P4, P5.
- *Communes animi conceptiones*: CN1, CN3, CN2, CN4.

$\bullet$ Cassiodorus (*Institutiones* II, vi 3) states that Severinus Boethius (early sixth century) translated Euclid into Latin. A few fragments of this translation were preserved in the Middle Ages and merged with other geometrical material (mostly for practical use). However defective, “Boethius’s” geometry remained for several centuries the only access to Euclid’s *Elements* in the West. Although a complete critical edition of the Boethian fragments is still missing, all the present evidence agrees upon the above system of principles. Boethius’s *Geometria altera* (the most important item attributed to him on this topic) was printed in Venice in 1492, and later appended to the edition of the Sacrobosco’s *Sphaera* published in Paris in 1500 (with several reprints, ed. Lefèvre d’Étaples), but we will not follow this trend any further. A modern critical edition of the *Geometria altera* is to be found in Folkerts (1970).

- Adelard of Bath, first half of twelfth century.

- Book I
- *Petitiones quinque*: P1–P2, P3, P4, P5, P6*.
- *Scientia universaliter communis*: CN1, CN2, CN3, CN6*, A1, CN9*, CN4, CN5.

$\bullet$ Translated from a modified version of the Arabic translation of Al-Hajjāj (first half of the ninth century). It is the earliest still-extant translation of Euclid into Latin. Axiom A1 is missing in some manuscripts. Edition in Busard (1983).

- Hermann von Carinthia, first half of twelfth century.

- Book I
- *Quinque modi investigandi*: P1–P2, P3, P4, P5, P6*.
- *Sapientia*: CN1, CN2, CN3, CN6*, CN9*, A1, CN4, CN5.

$\bullet$ Translated from a modified version of the Arabic translation of Al-Hajjāj, but probably independent from Adelard’s tradition. Book I only survives in one manuscript and it may have been modified since Hermann’s translation. A discussion on Hermann’s sources is to be found in Brentjes (2001b). Edition in Busard (1967, 1977).

- Robert of Chester, first half of twelfth century.

- Book I
- *Peticiones sunt quinque*: P1–P2, P3, P4, P5, P6*.
- *Communes animi concepciones*: CN1, CN2, CN3, CN6*, A1, CN9*, CN4, CN5.

$\bullet$ Formerly attributed to Adelard, the authorship of Robert of Chester is still doubtful, but the text seems to be posterior to Adelard’s version. It also depends on Hermann von Carinthia’s version. Some manuscripts only have the statements of the propositions without the demonstrations, while others have hints on how carry on the proofs, and still others show the complete text. It was one of the most widespread editions of Euclid in the Middle Ages. The system of principles follows Adelard’s version. A few manuscripts add the axiom CN8* before CN9*. Edition in Busard and Folkerts (1992).

- Gerardo da Cremona, *Liber Euclidis philosophi*, mid. twelfth century.

- Book I
- *Ea in quibus necesse est convenire*: P1, P2, P3, P4, P5.
- *Communes animi conceptiones*: CN1, CN2, CN3, CN6*, CN7*, CN8*, CN9*, CN4, CN5, P6*.

$\bullet$ An important edition of the *Elements* translated from the Arabic, which follows a modified version of the Arabic translation made by Isḥāq ibn Ḥunain and revised by Thābit ibn Qurra in the late ninth century (generally considered mathematically more reliable than the version by Al-Hajjāj). Edition in Busard (1984). A comprehensive study of Gerardo’s version is to be found in de Young (2004).

- Gerardo da Cremona, [*Anaritii in decem libros priores elementorum Euclidis commentarii*], mid. twelfth century.

- Book I
- *Petitiones*: P1, P2, P3, P4, P5, [P6*].
- *Propositiones per se note*: CN1, CN2, CN3, CN6*, CN7*, CN8*, CN4, CN5, P6*, M1, M2, I1, I2, I3, P2–I4.

$\bullet$ A translation from the Arabic of the commentary to the *Elements* written by An-Nayrīzī (tenth century), who was in turn reading Simplicius’s and Heron’s lost Greek commentaries to Euclid. An-Nayrīzī reports that Simplicius considered P6* to be an interpolation; An-Nayrīzī also gives a proof of it. The original Arabic version also has CN9* after CN8*. Edition in Curtze (1899), improved in Tummers (1994) for Books I–IV; English translation and commentary in Lo Bello (2003a, b). On the relation between the Arabic original by An-Nayrīzī and Gerardo’s translation, see Brentjes (2001a).

- Anonymous, second half of twelfth century.

- Book I
- Unlabeled principles: P1, P2, P3, P4, P5; CN1, CN2, CN3, CN6*, CN8*, CN9*, CN4, CN5, P6*.

$\bullet$ Possibly the first medieval translation made from the Greek. The final editor is not known, but probably the translation is the work of several scholars working at the court first of Frederick II and then of his son Manfredi in Sicily. Most of the text seems to follow a Greek manuscript close to Heiberg’s manuscript **B**, with several discrepancies. The system of principles seems to follow manuscripts **V** or **p**. Critical edition in Busard (1987).

- John of Tynemouth, late twelfth century or beginning of the thirteenth century.

- Book I
- *Petitiones sunt quinque*: P1–P2, P3, P4, P5, P6*.
- *Communes animi conceptiones*: CN1, CN2, CN3, CN6*, A1, CN9*, CN4, CN5, A3, R1. (...)

- Book VII
- *Communes animi conceptiones*: N1, N2, N3. (...)

$\bullet$ Translation from the Arabic probably made by John of Tynemouth (Johannes de Tinemue), formerly attributed to Adelard and following the same tradition of the Arabic text. Tynemouth made use of Adelard’s system of principles. However, he claimed that the common notions are infinite in number, and gave axioms A3 and R1 in Book I as examples of them. Tynemouth also stated a few arithmetical common notions in Book VII, which form the first basis for an axiomatization of arithmetic in Euclid’s *Elements*. They were to be found in An-Nayrīzī’s commentary to the *Elements* (see Curtze 1899, p. 191), who in turn attributed them to the commentary of Heron after *Elements* VII, 2 (without explicitly saying whether these assumptions, needed to prove *Elements* VII, 3, were to be considered as axioms). Gerardo da Cremona’s translation of An-Nayrīzī mentioned this point, but did not include the latter propositions as axioms of Book VII. The statement about the infinite number of common notions is also to be found in other anonymous manuscripts in the tradition of Robert of Chester. These thirteenth-century manuscripts (that may predate, or not, Tynemouth’s version) also added axioms A3 and R1 to Book I, but had no principles in Book VII; they have been edited in Busard (1996). The dating of Tynemouth’s version is controversial; for an educated guess that it is to be dated to the thirteenth century, see Knorr (1990). The edition of Tynemouth’s text is in Busard (2001).

- Jordanus de Nemore, *De elementis arithmetice artis*, first half of the thirteenth century.

- *Petitiones sunt tres*: N4, N5, N6.
- *Communes animi conceptiones sunt octo*: A2, N7, M3, M4, N8, N9, N10, N11.

$\bullet$ Jordanus’s *Arithmetica* was the most important medieval treatise on arithmetic, but it is not an edition of the *Elements*. The material treated in it comes from the arithmetical Books of the *Elements* (in the version of Gerardo), as well as from Boethius’s *De institutione arithmetica* (itself a translation of a Neopythagorean tract by Nichomacus of Gerasa) and several Arabic sources. Jordanus’s axiomatics, however, seems to be his own creation (as it is not attested to in his known sources), and it influenced the further tradition of the arithmetical Books of the *Elements* (starting with Campano’s edition). While Regiomontanus and Maurolico tried in vain to publish Jordanus’s *Arithmetica*, it eventually went to print in 1496, edited (and modified) by Lefèvre d’Étaples (see below). Edition in Busard (1991).

- Campano da Novara, around 1255–1259.

- Book I
- *Petitiones sunt quinque*: P1–P2, P3, P4, P5, P6*.
- *Communes animi conceptiones*: CN1, CN2, CN3, CN6*, A1, CN9*, CN4, CN5; A3, R1. (...)

- Book VII
- *Petitiones*: N12, N5, N6, N13.
- *Communes animi conceptiones*: A2, M3, M4, N8, N7, N9, N10, N2, N3, N1.

$\bullet$ This edition of Euclid is not, properly speaking, a new translation but rather a recension of various earlier Latin editions. The main text used in this version seems to be one in the tradition of Robert of Chester, but Campano emended and integrated it, drawing on An-Nayrīzī (via Gerardo da Cremona), John of Tynemouth, and the above-mentioned Latin translation made from the Greek text. Most of the arithmetical principles in Book VII come from Jordanus de Nemore’s *Arithmetica*, while the others are drawn from Tynemouth’s edition. Campano adds that R1 (the existence of the fourth proportional) is universally valid in continuous quantities, but it may be applied to numbers only when the second quantity is a multiple of the first, since numbers cannot be diminished to infinity (cf. N13). This N13 in Book VII is probably an original addition by Campano, and its main aim seems to be to allow a stop in the decomposition of a number into its factors (cf. *Elements* VII, 31); on Campano’s system of arithmetical principles, see Rommevaux (1999). Printed in 1482 (see below). Edition in Busard (2005).

- Thomas Bradwardine, *Geometria speculativa*, around 1320–1335.

- Book I
- *Petitiones ab Euclyde ponuntur quinque*: P1–E1, P3, P4, P5, P6*.
- *Communes scientie multe sunt, sed sufficiant novem*: A4, CN5, CN1, A5, CN2, CN3, CN6*, CN7*, CN4. (...)

$\bullet$ Bradwardine’s treatise is a commentary on a few propositions of Euclid’s *Elements* and several other mathematical results taken from various sources (including Archimedes). Bradwardine’s Latin text generally follows Campano as far as the *Elements* are concerned, but also modified it under several respects. In particular, Bradwardine employed Archimedes’s famous axiom (from *De sphaera et cylindro*), stating that the straight line is the shortest line, to ground the geometry of the *Elements*; and possibly rejected postulate P2 on the extendibility of a straight line on metaphysical grounds, i.e., since the universe is finite (he offered a discussion on the topic in his major theological treatise, *De causa dei*). Bradwardine also introduced principle A4 that will be widely employed in further axiomatizations and represented the grounding principle of many early modern (extensional) mereological theories. He also added principle A7, which seems to be false as it is spelled and received no further attention in subsequent treatments of the matter. It should be noted that, in the fourth part of the book, Bradwardine attempted to prove a few assumptions among those mentioned by An-Nayrīzī (in Gerardo da Cremona’s translation), such as I1, I2 and I3. Bradwardine had probably written an *Arithmetica speculativa*, even though scholars are still in doubt about the exact version of it that should be ascribed to him (several works of this kind have been attributed to Bradwardine in the past); none of these works, however, contains axioms for the theory of numbers. The *geometria speculativa* was printed in 1495 (see below), and can now be read in Molland (1989). See also the essays by Molland (1978) (on Bradwardine’s *geometria*) and Busard (1971, 1998) (on Bradwardine’s and Johannes de Mures’s *arithmetica*).

### Printed editions

- Campano da Novara, *Preclarissimus liber elementorum Euclidis perspicacissimi, in artem geometrie incipit quamfoelicissime*, Venezia, Ratdolt, 1482.

- Book I
- *Petitiones sunt quinque*: P1–P2, P3, P4, P5, P6*.
- *Communes animi conceptiones*: CN1, CN2, CN3, CN7*, CN6*, A1, CN9*, CN4, CN5, A3, R1. (...)

- Book VII
- *Petitiones*: N12, N5, N6, N13.
- *Communes animi conceptiones*: A2, M3, M4, N8, N7, N9, N10, N2, N3, N1.

$\bullet$ A printed edition of the medieval translation, and the first of all scientific treatises to go into print. The editor added CN7* among the common notions, which is absent in the known manuscript tradition.

- Thomas Bradwardine, *Breve compendium artis geometrie*, ed. Pedro Sánchez Ciruelo, Paris, Guy Marchant, 1495.

- Book I
- *Petitiones ab Euclyde ponuntur quinque*: P1–E1, P3, P4, P5, P6*.
- *Communes scientie multe sunt, sed sufficiant novem*: A4, CN5, CN1, A5, CN2, CN3, CN6*, CN7*, CN4. (...)

$\bullet$ A printed edition of Bradwardine’s *geometria speculativa*, edited by the mathematician and theologian Ciruelo. The volume was reprinted several times in the following decades and exerted a certain influence on many authors, such as Pacioli, Bovelles, Vögelin and others.

- Jacques Lefèvre d’Étaples, *Arithmetica decem libris demonstrata*, Paris, Higman, 1496.

- *Dignitates*: A2, N7, M3, M4, N8, N9, N10, N11, N14, CN1, CN3, M7, M8, CN2, M6, A4, N15, N16, R2, M5.
- *Petitiones*: N4, N5, N6, N13, N17, N18.

$\bullet$ A printed edition of Jordanus’s *Arithmetica*. Lefèvre d’Étaples (Faber Stapulensis), however, added several arithmetical axioms and postulates, which are partly new and partly taken from the medieval Euclidean tradition. The treatise had a big influence on the cossist tradition, and for instance the celebrated *Arithmetica integra* (1544) by Michael Stifel (1487–1567) depended heavily on Lefèvre’s version of Jordanus. We will not follow further this line of development, which falls outside of the tradition proper of the *Elements*.

- Giorgio Valla, *De expetendis et fugiendis rerum opus*, Venezia, Manuzio, 1501.

- Book I
- *Petitiones*: P1, P2, P3, [P4], P5.
- *Axiomata*: CN1, CN2, CN3, CN6*, CN7*, CN8*, CN9*, CN4, CN5, P6*.

$\bullet$ Several books of Valla’s posthumous encyclopedic work deal with the content of the *Elements*, which Valla could read in the Greek and of which he translated about a hundred of propositions. He mentioned no principles for arithmetic (since he found none in the Greek manuscripts), but provided a list of principles for geometry (Book X, Chapter 110). Integrating the Greek manuscript of the *Elements* with Proclus’s commentary, he stated that P4 can in fact be proven. Note that even if their sources were different, Valla’s system of principles is the same as Gerardo da Cremona’s (but different from Campano’s).

- Bartolomeo Zamberti, *Euclidis megarensis philosophi platonici Mathematicarum disciplinarum Janitoris: Habent in hoc volumine quicumque ad mathematicam substantiam aspirant: elementorum libros XIII. cum expositione Theonis*, Venezia, Tacuino, 1505.

- Book I
- *Postulata*: P1, P2, P3, P4, P5.
- *Communes sententiae*: CN1, CN2, CN3, CN6*, CN7*, CN8*, CN9*, CN4, CN5, P6*.

$\bullet$ The first printed translation made from the Greek, and thus providing the system of principles of some Greek manuscripts. Zamberti’s edition is the heir of Valla’s endeavor (see above), and he often reproduced the latter translation. Zamberti’s system of principles is in fact the same as Valla’s. Zamberti was highly critical regarding the “barbarisms” of Campano’s translation from the Arabic and initiated a long-lasting quarrel on the superiority of the Greek manuscript tradition over the Arabic one.

- Luca Pacioli, *Euclidis megarensis philosophi acutissimi mathematicorumque omnium sine controversia principis opera a Campano interprete fidissimo translata*, Venezia, Paganino, 1509.

- Book I
- *Petitiones*: P1–P2, P3, P4, P5, P6*.
- *Communes animi conceptiones*: CN1, CN2, CN3, CN7*, CN6*, A1, CN9*, CN4, CN5, A3, R1. (...)

- Book VII
- *Petitiones*: N12, N5, N6, N13.
- *Communes animi conceptiones*: A2, M3, M4, N8, N7, N9, N10, N2, N3, N1.

$\bullet$ An emended version of Campano (1482), with Pacioli’s remarks. It was written in 1485 and possibly printed already in 1486. Pacioli may also have translated the *Elements* into Italian, but this work is now lost.

- Jacques Lefèvre d’Étaples, *Euclidis Megarensis Geometricorum elementorum libri XV. Campani Galli transalpini in eosdem commentariorum libri XV. Theonis Alexandrini Bartholomaeo Zamberto Veneto interprete, in tredecim priores, commentariorum libri XIII*, Paris, Henri Estienne, 1516.

$\bullet$ A celebrated synoptic edition of Campano and Zamberti, with a comparison of their respective systems of principles. It was reprinted several times (see for instance the edition published in Basel, Herwagen 1537).

- Johannes V$\ddot{\textsc {o}}$ gelin, *Elementale geometricum ex Euclidis geometria*, Wien, Singren, 1528.

- Book I
- *Postulata*: P4, P5, P6*, E2.
- *Communes sententiae*: CN1, CN2, CN3, CN6*, CN7*, CN8*, CN9*, CN4, CN5, A4.

$\bullet$ Vögelin’s version is not a proper edition of the *Elements* but rather a collection of a few important theorems taken from Books I, III, V and VI. It is remarkable that Vögelin did not include P1, P2 and P3 among the principles, whereas he added *Elements* I, 7 (=E2) as a postulate, since (he claimed) it is difficult to prove and his aim was to write a book for students. Reprinted in 1536 with a preface by Melanchton.

- Oronce Fine, *Protomathesis*, Paris, 1532.

- Book I
- *Postulata, seu Petitiones*: P1, P2, P3, P4, PP1, C1, C2. (...)
- *Axiomata, vel Effata, seu Communes sententiae*: CN4, CN1, M3–M4, CN2–CN3, CN6*–CN7*, P6*, CN5–A4.

$\bullet$ A treatise on several pure and applied mathematical sciences that does not follow the deductive order of the *Elements* (it begins with an essay on arithmetic). Fine allowed no axioms for the theory of numbers, but offered one of the very first reworkings of the geometrical axiomatics. Fine explicitly claimed that PP1 and C1 are needed in the proofs of the *Elements*. The Parallel Postulate (P5) is not included among the postulates (but implicitly employed many times), even though Fine stated that the latter principles are infinite in number, and the ones that he mentions at the beginning of the treatise are just a few examples. See also Fine (1536).

- Simon Grynaeus, , Basel, Herwagen, 1533.

- Book I
: P1, P2, P3.
: CN1, CN2, CN3, CN6*, CN7*, CN8*, CN9*, CN4, CN5, P4, P5, P6*.

$\bullet$ The *editio princeps* of the Greek text, and one of the most influential works in the history of mathematics. It was printed along with Proclus’s commentary to Book I. The modified division between postulates and common notions will have a great importance in the following tradition, and it is probably owed to Proclus’s opinion on the matter, who attributed it to Geminus (*In Euclidis* 181–184). Such division is already to be found in a Greek manuscripts used by Grynaeus for his edition (the *Marc. gr.* 301), dating from the fifteenth century, while the alleged source of the latter manuscript (following Heiberg’s reconstruction of this line of transmission), dating from the fourteenth century, followed the standard arrangement; it may be possible, therefore, that the modified list of axioms and postulates had its origins in the work of the fifteenth-century Byzantine scribe that produced the *Marc. gr.* 301. On the making of Grynaeus’s edition, see Oosterhoff (2014).

- Oronce Fine, *In sex priores libros geometricorum elementorum Euclidis Megarensis demonstrationes*, Paris, Simon de Colines, 1536.

- Book I
- *Postulata*: P1, P2, P3, P4, P5.
- *Communes sententiae*: CN1, CN2, CN3, CN6*, CN7*, CN8*, CN9*, CN4, CN5, P6*.

$\bullet$ Zamberti’s system of principles.

- Niccolò Tartaglia, *Euclide Megarense philosopho, solo introduttore delle scientie mathematice*, Venezia, Rossinelli, 1543.

- Book I
- *Petitioni*: P1, P2, P3, P4, P5, P6*.
- *Commune sententie*: CN1, CN2, CN3, CN6*, CN7*, CN8*, CN9*, CN4, CN5.

- Book VII
- *Petitioni*: N12, N5, N6, N13.
- *Commune conceptioni dell’animo*: A2, M3, M4, N8, N7, N9, N10, N2, N3, N1.

$\bullet$ The first printed translation of the *Elements* into Italian, although several manuscripts with previous translations are known, mostly dating from the first decades of the sixteenth century; see Pagli (2003). Tartaglia translated from Campano. He acknowledged that Campano also included A3 and R1 among the “*commune sententie*,” but claimed that this was a mistake (esp. for R1). He also removed A1 as a useless reduplication of CN1.

- Pierre de la Ramée, *Euclides*, Paris, Grandin, 1545.

- Book I
- *Postulata*: P1, P2, P3, P4, P5.
- *Communes sententiae*: CN1, CN2, CN3, CN6*, CN7*, CN8*, CN9*, CN4, CN5, P6*.

$\bullet$ Zamberti’s system of principles. It is possible that a previous edition had been published already in 1541. The volume contains no proofs of the theorems, just the statements of the propositions. Ramus was subsequently to publish, besides his celebrated *Scholae mathematicae*, also a treatise on *Arithmetica* (1557) and a *Geometria* (1669), neither of which contains any axiom. Ramus’s mathematical works enjoyed many translations in modern languages and several abridgements in the sixteenth and seventeenth Centuries. On Ramus’s edition of Euclid, see Loget (2004).

- Angelo Caiani, *I quindici libri degli Elementi di Euclide, di greco tradotti in lingua thoscana*, Roma, Blado, 1545.

- Book I
- *Domande*: P1, P2, P3.
- *Comuni pareri*: CN1, CN2, CN3, CN6*, CN7*, CN8*, CN9*, CN4, CN5, P4, P5, P6*.

$\bullet$ Grynaeus’s system of principles. Like the contemporary edition by La Ramée, Caiani only stated the theorems without proving them.

- Joachim Camerarius, *Euclidis elementorum geometricorum libri sex, conversi in Latinum sermonem*, Leipzig, Valentin, 1549.

- Book I
- *Petita*: P1, P2, P3.
- *Communes notitiae*: CN1, CN2, CN3, CN6*, CN7*, CN8*, CN9*, CN4, CN5, P4, P5, P6*.

$\bullet$ Grynaeus’s system of principles. The volume was edited by Rheticus.

- Johann Scheybl, *Euclidis Megarensis philosophi et mathematici excellentissimi, Sex libri priores de geometricis principijs*, Basel, Herwagen, 1550.

- Book I
- *Postulata*: P1, P2, P3.
- *Communes notitiae*: CN1, CN2, CN3, CN6*, CN7*, CN8*, CN9*, CN4, CN5, P4, P5, P6*.

$\bullet$ Grynaeus’s system of principles. The geometrical section of the book is introduced by a treatise on algebra.

- Robert Recorde, *The Pathway to Knowledg, containing the First Principles of Geometrie*, London, Wolfe, 1551.

- [Book II]
- *Grauntable requestes*: P1, P2, P3, P4, P5, P6*.
- *Common sentences*: CN1, CN2, CN3, CN7*, CN6*, CN8*, CN9*, CN4, CN5, A4.

$\bullet$ Recorde’s book is not an edition of Euclid, but rather a rearrangement of some propositions of the first four books of the *Elements* for practical use, and the first printed translation into English of this material. After giving the definitions and solving a few classical geometrical problems in Book I (written already in 1546), Recorde began Book II with a list of principles taken from Campano and other sources (as in the case of A4); he made no real use of these principles, though, as the *Pathway* does not contain any proof of the geometrical statements (see above the 1545 Euclidean edition by La Ramée, which may have influenced Recorde’s work). While this translation was soon superseded by the one by Billingsley and Dee (1570), it exerted a certain influence on further English editions of Euclid (especially in practical mathematics). Recorde had published his English treatise on practical arithmetic, *The Grounde of Artes*, in 1542; the latter, however, contains no principles. On Recorde’s life and works, see Roberts and Smith (2012) and Barany (2010); on the British tradition of practical mathematics, see Taylor (1954, 1966).

- Valentin Naboth, *Euclidis Megarensis mathematici clarissimi elementorum geometricorum liber primus*, Köln, Birkmann, 1556.

- Book I
- *Petita*: P1, P2, P3.
- *Communes notitiae*: CN1, CN2, CN3, CN6*, CN7*, CN8*, CN9*, CN4, CN5, P4, P5, P6*.

$\bullet$ Grynaeus’s system of principles. The volume contains a selection of theorems and problems from Book I of the *Elements*, without proofs, and a few other propositions chosen from Books II, III and VI. Naboth (aka Nabod, or Nabodus) was a famous astrologer.

- Jacques Peletier du Mans, *In Euclidis Elementa Geometrica Demonstrationum Libri sex*, Lyon, Tornes, 1557.

- Book I
- *Postulata*: P1, P2, P3, P4, [P5].
- *Animi notiones*: CN1, CN2, CN3, CN6*, CN7*, CN8*, CN9*, CN4, CN5, [P6*]; E3. (...)

$\bullet$ Zamberti’s system of principles as a starting point (but the main text is based on Campano). Peletier claimed that P5 is in fact a kind of definition of non-parallel (converging) lines and should not be placed among the postulates. He also stated that P6* should be ranged among the *postulata* rather than the *animi notiones*. Finally, he famously claimed that Euclid’s proof of *Elements* I, 4 (=E3) should be rejected, as it employs motion and superposition, and proposed to count the statement among the innumerable implicit principles of geometry.

- [Jean Magnien, Pierre de Montdoré, St. Gracilis], *Euclidis Elementorum libri XV, Graecè et Latinè*, Paris, Cavellat, 1557.

- Book I
- *Postulata*: P1, P2, P3.
- *Communes notiones*: CN1, CN2, CN3, CN6*, CN7*, CN8*, CN9*, CN4, CN5, P4, P5, P6*.

$\bullet$ Grynaeus’s system of principles. A preface by a certain St. Gracilis explains that this edition was first conceived and prepared by Jean Magnien, and later completed, after the latter’s untimely death, by Gracilis himself. Book X of the *Elements* follows the edition of it given by Pierre de Montdoré (*Euclidis elementorum liber decimus, Petro Montaureo interprete*, Paris, Vascosan 1551). The edition enjoyed several reprints and was especially used in Jesuit colleges. Like La Ramée (1545) and Caiani (1545), it gave no proofs of the propositions.

- Xylander (Wilhelm Holtzmann), *Die Sechs ersten Bücher Euclidis, vom Anfang oder Grund der Geometrj*, Basel, Kündig, 1562.

- Book I
- *Petitiones, seu Postulata*: P1, P2, P3.
- *Communes notitiae*: CN1, CN2, CN3, CN6*, CN7*, CN8*, CN9*, CN4, CN5, P4, P5, P6*.

$\bullet$ Grynaeus’s system of principles. The first printed translation of the *Elements* into German. Before Xylander, at least one other important mathematical volume had been published in German, *Das erst Buch der Geometria*, written by Wolfgang Schmidt and printed in Nürnberg in 1539. The latter book, however, did not follow the Euclidean order of the propositions, and only dealt with a few geometrical problems (rather than theorems); it included no axioms whatsoever.

- Conrad Dasypodius, *Euclidis quindecim Elementorum Geometriae primum*, Strasbourg, Mylius, 1564.

- Book I
- *Postulata*: P1, P2, P3.
- *Communes notiones*: CN1, CN2, CN3, CN6*, CN7*, CN8*, CN9*, CN4, CN5, P4, P5, P6*.

$\bullet$ Grynaeus’s system of principles. Dasypodius (Konrad Rauchfuss) edited several books of the *Elements*, in both Greek and Latin, between 1564 and 1579, without altering the list of principles. See also the important Herlin & Dasypodius (1566).

- Pierre Forcadel, *Les six premier livres des Elements d’Euclide traduicts et commentez par Pierre Forcadel de Bezies*, Paris, Marnef, 1564.

- Book I
- *Demandes*: P1, P2, P3.
- *Communes sentences*: CN1, CN2, CN3, CN6*, CN7*, CN8*, CN9*, CN4, CN5, P4, P5, P6*.

$\bullet$ Grynaeus’s system of principles. The first printed translation of the *Elements* into French. The following year, Forcadel also published his edition of the arithmetical books of the *Elements*. The latter edition has no axioms, but in discussing the proof of *Elements* VII, 1, Forcadel claimed the necessity to assume N17, N2, N1.

- Christian Herlin, Conrad Dasypodius, *Analyseis geometricae sex librorum Euclidis*, Strasbourg, Rihel, 1566.

- Book I
- *Postulata*: P1, P2, P3.
- *Communes notiones*: CN1, CN2, CN3, CN6*, CN7*, CN8*, CN9*, CN4, CN5, [P4], [P5], P6*.

$\bullet$ Grynaeus’s system of principles. A famous and influential edition that reshaped into syllogisms the proofs of Euclid. Books I and V were written by Herlin, while his pupil Dasypodius, who had already published several partial editions of the *Elements*, completed the volume. In the appended *Commentaria*, Dasypodius stated that P4 and P5 are provable and should not be counted among the *communes notiones*.

- François de Foix-Candale, *Euclidis Megarensis mathematici clarissimi elementa geometrica libri XV ad germanam geometriae intelligentiam è diversis lapsibus temporis iniuria contractis restituta*, Paris, Royer, 1566.

- Book I
- *Postulata*: P1, P2, P3.
- *Communes sententiae*: CN1, CN2, CN3, CN6*, CN7*, CN8*, CN9*, CN4, CN5, P4, P5, P6*.

- Book VII
- *Communes sententiae*: N19, N1.

$\bullet$ Grynaeus’s system of principles in Book I. Candale, however, stated that CN8* and CN9* should be interpreted as referring to any multiple and submultiple (cf. M3 and M4, in Jordanus and Campano); a similar remark is found in Proclus, *In Euclidem* 196–197. Candale also thought it necessary to add a few arithmetical principles, but found Campano’s list of them too long and reduced it to two axioms only.

- Francesco Maurolico, *Euclidis elementorum compendia*, 1567 (unpublished).

- Book I
- *Postulata*: P1–P2, P3, P4, P5, P6*.
- *Communes sententiae*: CN1, CN2–CN3–CN6*–CN7*, CN8*–CN9*, CN4, CN5–A6.

- Book V
- *Communes animi conceptiones*: R3, R4, R5, R6, R7, R8, R9, R10, R11, R12, R13, R14, R15, R16.

$\bullet$ Maurolico was highly dissatisfied with the editions of Euclid printed in the Renaissance, and from the 1530s on he raised several criticisms of both the Campano and Zamberti traditions. Between 1532 and 1534, he prepared new versions of Books II, V, VII, VIII, IX and X of the *Elements* which treated the Euclidean material with great freedom, and never went to print. In the same years, he also reworked the stereometric books of the *Elements* which were later published among Maurolico’s *Opuscula mathematica* (1575), and he wrote the *Arithmeticorum libri duo* (also published in 1575). None of these works presents any principle besides the definitions. In 1567, however, Maurolico prepared the abridged version of a number of Euclidean books, the *Euclidis elementorum compendia*, with the aim of merging together Zamberti’s and Campano’s versions (something that Lefèvre had only partially done in 1516) and adding a few new proofs and results. The latter edition, which also remained unpublished but circulated in manuscript form, has a set of axioms in Book I and V. The new axiomatization of the theory of proportions, in particular, represented an important starting point for further studies on the topic. Note that axioms R11 and R12 on the composition of ratios are completely new, and treat multiplications and divisions among ratios. The formal statement of A10 is that if ${\text {A}}{:}{\text {B}}={\text {D}}{:}{\text {E}}$ and $B{:}C=E{:}F$, then ${\text {A}}{:}{\text {C}}={\text {D}}{:}{\text {F}}$ (composition ’, or *ex aequali*). The axiom will be employed again by Benedetti, Viviani, Marchetti, and others. The edition of Maurolico’s text is in Garibaldi (2002). On Maurolico’s axiomatization of Book V, see Sutto (2000).

- Pedro Juan de Lastanosa (de Monzón), *Elementa Arithmeticae ac Geometriae*, Valencia, Huete, 1569.

- *Dignitates arithmeticae*: CN1, N7, N8, N9, A4, N20, N21, N22, CN2, CN3, CN6*, CN7*, N10, N14, N23, N24, N15, N25, N26, N16.
- *Petitiones seu postulata* [*arithmeticae*]: N6, N13, N27, N28, N4, N17.
- *Axiomata, seu dignitates* [*geometriae*]: CN1, CN2, CN3, CN6*, CN7*, CN8*, CN9*, CN4, CN5–A4.
- *Postulata* [*geometriae*]: P1, P2, P3, P4, P5, C1, P6*. (...)

$\bullet$ The book does not follow the Euclidean order and only covers a few elementary results in arithmetic and geometry. Monzón gave, however, two complete systems of principles for the two sections of his book, mostly drawing on Lefèvre d’Étaples (1496) and Fine (1532), but with a few additions of his own.

- Henry Billingsley, John Dee, *The elements of geometrie of the most ancient Philosopher Euclide of Megara, faithfully (now first) translated into the English tongue*, London, Daye, 1570.

- Book I
- *Peticions, or requestes*: P1, P2, P3, P4, P5, P6*.
- *Common sentences*: CN1, CN2, CN3, CN6*, CN7*, CN8*, CN9*, CN4, CN5.

- Book VII
- *Common sentences*: N7, M3, M4, N1, N3, N2, N29.

$\bullet$ The first complete translation of the *Elements* into English, with a Preface by John Dee. Billingsley adopted Campano’s system of principles in Book I, as emended by Tartaglia, and a modification of Campano’s axiomatics in Book VII. He added principle N29 of his own, stating the transitivity of the equality of ratios; it is an arithmetical version of *Elements* V, 11. The edition set the standards for further translations of Euclid into English. Thomas Rudd’s edition of the *Elements* (London, Leybourn 1651), for instance, followed quite closely Billingsley’s edition, and endorsed the same system of principles. On the Euclidean tradition in Britain, see Barrow-Green (2006).

- Federico Commandino, *Euclidis elementorum libri XV una cum Scholiis antiquis*, Pesaro, Franceschini, 1572.
- Italian version as *De gli elementi d’Euclide libri quindici con gli scolii antichi*, Urbino, Frisolino, 1575.

- Book I
- *Postulata* (*postulati, overo dimande*): P1, P2, P3, P4, [P5].
- *Axiomata, seu communes notiones* (*assiomi, overo communi notitie*): CN1, CN2, CN3, CN6*, CN7*, [CN8*], CN9*, CN4, CN5, P6*; PP14.

- Book V
- *Notiones communes* (*communi notitie*): M3, M4.

- Book VII
- *Petitiones* (*petitioni, overo dimande*): N12, N5, N6–N13.
- *Notiones communes* (*communi notitie*): M3, M4, N30, N31, N8, N32, N33, N35, N36, N34, N2, N3, N1.

- Book X
- *Notiones communes* (*communi notitie*): AA1, M9, M10, M11.

$\bullet$ A crucially important edition based on the Greek text by Grynaeus (1533) but double-checked against other, more reliable Greek manuscripts. Commandino remarked that P6* is not self-evident enough to be ranged among the axioms, and for this reason Campano had put it among the *petitiones*. He stated that P5 should be proven and removed from the list of postulates, and gave Proclus’s proof of it, relying on the new assumption PP14 (Latin ed. p. 19v; Italian ed. p. 21v). Moreover, Commandino noted that CN8* can be deduced by reiterated applications of CN2, and is thus redundant as a principle (cf. Proclus, *In Euclidis* 196–197). This notwithstanding, Commandino added axioms M3 (a generalization of CN8*) and M4 in Book V, taking them from Campano’s edition of Book VII; he also employed the same two principles in Book VII, just as Campano did. While in the Latin edition these axioms refer generally to “things” that are multiplied, in the Italian edition of the *Elements* Commandino stated a modified version of axioms M3 and M4 in Book V, which only applies to continuous magnitudes, and another version of the same axioms in Book VII, which only applies to numbers. In Book X, Commandino added a version of Archimedes’s Axiom (AA1) and three new axioms (M9, M10, M11) which are modifications of the arithmetical axioms N3, N1 and N2.

- Christoph Clavius, *Euclidis elementorum libri XV*, Roma, Vincenzo Accolto, 1574.

- Book I
- *Petitiones, sive Postulata*: P1, P2, P3, A7.
- *Communes notiones, sive Axiomata, sive Pronunciata, sive Dignitates*: CN1, CN2, CN3, CN6*, CN7*, CN8*, CN9*, CN4, CN5, P4, [P5], P6*, I5, M1, M2, M12, M13, A4, M14.

- Book V
- *Axioma*: R17.

- Book VII
- *Postulata, sive petitiones*: N12, N5, [N6–N13].
- *Axiomata, sive pronuntiata*: M3, M4, N30, N31, N32, N33, N34, N35, N36, N2, N3, N1.

- Book X
- *Postulatum, sive petitio*: AA1.
- *Axiomata, sive pronunciata*: M11, M9, M10.

$\bullet$ Another very important edition, which remained, for two centuries, the basis of many further axiomatizations of the *Elements*. Clavius claimed that all the principles can be proven from the definitions. Among them, P5 is complex enough to have to be removed from the list of axioms and to be considered a theorem. Clavius stated that M1 and M2 are taken from Pappus, quoted in Proclus, *In Euclidem* 197 (but they were already known to Campano through Gerardo da Cremona’s translation of An-Nayrīzī’s commentary). Axiom R17 (the existence of the fourth proportional) is added at the beginning of Book V. Clavius mostly followed Commandino’s system of principles in Books VII and X. Principle N6–N13 is not listed among the arithmetical postulates, but added in a following remark. Principle AA1 was an axiom in Commandino and became a postulate in Clavius. See below the different list of geometrical principles in the 1589 edition of Clavius’s masterwork.

- Rodrigo Zamorano, *Los seis libros primeros dela geometria de Euclides*, Sevilla, La Barrera, 1576.

- Book I
- *Peticiones*: P1, P2, P3, P4, P5.
- *Comunes sentencias*: CN1, CN2, CN3, CN6*, CN7*, CN8*, CN9*, CN4, CN5, P6*.

$\bullet$ Zamberti’s system of principles. The first translation of the *Elements* into Spanish.

- Giovan Battista Benedetti, *In quintum Euclidis librum*, in *Diversarum speculationum mathematicarum, & physicarum Liber*, Torino, Belvilacqua, 1585.

- Book V
- *Postulata*: R11–R12, CN3–CN2, R3–R5, R4–R6, R8, R9, R10, R18, R19, R20, R21, R22.

$\bullet$ A reworking of Book V of the *Elements* with a new system of principles, which exerted a certain influence on seventeenth-century axiomatizations of the theory of proportions. Benedetti’s first and second axioms put together the standard Euclidean common notions on the composition of magnitudes (their sum and difference), with Maurolico’s new axioms on the composition of ratios (their multiplication and division), with the clear aim of justifying the latter by the reference to the former. Benedetti’s following axioms are simply the Euclidean propositions of *Elements* V, 7–13. They are followed by R20 that makes explicit the connection between ratios among magnitudes and ratios among numbers, and by two more axioms that seem to define continuous proportion. On Benedetti’s theory, see Bodriga (1926) and Giusti (1993).

- Francesco Patrizi, *Della nuova geometria*, Ferrara, Baldini, 1587.

- [Book I]
- *Supposizioni*: S1, S2, S3, S4, S5.
- *Assiomi*: A8, A9, A10, CN5, A11, A12.

$\bullet$ This book is not, properly speaking, an edition of the *Elements*, but rather an independent logical treatment of the first Euclidean results regarding straight lines, angles and triangles (covering more or less the Euclidean material up to *Elements* I, 29). The book is important inasmuch as it is the first treatise to discuss space as the object of geometry, as well as for developing a quasi-formal treatment of this field (cf. Herlin & Dasypodius, 1566). Patrizi’s geometrical “suppositions” are mostly taken from his own essays on the metaphysics of space (*De rerum natura libri II priores. Alter de spacio physico, alter de spacio mathematico*, Ferrara, Baldini 1587), while his “axioms” are modified Euclidean common notions.

- Christoph Clavius, *Euclidis elementorum libri XV*, Roma, Bartolomeo Grassio, $1589^{2}$.

- Book I
- *Petitiones, sive Postulata*: P1, P2, P3, A7.
- *Communes notiones, sive Axiomata, sive Pronunciata, sive Dignitates*: CN1–A13–A14, CN2, CN3, CN6*–M15, CN7*–M16, CN8*–M17, CN9*–M5, CN4, CN5, I5, I6, P4, [P5], P6*, M1, M2, M12, M13, A4, M14.

- Book V
- *Axioma*: R17.

- Book VII
- *Postulata, sive petitiones*: N12, N5, [N6–N13].
- *Axiomata, sive pronuntiata*: M3, M4, N30, N31, N32, N33, N34, N35, N36, N2, N3, N1.

- Book X
- *Postulatum, sive petitio*: AA1.
- *Axiomata, sive pronunciata*: M11, M9, M10.

$\bullet$ With respect to the 1574 edition, Clavius’s second edition added, in Book I, a number of common notions on the comparison of magnitudes, as well as the new axiom I6, which Clavius employed in the proof of P5. The main difference between the two editions is given, in fact, by the lengthy discussion of the Parallel Postulate, that was engendered by Clavius’s new acquaintance with the work of Nasīr ad-Dīn (see below). The following editions of Clavius’s commentary (the last one being in Clavius’s *Opera mathematica* from 1612) made no further changes in the system of principles.

- Nas ī r ad-Dī n at-Tū s ī, *Euclidis elementorum geometricorum libri tredecim ex traditione doctissimi Nasiridini Tusini*, Roma, Typographia Medicea, 1594.

- Book I
- *al-usul al-mawdu’a*: P6*, P2, P4, P5.
- *al-’ulum al-muta’arifa*: CN1, CN2, CN3, CN6*–CN7*, CN8*, CN9*, CN4, CN5.

$\bullet$ The Persian mathematician and scientist Nasīr ad-Dīn at-Tūsī (1201–1274) wrote a commentary on the *Elements* (largely inspired by Ibn al-Haytham’s work), as well as an important treatise on the proof of the Parallel Postulate. After his death a disciple of his compiled, in 1298, a further commentary on Euclid that drew on Nasīr ad-Dīn’s work and was later attributed to Nasīr ad-Dīn himself. Many centuries later, three manuscripts containing the two commentaries (the one by Nasīr ad-Dīn, and the other by his disciple, later called the pseudo-Tūsī) came into possession of the Medici family, along with many other Arabic works. A publishing house, the Oriental Press or Typographia Medicea, was then established in Rome under the direction of Giovan Battista Raimondi (1536–1614) with the aim of publishing several of these works. The commentary of the pseudo-Tūsī (still attributed to Nasīr ad-Dīn) was published in Arabic (with a Latin title) in 1594. This famous Arabic edition could not exert much influence in Europe: yet Nasīr ad-Dīn’s proof of the Parallel Postulate was read (still in manuscript form) by Clavius, engendering the 1589 edition of his commentary (see above), and was later translated into Latin by Edward Pocock (1661) and published as an appendix to Wallis’s *Algebra* (1693). This was the only fragment of the pseudo-Tūsī’s commentary that enjoyed some diffusion in a Western language. His more general system of principles in Book I, apparently, was not able to affect any further study on the topic. While the list of axioms of this edition is quite standard, the geometrical postulates are widely discussed and connected with metaphysical considerations; P1 and P3 on the construction of lines and circles are expunged from the list of proper principles. On Nasīr ad-Dīn’s commentary on Euclid, its sources, and its relation with the pseudo-Tūsī’s edition, see de Young (2003, 2009). A complete discussion of the Medici edition is to be found in Cassinet (1986).

- Jan Pietersz Dou, *De ses eerste Boecken Euclidis*, Leiden, Bouwensz, 1606.

- Book I
- *Eenige stucken tot dese konst behoorende*: P1, P2, P3.
- *Ghemeene bekantenissen*: CN1, CN2, CN3, CN6*, CN7*, CN8*, CN9*, CN4, CN5, P4, P5, P6*.

$\bullet$ Grynaeus’s system of principles. The first translation of the *Elements* into Dutch. Dou’s book follows the German version by Xylander (1562) with a few modifications. The same system of principles was to be used by the following Dutch edition of the *Elements* edited by Frans van Schooten, Sr. (*De propositien vande XV. Boucken der Elementen Euclidis*, Leyden, Basson, 1617).

- Pietro Antonio Cataldi, *I primi sei libri de gl’Elementi d’Euclide ridotti alla Prattica*, Bologna, Cochi, 1613.

- Book I
- *Petitioni, overo domande*: P1, P2, P3, [P4], [P5], P6*.
- *Comuni concessioni, o comuni notitie*: CN1, CN2, CN3, CN6*, CN7*, [CN8*], CN9*, CN4, CN5–A4.

- Book V
- *Petitione*: M14.
- *Comuni concessioni*: CN1, CN2.

$\bullet$ Cataldi claimed that P4, P5 and CN8* (but probably also CN9*) can be proven and should not be counted among the principles.

- Denis Henrion, *Les quinze livres des elements d’Euclide traduicts de Latin en François*, Paris, Joallin, 1615.

- Book I
- *Petitions, ou demandes*: P1, P2, P3.
- *Communes sentences*: CN1, CN2, CN3, CN6*, CN7*, CN8*, CN9*, CN4, CN5, P4, P5, P6*.

- Book VII
- *Communes sentences*: M3, M4, N30, N31, N8, N32, N34, N35, N36, N2, N3, N1.

- Book X
- *Demande*: AA1.
- *Communes sentences*: M11, M9, M10.

$\bullet$ Grynaeus’s system of principles in Book I, and a simplification of Clavius’s (and Commandino’s) principles in the other books. The volume enjoyed great success and was reprinted several times.

- Pierre Herigone, *Cursus mathematicus*. *Cours mathematique*, Paris, Le Gras, 1634.

- Book I
- *Petitiones, sive Postulata* (*Petitions, ou Demandes*): P1, P2, P3, A7.
- *Communes notiones, sive Axiomata, sive Pronunciata, sive Dignitates* (*Communes notions, ou communes sentences, ou maximes*): CN1, A15, A13, A14, A16–A17, A18, A19, CN2, CN3, M18, CN6*, M6, M15, CN7*, M7, M16, CN8*, M19, M20, M21, CN9*, M22, M23, M24, CN4, CN5, A20, I5, I6, P4, E4, P5, P6*, I7, I8, M1, M2, M12, M13, A4, M25, M14, M26, A21.

- Book V
- *Axioma*: M27.

- Book VII
- *Postulata*: N12, N5, N37.
- *Axiomata*: N38, A22, N30, N31, N32, N33, N34, N35, N36, N2, N3, N1.

- Book X
- *Postulatum*: AA1.
- *Axiomata*: M11, M9, M10.

$\bullet$ A complete reworking of the system of principles of the *Elements*, Herigone’s work was a milestone in the development of Euclidean axiomatics, and several of its principles were employed many times in the following years. The intended interpretation of a few of the added principles is the following. Axiom A15 should guarantee the transitivity of equality, and refers to things which are equal (not just “the same things”). Axiom A18 is a principle of substitutivity *salva aequalitate*: if ${\text {A}}+{\text {B}}={\text {C}}+{\text {D}}$ and ${\text {B}}={\text {D}}$, then ${\text {A}}+{\text {D}}={\text {C}}+{\text {B}}$. The example that Herigone gives for A19 is the following: if AF is the square of AB, CG is the square of CD, and AF is equal to CG, then by A19 the square of AB is equal to the square of CD. The example of A21 is that if ${\text {A}}>{\text {B}}$ and ${\text {A}}<{\text {B}}$ are both false, then ${\text {A}}={\text {B}}$; the axiom was later to be stated in this form (A24, in Schott 1661). Axiom M27 means that if A is an equimultiple of B as E is of F (i.e., ${\text {A}}{:}{\text {B}}={\text {E}}{:}{\text {F}}$), and C is an equimultiple of D as E is of F, then A is an equimultiple of B as C is of D. Axiom E4 on the right angles seems to come from Proclus, *In Euclidis* 189, where it is said that Pappus had denied this principle, stating that curvilinear right angles may be different to one another.

- Christoph Grienberger, *Euclidis sex primi elementorum geometricorum libri cum parte undecimi ex majoribus Clavij commentarijs in commodiorem forma contracti*, Graz, Widmanstad, 1636.

- Book I
- *Petitiones, seu Postulata*: P1, P2, P3.
- *Axiomata, seu Pronunciata*: CN1, CN2, CN3, CN6*, CN7*, CN8*, CN9*, CN4, CN5, I9, P4, P5, P6*, A4, M14.

$\bullet$ Another Jesuit edition of the Elements, Grienbergers’s book presented a simplification of Clavius’s axiomatics, which substituted I5 and I6 with axiom I9, and deleted M1, M2, M12, and M13 from Clavius’s list. Grienberger had no axioms in Book V.

- Marin Mersenne, *Universae geometriae, mixtaeque mathematicae synopsis*, Paris, Bertier, 1644.

- Book I
- *Postulata*: P1, P2, P3, P4, P5.
- *Axiomata, seu Communes notiones*: CN1, CN2, CN3, CN6*, CN7*, CN8*, CN9*, CN4, CN5, P6*.

- Book VII
- *Petitiones*: N12, N5, N6–N13.
- *Communes notiones*: M3, M4, N30, N31, N8, N32, N33, N35, N36, N34, N2, N3, N1.

- Book X
- *Communes notiones*: AA1, M9, M10, M11.

$\bullet$ Mersenne’s treatise of mathematics offers a simplification of Commandino’s axiomatics, which includes no axioms in Book V and does not further discuss the principles in Book I. Mersenne’s book only states the theorems, without giving the proofs of them. The book also contains several other mathematical works given in a similarly abridged form.

- Claude Richard, *Euclidis elementorum geometricorum libros tredecim*, Antwerp, Verdus, 1645.

- Book I
- *Petitiones, sive Postulata*: P1, P2, P3, A7, T1, T2.
- *Axiomata, seu Pronunciata, seu Dignitates, seu Communes notiones*: CN1–A13–A14–A15–A16–A17, CN2, CN3, CN6*–M15, CN7*–M16, CN8*–M17, CN9*–M5, CN5–A6, CN4–T3, I5, I6, P4, P5–PP15, P6*, M1, M2, M12, M13, A4, [M14], T4, C3, C4, I10, C5, C6, C7, C8, C9, T5.

- Book V
- *Axiomata*: R17, A23, M28; [A4, CN2, CN6*, CN3, CN1, CN7*, A13, M1].

- Book VII
- *Petitiones*: N12, N5.
- *Axiomata*: M3, M4, N30, N31, N32, N33, N34, N35, N36, N2, N3, N1.

- Book X
- *Petitio*: AA1.
- *Axiomata, sive pronunciata*: M11, M9, M10.

$\bullet$ Jesuit textbook. Richard enriched Clavius’s axiomatization, which he took as a starting point for his emended edition of Euclid. Axioms T1 and T2 are the first attempt to explicitly axiomatize the use of movement in geometry, and depend on Clavius’s discussion with Peletier (in Clavius 1589 and following editions, commenting *Elements* III, 16). The movements described by T2 are translations and rotations around a point or an axis. The rigidity of the figure during movement is intended to be guaranteed by the new axiom T5. Axioms CN8*–M17 and CN9*–M5 are in fact stated in a more general way which applies to any multiple and submultiple (not just the double and the half). Richard gave a complete proof of his T3, relying on superposition (but, following Clavius, he thought that a proven axiom is still an axiom). Richard stated that Proclus’s and Clavius’s proofs of P5 rely on more obscure principles than P5 itself, and thus he claimed that it should be regarded as an axiom rather than as a theorem (as Clavius had stated). Principle PP15 comes from Ptolemy’s proof of *Elements* I, 28 (see Proclus, *In Euclidis* 362–363): If two straight lines form internal angles equal to two right angles on the one side, they cannot meet; otherwise there would be no reason why they should not meet on the other side as well (where they also form internal angles equal to two right angles), and this is impossible as they would enclose a space. Axiom PP15 states the symmetry requirement. Richard expunged M14 from the list of principles, since it was introduced by later commentators to make explicit an assumption of the Euclidean proof of *Elements* III, 20, while Richard gave another proof of the same proposition that does not recur to such a principle. Axioms C5, C6, C7, and C8 are about “Line–Circle” continuity. Axiom C9 is a form of the so-called Pasch’s Axiom. The system of principles employed by Richard in Book V largely consists in new definitions, but he also had a list of eleven axioms that simply repeat (with small changes) a few of the common notions of Book I, adapting them to continuous magnitudes and ratios; the only principles to which nothing corresponds in Book I, besides R17, are A23 and M28; the other principles are modified versions of A4, CN2, CN6*, CN3, CN1, CN7*, A13, M1.

- Evangelista Torricelli, *De proportionibus liber*, 1647 (unpublished).

- *Suppositiones et Axiomata*: R10, R3–R4, R5–R6, R23, R24, R20.

$\bullet$ Torricelli’s treatise on the theory of proportions reworked Galileo’s own theory, which was dictated to him by Galileo and first published by Viviani in 1674 (as the Fifth Day of the *Discorsi*). Even though Torricelli’s book was never published, it enjoyed some manuscript circulation, and influenced several writers. Axioms R23 and R24 give in fact a different characterization of proportionality than the Euclidean definition. Torricelli’s book on proportions was first published in Vol. 1 of Torricelli’s *Opere* (see p. 306), and can now be read with a critical edition and a commentary in Giusti (1993).

- John Wallis, *In Elementa Euclidis Praelectiones*, 1651 (unpublished).

- Book I
- *Postulata*: P1, P2, P3.
- *Axiomata*: CN1, CN2, CN3, CN6*, CN7*, CN9*, CN8*, CN4, CN5, P4, [P5], P6*.

$\bullet$ The Bodleian Library preserves a manuscript with Wallis’s Oxford lectures on Euclid’s *Elements* from 1651. They are still unpublished, and contain a remarkable amount of notes and philosophical discussions on the principles and theorems of Book I of the *Elements*. Among them, we find Wallis’s alleged proof of the Parallel Postulate, that he published in the essay *De postulato quinto*, appended to the 1693 Latin edition of his *Algebra*. In his lectures, Wallis made use of Grynaeus’s text and system of principles, even though he discussed Clavius’s list as well, stating that the axioms added by the Jesuit are rather theorems (as a matter of fact, Wallis provided a few proofs of other axioms as well).

- Giovanni Ricci, *De gli Elementi di Euclide li primi sei Libri*, Bologna, Longhi, 1651.

- Book I
- *Postulati, overo Dimande*: P1, P2, I11, S6, T6, T7.
- *Assiomi, overo communi sentenze*: CN1, A13, A14, CN2, CN3, CN6*, M15, CN7*, M16, CN8*, CN9*, CN4, CN5, P6*, A4, P4, I6, T8, T9, PP2, A21.

- Book II
- *Assioma*: A1.

$\bullet$ The aim of the author was to produce a text for students. Postulate I11 is a modification of P3, while T6 is intended to guarantee the possibility of reproducing any figure (Ricci employed it in *Elements* I, 5 in order to construct an isosceles triangle congruent with the given one), and T7 is a version of Richard’s T1. In Ricci, each axiom A13 and A14 is split in two different principles (referring to the greater or the lesser relation respectively). Axiom T8 is a simplified version of Richard’s T3. Axiom PP2 is a substitute for the Parallel Postulate (P5).

- Andreas Tacquet, *Elementa geometriae planae et solidae*, Antwerp, Meurs, 1654.

- Book I
- *Postulata*: P1, P2, P3.
- *Axiomata*: CN1–A13, CN2, CN3, CN6*, CN7*, CN8*–CN9*, CN4, T10–T11, CN5, P4, PP3, PP4, P6*, [I5].

- Book V
- *Axioma*: R25.

$\bullet$ Jesuit textbook. It only contains Books I–VI and XI–XII of the *Elements*, while Books VII–IX were published by Tacquet in 1656 (see below). Tacquet’s edition of the *Elements* modified several proofs and offered a new theory of proportions in Book V, as well as a proof of the Parallel Postulate. It enjoyed a broad circulation and was regarded as a model for subsequent reformulations of elementary geometry. Tacquet added axioms T10 and T11 as partial converses of CN4 (cf. Richard’s T3). He explicitly recused P5 (the Parallel Postulate) from the list of axioms. Tacquet’s new theory of parallels is grounded on the definition of parallel lines as equidistant straight lines, and on his new axioms PP3 and PP4. In fact, each of these three principles is enough to guarantee the provability of the Parallel Postulate (P5). Assuming the existence of equidistant straight lines (as Tacquet did) is in fact equivalent to assuming P5 itself. Axioms PP3 and PP4 are false in hyperbolic geometry as well, and from each of them P5 can be deduced, while both of them can in turn be proven, assuming the existence of equidistant straight lines. Tacquet’s intended interpretation of PP4 is that *every* perpendicular to a straight line is also perpendicular to a parallel to it (in hyperbolic geometry, ultraparallel lines only have *one* common perpendicular). After the statement of I5, Tacquet gave a proof of it. The axiom R25 of Book V is simply the existence of the fourth proportional already employed by Clavius and others, in a clearer wording. In dealing with irrational magnitudes and ratios in Book V, Tacquet added three more axioms, which he took to be provable and which had in fact been proven by Grégoire de Saint Vincent (Tacquet’s teacher) in his *Opus geometricum quadraturae circuli et sectionum coni* (1647). Further editions of Tacquet’s book (among which an emended version from 1665) did not modify the system of principles. The book enjoyed a broad diffusion in English translation (see for instance William Whiston’s edition from 1714).

- Isaac Barrow, *Euclidis Elementorum Libri XV breviter demonstrati*, Cambridge, Nealand, 1655.

- Book I
- *Postulata*: P1, P2, P3.
- *Axiomata*: CN1, CN2, CN3, CN6*, CN7*, CN8*, CN9*, CN4 [T10, T12], CN5, I5, I6, P4, P5, P6*, M1, M2, M12, M13, A4, M14.

- Book V
- *Axioma*: M27.

- Book VII
- *Postulata:* N12, N5, N37.
- *Axiomata*: N38, A22, N30, N31, N32, N33, N34, N35, N36, N2, N3, N1.

- Book X
- *Postulatum*: AA1.
- *Axiomata*: M11, M9, M10.

$\bullet$ A famous abridged edition of the *Elements*, with new proofs by Barrow. Barrow’s system of principles in Book I is a slight simplification of Clavius’s 1589 edition. Barrow stated that CN4 can be converted in the case of straight lines and angles (cf. Tacquet’s axiom T10, to which he added T12). This notwithstanding, in his *Lectiones mathematicae* from 1665 he explicitly criticized Tacquet, claiming that Euclid’s CN4 may be converted in all cases. The meaning of the converse of CN4, according to Barrow, should be that “Things that are equal to one another *may* coincide with one another”, where the modal verb should express the possibility to dissect the first thing into pieces (and even in infinitesimal or indivisible pieces) and recompose them again in another thing which is congruent to the second. He wanted, in sum, to ground the equality of measure on the congruence of parts (see in particular his *Lect.* XIII). Barrow’s lectures were posthumously published in 1685 and reprinted in a celebrated edition by Whewell (Cambridge University Press, 1860). Barrow’s system of principles in Books V, VII, and X follows Herigone (1634).

- Blaise Pascal, *Introduction à la géométrie*, around 1655 (unpublished).

- [Book I]
- *Principes*: S8, S9, S10, S11, E5, S12.
- *Théorèmes connues naturellement*: S13, E8–S14, S15, S16, E6, I3, C10, C11, C12, C13, C14, C15.

$\bullet$ The sketch of an introduction to geometry written by Pascal around 1655. Pascal went no further in the project, nor did he publish his introduction, since Arnauld was then working on his *Nouveaux éléments* (published in 1667) and Pascal decided that a further edition would have been useless. Pascal’s draft was later copied by Leibniz and was found among Leibniz’s papers in Hannover (the original manuscript is now lost); it is published in Pascal, *Oeuvres*, vol. 9, pp. 292–294. Axiom S16 is the converse of *Elements* III, 24. Axiom E6 is taken from a Euclidean assumption in *Elements* I, def. 18: there had been several attempts to prove this proposition, from Antiquity to the Early Modern Age.

- Andreas Tacquet, *Arithmeticae theoria et praxis*, Leuven, Coenestenius, 1656.

- Book VII
- *Axiomata*: M3, M4, N30, N31, N8, N9, N34, N36, N5, N2, N3, N1.

$\bullet$ Jesuit textbook. Tacquet’s edition of Books VII, VIII, and IX of the *Elements*, complementing his edition of the geometrical books, already appeared in 1654 (see above). A treatise on practical arithmetic follows the Euclidean books.

- Giovanni Alfonso Borelli, *Euclides restitutus, sive prisca geometriae elementa*, Pisa, F. Onofrio, 1658; Roma, Mascardi, $1679^{2}$. Italian ed. *Euclide rinnovato ovvero gli antichi elementi della geometria,* Bologna, Ferroni, 1663.

- [Book I]
- *Petitiones, seu Postulata*: P1, P2, P3, A7.
- *Axiomata, seu Pronunciata*: CN1–A13–A14, CN2, CN3, CN6*–CN7*, CN8*–CN9*, M29, CN5, A4, CN4, I9, P6*, E7, C16; PP5.

- [Book II]
- *Axioma*: E8–E9.

- [Book III]
- *Axiomata*: M9, R17, M30, R26, R27.

- [Book V]
- *Axioma*: E10.

- [Book VI]
- *Postulata*: I12, I4.
- *Axiomata*: I13, I14, I15, I8, I16.

- [Book IX]
- *Axiomata*: M2–M13, M12.

$\bullet$ Borelli’s *Euclides restitutus* attempted a new foundation of the *Elements* through the introduction of several new axioms, new proofs and a new ordering of the classical theorems. Book I deals with elementary geometrical theorems similar to *Elements* I, while Book II is the theory of the circle (similar to *Elements* III); Book III discusses the theory of proportions (*Elements* V); Book V a few results in inscribing and circumscribing polygons (cf. *Elements* IV) and their similarity (*Elements* VI); Book VI has several points in common with *Elements* XI; Book VIII deals with arithmetic (*Elements* VII, VIII and IX). Book IX deals with irrational quantities (with a few connections with *Elements* X). In Book I, axiom M29 is a simple extension of N2. Axioms E7 and C16 are used by Borelli to prove *Elements* I, 1, which, in his opinion, was in need of a further continuity assumption. Axiom E7 is used in the following Books as well. Axiom PP5 is added at p. 32, just before proving P5 and *Elements* I, 29; it is in fact equivalent to the Parallel Postulate. Borelli’s definition of parallel lines is that of straight lines such that any perpendicular to one of them is also a perpendicular of the others and such that all these perpendiculars are equal to one another (a property that in fact follows from the first condition); axiom PP5 is intended to guarantee their existence. In Book III, axiom R17 on the existence of the fourth proportional is split into two different axioms. The system of principles in Book III, completed with a set of new definitions for equiproportionality, is intended to radically reform the Eudoxian theory of proportions (see Palladino 1991; Giusti 1993). Axioms I13, I14 and I15 are extensions of the usual axioms on the straight line, while I8 is explicitly referred to *Elements* XI, 1, where it is implicitly assumed by Euclid; I16 is referred to *Elements* XI, 2. Borelli had no axioms on arithmetic in his Book VIII. Borelli’s work was translated into Italian by Domenico Magni in 1663, in an abridged edition that did not include Books VIII and IX. This edition had (in the remaining Books) the same system of principles than the first edition, with the only exception that Magni, contrary to Borelli’s opinion, claims that CN4 can be converted (i.e.: everything which is equal can be superposed; cf. the edition by Barrow, 1655, above). A second Latin edition of the work appeared in 1679, again without Books VIII and IX. In it, Borelli eliminated the continuity principle C16 from the axioms of Book I, supplying a new (less rigorous) proof of *Elements* I, 1 which only employs E7.

- Claude François Milliet Dechales, *Huict Livres des Elements d’Euclide rendus plus faciles*, Lyon, Coral, 1672.

- Book I
- *Demandes*: P1, P2, P3.
- *Axiomes, ou Principes*: CN1–A13, CN2, CN3, CN6*, CN7*, CN8*–CN9*, CN4, T10–T12, CN5, P4, PP7, P6*, I9.

- Book V
- *Axioma*: R25.

$\bullet$ Jesuit textbook. This edition only contains Books I–VI and XI–XII of the *Elements* (cf. Tacquet, 1654), thus eliminating the arithmetical treatises (VII–IX), the tenth book on irrational magnitudes, and the thirteenth book on the Platonic solids; this new arrangement of the matter was later followed in many eighteenth-century editions. Dechales’s system of principles derived from Tacquet (1654), but substituted a new axiom PP7 to prove the Parallel Postulate (it is in fact equivalent to it). Dechales’s book enjoyed several reprints and translations. A Latin version of it was inserted into Dechales’s monumental *Cursus seu Mundus mathematicus*, the first edition of which was printed in 1674, followed by an important posthumous enlarged edition in 1690. The system of geometrical principles remained, however, unchanged in these later editions. The *Cursus* also added a few books on arithmetic, which are introduced by five arithmetical axioms. These books, however, do not follow the Euclidean development and their (very practical) principles only concern the decimal number system.

- Caspar Schott, *Cursus mathematicus, sive Absoluta omnium mathematicarum disciplinarum encyclopaedia*, Würzburg, Hertz, 1661.

- Book I
- *Petitiones, sive Postulata*: P1, P2, P3, A7.
- *Axiomata, sive Pronunciata*: CN1, CN2, CN3, CN6*, CN7*, CN8*, CN9*, CN4, CN5, P4, [P5], P6*, I5, I6, I7, I8, M1, M2, M12, M13, A4, M14, M26, A24, E8, C17, A16.

$\bullet$ Jesuit textbook. Book III of Schott’s *Cursus* includes the first six Books of the *Elements*. Schott’s axioms for Euclid’s Book I, mostly follow Clavius, with a few additions from Herigone and a couple of new principles. Schott claimed to have proven P5 with a simple (and wrong) conversion of *Elements* I, 27 (p. 73). He had no axioms in *Elements* V.

- Antoine Arnauld, *Nouveaux Élémens de géométrie*, Paris, Savreux, 1667.

- [Book I]
- *Axiomes de l’égalité et inégalité*: CN5, A4, CN1, CN2, CN3, CN7*, CN6*, M3, M4.

- [Book II]
- *Axiomes*: R20, R13, R29, R30, M31, R31.

- [Book V]
- *Axiomes*: E11, P1, P2, I5, I17, PP6; P3, T13, E8, E12, S16–E17, E13, S17; E14.

- [Book XIII]
- *Axiomes*: E15, E16, N39.

$\bullet$ Arnauld’s text on elementary geometry was one of the most important books on the subject in the seventeenth century. It did not follow the Euclidean order or axiomatics, but wanted to improve upon it (cf. Tacquet’s and Borelli’s works). It was written around 1655–1656 in connection with Pascal’s *Introduction* (see above), but printed only in 1667; a second, modified edition was published in 1683 (see below). It was clearly influenced by Clavius (1589), Herigone (1634), and Tacquet (1654), among others. Book I deals with arithmetic and adds several unproven rules to the Euclidean common notions. Books II–IV deal with the theory of proportions and incommensurable magnitudes, and begin with an axiomatization of the classical Eudoxian theory (which was to be modified in the second edition of the volume). Book III ends with two simpler axioms on equal ratios which are, however, not employed in the book, and are removed in the second edition. Books V–XV deal with plane geometry, all axioms being at the beginning of Book V with the exception of the three used in Book XIII for the calculation of areas. The interpretation of axiom R29 is that $n{\text {A}}{:}m{\text {A}}=n{\text {B}}{:}m{\text {B}}$ (for any A, B), while R30 states that $n{\text {A}}{:}n{\text {B}}=m{\text {A}}{:}m{\text {B}}$; they are generalizations of R20 ($n{\text {A}}{:}{\text {A}}=n{\text {B}}{:}{\text {B}}$) and R13 ($n{\text {A}}{:}n{\text {B}}={\text {A}}{:}{\text {B}}$), respectively. Axiom I17 is a formulation of the classical P6*, but has in Arnauld a simpler statement. Axiom PP6 is a substitute for the Parallel Postulate (it rules out asymptotic parallel lines); Arnauld, however, also attempted a proof of P5 independent from this axiom in Book VI. The intended meaning of E12 is that the “degrees” of a smaller circumference are smaller than the degrees of a larger circumference, since both lines are divided in the same number of degrees (i.e., 360). Axiom E14 is connected with Arnauld’s definition of a perpendicular, which is a line that satisfies the condition of the axiom (in two points).

- Gilles-François de Gottignies, *Elementa geometriae planae*, Roma, Angelo Bernabò, 1669.

- *Postulata*: P1, P2, P3.
- *Axiomata*: CN1–A13–A14, CN2, CN3, CN6*, CN7*, CN8*–CN9*, CN4, T10–T11, CN5, P4, P6*, I5, R32, R28.

$\bullet$ Gottignies’s book is not, properly speaking, an edition of the *Elements*, but rather an introduction to geometry following Euclid as closely as possible. Gottignies assumed that P5 had been proven by Clavius.

- Honoré Fabri, *Synopsis geometrica*, Lyon, Molin, 1669.

- *Axiomata*: CN1–CN8*–CN9*, CN2–CN3–CN6*–CN7*, CN5–A4–M14–A24, R33, I17–I5, T14, CN4, R34, R35, R36.
- *Postulata*: P1, P3, P2–T15, A7, T2, S18, T16.

$\bullet$ Jesuit textbook. Once again, this is not, properly speaking, an edition of the *Elements*, but rather an introduction to geometry following Euclid as closely as possible. I have simplified somewhat Fabri’s verbose axioms R33, R35, R36. Axiom T14 is introduced in order to employ motion in geometry. Fabri claimed that CN4 can be unrestrictedly converted. Fabri’s constructive postulate T15 is stated among a list of operations that can be accomplished with geometrical tools and it does not seem to have real foundational relevance. Axiom T2 is again employed in order to make use of rigid motion in geometry, while the new T16 was intended to guarantee non-rigid motion in a few occurrences. The Parallel Postulate is proven from the definition of equidistant lines (assumed as a real definition).

- Vincenzo Viviani, *Quinto Libro degli Elementi di Euclide, ovvero Scienza Universale delle Proporzioni spiegata colla dottrina del Galileo*, Firenze, Condotta, 1674.

- *Assiomi, ovvero Comuni notizie*: R38, R39, R35, R36, R16–R37, R10, R4, R40, R11.
- *Domanda*: R17.

$\bullet$ Since Torricelli’s book on the theory of proportion (written in 1647) had not been published, this edition by Viviani of Galileo’s Fifth Day of the *Discorsi* is the first printed essay containing Galileo’s reform of Book V of the *Elements*. It is prefaced by Viviani’s own attempt to render more rigorous the theory of proportions, which includes ten new principles. Viviani was to publish a fuller edition of the *Elements* in 1690, in which, however, he was to adhere to a more standard axiomatization, following Zamberti for the system of principles in Book I and including no axioms in Book V.

- Gilles Personne de Roberval, *Éléments de géométrie*, around 1673–1675 (unpublished).

- [Book I]
- *Axiomes*: A4, CN5, CN1, CN2.
- *Postulats*: S19, I18, I19, I20, I21, I22, I23, T2, I24, P1, A7, A25.

- [Book II]
- *Axiomes*: CN4, T17, E1.
- *Postulat*: C18.

- [Book III]
- *Postulats*: P3, AA4, C19.

- [Book IV]
- *Axiome*: PP8.

- [Book VIII]
- *Axiome*: E19.

$\bullet$ Roberval died before publishing the text, which is a new systematization of elementary geometry (plane and solid) in eight books. Several postulates in Book I are more of a philosophical than of a geometrical nature, while others have a more recognizably mathematical appearance. Among them, S19 and I22 together should guarantee the existence of all mathematical objects. Principles I18–I21 may also be employed in a theory of indivisibles. Axiom I23, stating the possibility to include a “space” between any two bounded regions, seems to state a modern separability property (e.g., the so-called topological separability axiom $\hbox {T}_{4}$, stating that space is “normal”). Axiom I24 is the condition for the definition of a straight line as a rotational axis. Book II has, besides the axioms, two postulates: The first of them is, however, incompletely formulated; the second, C18, may be regarded as a form of Pasch’s Axiom. Postulate AA4 in Book III is a form of the Archimedean Axiom. Axiom PP8 in Book IV is a substitute for P5. The edition of Roberval’s manuscript is in Jullien (1996).

- Jean Prestet, *Elemens des mathematiques, ou principes generaux de toutes les sciences qui ont les grandeurs pour objet*, Paris, Pralard, 1675.

- *Axiomes*: A26, M32, A4, CN5, M33, CN1, CN2, CN3, CN6*, CN7*; M3, M4, N40, M11, M10, N41, N42.

$\bullet$ Jesuit textbook. Prestet’s book is a treatise on arithmetic and algebra with a few geometrical examples. It does not follow the demonstrative order of the *Elements*, but contains a few axioms in the first two Books which may be considered an extension of the Euclidean Common Notions. Prestet also added several other unproven practical principles (which he called “*demandes*” or simply “*regles*”) that teach how to manipulate the algebraic symbolism; they are not included here, as they are too numerous and depart widely from the Euclidean style. A second edition appeared in 1689 (see below).

- Mercator (Nikolaus Kauffmann), *Euclidis Elementa Geometrica Novo Ordine ac Methodo fere demonstrata*, London, Martyn, 1678.

- *Axiomata*: I5–P6*, P4, PP9, CN4–T10–T12, CN5–A4, CN1, CN8*–CN9*, CN2–CN3, CN6*–CN7*; R35, R8, R41, R10, R42.
- *Principia practica, sive Postulata*: P1, P2, P3.

$\bullet$ Mercator’s book is not, properly speaking, an edition of the *Elements*, but rather a new arrangement of Euclid’s mathematics. Mercator stated the first nine axioms as principles of plane geometry, and the further five axioms as principles of the theory of proportions. He tried to separate theorems and problems, and claimed that all theorems can be proven relying on the axioms alone, while the problems require the three postulates (or practical principles). Mercator’s definition of parallel lines is that of straight lines which do not incline toward one another; the notion of inclination is left undefined. Axiom PP9 states the transitivity of parallelism, and this may be the first instance of the use of this principle to ground the Parallel Postulate. The entire theory may be considered a first step toward a direction theory of parallelism (developed in the nineteenth century). Mercator took *Elements* V, 18 (=R42) as a principle, as Euclid’s proof of it requires the assumption of the existence of the fourth proportional; he preferred to directly assume the Euclidean proposition rather than accepting axiom R1 or R17 (employed by Clavius and others to prove the proposition).

- Vitale Giordano da Bitonto, *Euclide Restituto, ovvero gli antichi elementi geometrici ristaurati e facilitati*, Roma, Angelo Bernabò, 1680.

- Book I
- *Postulati*: P1, P2, P3, A7.
- *Assiomi*: CN1–A13, CN2, CN3, CN6*, CN7*, CN8*, CN9*, CN4, CN5, P4, [P5], [P6*], [I5].

- Book VII
- *Postulati*: N12, N5.
- *Assiomi*: M3, M4, N30, N31, N32, N33, N34, N35, N36, N2, N3, N1.

- Book X
- *Postulato*: AA1.
- *Assiomi*: M11, M9, M10.

$\bullet$ Giordano’s system of principles is strongly dependent on Clavius. Giordano attempted to prove the geometrical axioms P5, P6* and I5 in the course of Book I, and his defective proof of the Parallel Postulate (pp. 45–66) is especially worthy of notice. A second edition in 1686 did not change the system of principles.

- Jacques Rohault, *Les six premiers Livres des Elemens d’Euclide*, in *Ouvres posthumes*, ed. C. Clerselier, Paris, Desprez, 1682.

- Book I
- *Demandes*: P1, P2, P3, A7.
- *Axiomes*: CN1, CN2, CN3, CN6*, CN7*, CN8*, CN9*, CN4, A4, CN5, P4, P6*, I5, I6, M1, M2, M12, M13, M14.

- Book V
- *Axiomes*: R43, R44, R45.

$\bullet$ One of the most influential treatises on Cartesianism, it also included a shortened version of the *Elements*. In Book I, Rohault mostly followed Clavius’s axiomatization. He did not include P5 among the axioms, nor did he give a proof of it, but rather stated it as an evident truth before proving *Elements* I, 29 (vol. 1, p. 60). Rohault added three axioms in Book V which are a simple variation of the Euclidean definitions of the equality and inequality of ratios, Rohault having himself defined these in a different way.

- Antoine Arnauld, *Nouveaux Élémens de géométrie*, Paris, Desprez, $1683^{2}$.

- [Book I]
- *Axiomes de l’égalité et inégalité*: CN5, A4, CN1, CN2, CN3, CN7*, CN6*, M3, M4.

- [Book II]
- *Raisons égales naturellement connues*: R46, R20, R13, R29, R30.
- *Axiomes*: R18, R47, R48, R28, R49, R10, R4, R50, R51, R52.

- [Book V]
- *Axiomes*: E11, P1, P2, I5, I17, PP6; P3, T13, E8, E12, S16–E17, E13, S17; E14.

- [Book XIII]
- *Axiomes*: E15, E16, N39.

$\bullet$ The second edition of Arnauld’s 1667 work. Several changes were made in this second edition in Books II–IV on the theory of magnitudes and proportions, mostly following Nonancourt’s *Euclides logisticus*, which had been published in 1652 and which Arnauld became familiar with in 1679–1680. The new system of principles in Book II is a modification of the 1667 edition with Nonancourt’s own axioms. Several further editions were printed, with no changes in the axiomatics. A modern critical edition, which underlines the differences between the 1667 and 1683 editions, is to be found in Descotes (2009).

- Bernard Lamy, *Les elemens de geometrie*, Paris, Pralard, 1685.

- *Principes generaux, ou Axiomes:* CN5, A4, CN1, CN2, CN3, CN7*, CN6*, CN4, CN8*, CN9*, N43.

- [Book I]
- *Propositions, ou Demandes*: I29, I3, E18, P1, P2, I31, I32, I30, I17; P3, E24, E6, E13, E8; E14, I25, I26; PP1.

- [Book II]
- *Demandes*: E19, E20.

- [Book III]
- *Demandes*: N44, N45, R53.

- [Book IV]
- *Axiomes*: I4, I33, I16, I34, I15, E21, I27, I28; E22, E23, AA2, AA3.

$\bullet$ Lamy’s volume is a textbook mostly based on Arnauld (1683). It added a few geometrical axioms, and in particular several principles on the straight line that are almost identical to one another (P1, I31, I32, I30, I17) in an attempt to formalize the incidence properties of this line. It includes no axiomatization for the theory of proportions (except for a few hints in one appendix, and the three postulates on the commensurability of ratios in Book III). Book IV deals with solid geometry. Axiom E14 is in fact expressed by Lamy as four distinct propositions. Axioms AA2 and AA3 are related to the method of indivisibles.

- Jean Prestet, *Nouveaux elemens des mathematiques, ou principes generaux de toutes les sciences qui ont les grandeurs pour objet*, Paris, Pralard, 1689$^{2}$.

- *Axiomes, ou notions communes, et principes generaux du corps entier des Mathematiques*: CN5, A4, M33, CN2, CN3, CN6*, CN7*, M12, A13, A27, A28.
- *Axiomes, ou notions communes, et principes generaux du corps entier de la Geometrie*: P1–P2–P6*, CN4, E25.

$\bullet$ The widely popular second edition of Prestet’s *Elements* (1675) appeared in two largely augmented and revised volumes in 1689. It changed the system of axioms for the theory of magnitudes, and added a few principles for geometry, which is treated of cursorily in the book.

- [John Keill], *Euclidis elementorum libri priores sex, item undecimus et duodecimus ex versione latina Federici Commandini in usum juventutis academicae*, Oxford, Theatrum Sheldonianum, 1701.

- Book I
- *Postulata*: P1, P2, P3.
- *Axiomata*: CN1, CN2, CN3, CN6*, CN7*, CN8*, CN9*, CN4, CN5, P6*, P4, P5.

- Book V
- *Axiomata*: M3, M4.

$\bullet$ A very successful edition in Britain. The first Latin edition appeared anonymously, but a number of the many subsequent Latin and English editions stated that the editor was John Keill. The Latin text mostly follows Commandino, but the system of principles in Book I is the one by Grynaeus. In the Latin editions, the axioms in Book V are those by Commadino, but in the English translation they were later suppressed.

- David Gregory, , *Euclidis quae supersunt omnia*, Oxford, Theatrum Sheldonianum, 1703.

- Book I
, *Postulata*: P1, P2, P3.
, *Communes notiones, sive Axiomata*: CN1, CN2, CN3, CN6*, CN7*, CN8*, CN9*, CN4, CN5, P4, P5, P6*.

$\bullet$ An new edition of the Greek text of the *Elements* and the other works of Euclid, together with a new Latin translation. The Greek of the *Elements* is largely based on Grynaeus’s edition, but it is improved in several respects. The system of principles, however, is left unmodified.

- Edmund Scarburgh, *The English Euclide, being The First Six Elements of Geometry, translated out of the Greek*, Oxford, Theatrum Sheldonianum, 1705.

- Book I
- *Postulates*: P1, P2–[T18], P3.
- *Axioms*: CN1, CN2, CN3, CN6*, CN7*, CN8*, CN9*, CN4, CN5, P4, [P5], P6*, PP1.

$\bullet$ A further Oxonian edition of Euclid from the beginning of the eighteenth century, this one is provided with an extensive commentary by Scarborough. He claimed that Postulate T18 was not stated by Euclid, since it is somehow included in P2, but that it should, nonetheless, be explicitly given. He thought that the Parallel Postulate is too complicated to be assumed as a principle, and preferred to add the alternative axiom PP1 on equidistance. Scarborough also offered a thoroughgoing discussion of the theory of proportions in *Elements* V, but he did not add any further axiom to this effect.

- Nicholas de Mal $\acute{\textsc {e}}$ zieu, *Elemens de geometrie de Monseigneur le Duc de Bourgogne*, Paris, Boudot 1705.

- [Book I]
- *Axiomes, ou verités connues d’elles mêmes*: CN5, A6, A4, CN1, CN2, CN3, N46, N47, N30, N48, N49.

$\bullet$ Malézieu was the mathematics teacher of the Duke of Burgundy (who, in 1705, was a child of only fourteen but who appears nonetheless as the author of this book). The volume is not an edition of Euclid, but rather a textbook in elementary mathematics which enjoyed a certain diffusion in the eighteenth century, largely inspired by the “revisionist” French attitude toward Euclid which had previously informed Richard’s and Arnauld’s work, and the latter’s dismissive opinion about a strictly formal treatment of mathematics. In particular, Malézieu’s work has no geometrical postulates at all, and simply assumes the possibility of constructions as obvious in the proofs of the propositions (see below Legendre, 1794, for a similar treatment). It only has a few common notions that are mainly employed in the arithmetical book which opens the volume, and which clearly have to be interpreted in a much more general way than they are actually stated (i.e., N46 and N47 state the commutative and distributive properties of multiplication in general). Many subsequent French books on elementary geometry in the eighteenth century simply removed any axiomatic system for the demonstrations, assuming in the body of the proofs any principle that was needed; among them, we may mention the celebrated books by Joseph Sauveur, *Geometrie élémentaire et pratique*, ed. Le Blonde, Paris, Rollin 1753 (posthumous, but widely circulating in manuscript form already for decades by this date) and by Alexis Claude Clairaut, *Elemens de geometrie*, Paris, Lambert & Durand 1741. See also below, Deidier (1739), which also has only a few common notions.

- Isaac Newton, [*The Science of Axiomatc Deduction*], around 1693–1706 (unpublished).

- *Geometriae libri duo*
- *Postulata*: P1, T22, T23.

- [*Arithmetic and Geometry*]
- *Axiomata*: CN1, CN2, CN3, R4, A4, R54.
- *Postulata*: N50, N51, ... .

- *De constructione problematum geometricorum*
- *Postulata*: T19, T20, T21.

$\bullet$ A few drafts dealing with the principles of geometry have been found among Newton’s unpublished papers and can now be read in volumes 7 (p. 388) and 8 (pp. 197, 200) of Whiteside et al. 1967–1981. The draft of some *Geometriae libri duo* has been tentatively dated by the editor to the early 1690s, possibly 1693. In it, Newton added to the first Euclidean postulate two more principles which had a broader scope than those in the *Elements* (Newton explicitly claimed that T22 implies P2 as a particular case, while T23 implies P3). Principle T22, in fact, is a modification of *Elements* I, 2-3 on the transportation of segments (cf. T15, T26) and adds nothing to the deductive power of the *Elements* (Newton thought, as did many others, that such propositions are so elementary as to be better assumed as principles). Principle T23, on the other hand, far exceeds the standard Euclidean constructions, making it possible to draw algebraic curves of higher degree and therefore to solve “all plane and solid problems” (i.e. those which require conic sections), and others as well (since reiterating constructions by T23 one can draw curves of degree greater than two). In a later passage, Newton admitted that other ways of solving solid problems may also be postulated at the beginning of geometry, such as the classical  construction, but these other methods do not allow the drawing of conic sections and other algebraic curves, and therefore T23 should be preferred. Even the basic assumption of cutting a cone with a plane (“*datum conum dato plano secare*”, p. 382) is more complicated than his own postulate. The mathematician François Viète had previously postulated the  construction in his *Supplementum geometriae* (1593); I have not included it among the present list of works, since it is neither an edition of Euclid nor does it state any further mathematical principle. Two further Newtonian drafts on axiomatics may belong to the years 1705–1706. A first one on arithmetic and geometry begins with a list of Common Notions, followed by two arithmetical postulates, and then breaks off. A second one on the construction of geometrical problems seems to envisage a geometrical axiomatics based on mechanical principles, as Newton himself had famously claimed in the Preface of the *Principia*.

- Angelo Marchetti, *Euclides reformatus*, Livorno, Celsi, 1709.

- Book I
- *Petitiones, seu Postulata*: P1, P2, T24, T25, P3, A7.
- *Axiomata, seu Pronunciata*: CN1, A13, CN8*–CN9*, CN2, CN3, M6, M7, CN5, A4, M11, T4–T8, P4, I5, E6, E7, E8–E26, E9–E27, PP5, E28, E29, CN4, E30; PP1, AA1.

- [Book III]
- *Axiomata*: R35, R8, R55, R10, R16, R37, R11, R12, R14, R56, R15, R57, R58, R59, R17.

- [Book VI]
- *Postulata*: I12, I4.
- *Axiomata*: I35, I14, I15, I8, I16.

$\bullet$ Marchetti mainly followed Borelli’s axiomatization in Book I, but with several twists. He justified the assumption of *Elements* I, 20 (=E30) among the axioms, stating that it follows from the definition of a straight line as the shortest line between two points, which he considered the only viable definition of a straight line. Axiom PP5 is equivalent to the Parallel Postulate in Borelli’s axiomatization, but Marchetti also added PP1 (p. 25) to the same effect. Axiom AA1 is added in p. 42. Marchetti’s axiomatization of the theory of proportions is to be found in his Book III. It amounts to a profound revision of the Euclidean theory, but it is not entirely new, as it was prepared by several other works of the Galileian School (Torricelli, Borelli, Viviani, and others). Marchetti himself had already published his axiomatization in his booklet *La natura della proporzione e della proporzionalità* in 1695. Axiom R55 is a kind of restatement of *Elements* V, def. 5, for commensurable magnitudes (cf. also *Elements* V, 3). Marchetti’s Book VI is on solid geometry. On Marchetti’s theory of proportions see Giusti (1993).

- Geminiano Rondelli, *Sex priora Euclidis elementa, quibus accesserunt undecimum, & duodecimum*, Bologna, Longhi, 1719.

- Book I
- *Petitiones, seu Postulata*: P1, P2, P3, S6, T7, A7.
- *Axiomata*: CN1–A13–A14, CN2, CN3, CN6*–M15, CN7*–M16, CN8*–M17, CN9*–M5, CN4, CN5, I5, I6, P4, [P5], P6*, M2, M1, M13, M12, A4, M14, A16, A24, PP2, PP10, I36.

- Book V
- *Axioma unicum*: R25.

$\bullet$ A first edition of this book had been published by Rondelli in 1684, using Clavius’s system of principles. The 1719 edition, however, added several axioms. Some of them were introduced by Rondelli, while others were taken from Ricci (1651). Rondelli held the Bologna University chair of mathematics which had been held by Ricci some years before. Rondelli proved the Parallel Postulate (P5), grounding his demonstration in the equivalent principle PP2. He also added another principle equivalent to P5, namely PP10, which, however, he did not employ in his proof of P5.

- Henry Hill, *The Six First, Together with the Eleventh and the Twelfth Books of Euclid’s Elements, Demonstrated after a New, Plain, and Easie Method*, London, Pearson, 1726.

- Book I
- *Postulates*: P1, P2, P3.
- *Axioms*: CN1, A13, A14, A29, A30, A31, CN8*, CN9*, N52, N53, CN2, CN3, N48, N49, M2, M13, CN5, A4, CN4, P6*, P4, P5.

$\bullet$ A quite typical modern edition of Euclid, that added a few algebraic principles and performed several proofs by symbolic reasoning. The entire *Elements* V on the theory of proportions is reduced to a few calculations.

- Guido Grandi, *Elementi geometrici piani e solidi di Euclide posti brevemente in volgare*, Firenze, Tartini, 1731.

- Book I
- *Dimande*: P1, P2, P3.
- *Assiomi*: CN1, CN2–CN3, CN6*–CN7*, CN8*–CN9*, CN4, CN5, P4, [P5], P6*, I5.

$\bullet$ Grandi’s edition of the *Elements* is very classical, and draws on Grynaeus’s system of principles, adding to it a rather standard axiom by Clavius (I5). At the time of Grandi’s edition of Euclid, Gerolamo Saccheri had already written his *Euclides vindicatus* (published in 1733), in which he disproved Clavius’s proof of the Parallel Postulate (P5). Even though Grandi was in personal relations with Saccheri, however, he proved P5 following Clavius’s demonstration.

- Manoel de Campos, *Elementos de geometria plana, e solida, segundo a ordem de Euclides*, Lisboa, Rita Cassiana, 1735.

- Book I
- *Postulados*: P1, P2, P3.
- *Axiomas*: CN1, CN2, CN3, CN6*, CN7*, CN8*, CN9*, CN4, CN5, P4, P5 [PP3], PP4, P6*, I5.

$\bullet$ A Potuguese edition of the *Elements*, employing a rather common choice of principles (see Grandi above). Campos stated that Tacquet’s axiom PP3 might be substituted for the Parallel Postulate (P5); in any case, he also added axiom PP4, which is itself equivalent to P5.

- Antoine Deidier, *La science des géomètres*, Paris, Jombert, 1739.

- Book I
- *Axiomes*: CN5, A4, CN2, CN3, N48–N49, CN6*–CN7*–N54–N55, CN8*–CN9*, AA5, CN1.

$\bullet$ A quite famous French edition in the eighteenth century, which does not follow Euclid’s text but rather systematizes it, Deidier’s version of the *Elements* has no geometrical principles, and grounds its demonstration on the definitions of geometrical figures. See above Malézieu (1705) for a similar example.

- Samuel Klingenstierna, *Euclidis elementorum libri sex priores una cum undecimo et duodecimo*, Upsala, Höjer, 1741.

- Book I
- *Postulata*: P1, P2, P3.
- *Axiomata*: CN1, CN2, CN3, CN6*, CN7*, CN8*, CN9*, CN4, CN5, P6*, P4, P5.

- Book V
- *Axiomata*: M3, M4.

$\bullet$ An Euclidean edition by one of the greatest Swedish mathematicians. The system of principles follows Grynaeus in Book I, but the additional axioms in Book V bear trace of Keill’s edition from 1701. A Swedish translation of Euclid was made a few years later by Strömer (see below).

- Mårten Str $\ddot{\textsc {o}}$ mer, *Euclidis Elementa eller Grundeliga inledning til geometrien, til riksens ungdoms tienst på swenska språket utgifwen*, Upsala, Kiesewetter, 1744.

- Book I
- *Postulater*: P1, P2, P3.
- *Axiomer*: CN1, CN2, CN3, CN6*, CN7*, CN8*, CN9*, CN4, CN5, P6*, P4, P5.

$\bullet$ Grynaeus’s system of principles. Possibly the most widely read (and republished) edition of the *Elements* in Swedish.

- Thomas Simpson, *Elements of Plane Geometry*, London, Farrer & Turner, 1747.

- Book I
- *Postulates, or Petitions*: P1, P2, P3, I37–T26.
- *Axioms, or Self-evident Truths*: CN1, CN5, A4, CN2, CN3, CN6*–CN7*, P4, PP10, E31.

- [Book IV]
- *Axioms*: R60, R61, R62, R8, R10.
- [*Treatise of Regular Solids*]
- *Postulatum*: E32.

$\bullet$ A textbook rearranging the geometrical results of the *Elements*. Simpson’s volume substituted for the Parallel Postulate (P5) a principle based on equidistance (PP10), and assumed a few basic or controversial theorems of the *Elements* (Book One, propositions 2, 3, 4, 11, 12, 23) as axioms. Axiom I37–T26 allows the transportation of segments and angles (cf. Hilbert’s Axioms III, 1, 4, and 6, for a similar axiomatic treatment), but Simpson incidentally added that it should also ground the possibility to construct parallel lines (in his definition: equidistant lines) by parallel displacement of a segment, therefore assuming a second time the Parallel Postulate. Simpson’s Book IV is a treatment of the Euclidean theory of proportions in *Elements* V. Simpson added in the same volume a treatise on solid elementary geometry (*Elements* XI and XII), with its own new postulate. A second, revised edition of Simpson’s *Elements* appeared in 1760 (see below).

- Robert Simson, *Euclidis Elementorum libri priores sex item undecimus et duodecimus ex versione latina Federici Commandini*, Glasgow, Foulis, 1756.
- English version as *The Elements of Euclid, viz. the first Six Books together with the Eleventh and Twelfth. In this Edition, the Errors, by which Theon, or others, have long ago Vitiated these Books, are Corrected, and some of Euclid’s Demonstrations are Restored*, Glasgow, Foulis, 1756.

- Book I
- *Postulata* (*Postulates*): P1, P2, P3.
- *Axiomata* (*Axioms*): CN1, CN2, CN3, CN6*, CN7*, CN8*, CN9*, CN4, CN5, P6*, P4, [P5].

- Book V
- *Axiomata* (*Axioms*): M3, M4, M34, M35.

$\bullet$ Grynaeus’s system of principles in Book I. An important edition of the geometrical books of Euclid, Simson’s volume also attempted to restore the original text of the *Elements* after the many corruptions and modifications added in the previous early modern editions. To this effect, Simson’s commentary is put at the end of the book, so that it would not interfere with the original text, and the system of principles is the most conservative possible. In *Elements* I, Simson placed the Parallel Postulate posterior to the other axioms, since he believed that it should, in fact, be ranged among the theorems, and gave the old proof of it by Proclus (even though it had been refuted several times in the preceding literature). In *Elements* V, he added four axioms taken from, or inspired by, Campano to deal with the theory of proportions. The book enjoyed several reprints and translations into other languages (including German and Portuguese), and was used by several other British authors as the foundational text for their editions; see for instance the editions by John Playfair (1795, below), and Alexander Ingram (Edinburgh, Pillans 1799). The French edition by Castillon (Berlin, Société typographique 1767) also followed Simson’s *Elements*, and made use of the same system of axioms (with an even more naïve proof of the Parallel Postulate).

- Samuel K$\ddot{\textsc {o}}$ nig, *Elemens de geometrie contenant les six premiers livres d’Euclide*, Den Haag, Scheurleer, 1758.

- Book I
- *Demandes*: P1, P2, P3.
- *Axiomes ou notions communes*: CN1, CN2, CN3, CN6*, CN7*, CN8*, CN9*, CN5, T27, P4, [P5–PP2], P6*.

- Book II
- *Axiomes*: A4, E16.

- Book III
- *Axiomes*: E8, E33.

- Book V
- *Demandes*: M36, A32, [N37].

$\bullet$ König’s edition of the *Elements* attempted to offer a quasi-formal account of the Euclidean proofs. König employed Grynaeus’s system of principles in Book I, but added several axioms in the subsequent books. The only modification in the axiomatization of *Elements* I is the substitution, for CN4, of T27. The latter principle is itself quite classical, as Euclid himself had employed the terms “similar and equal” () to mean congruent figures in *Elements* XI, def. 10. In the Early Modern Age this formula was often used to explicitly define congruence, but König may be the first to have employed it as an axiom. The Parallel Postulate (P5) is included among the axioms, but the further discussion shows that König wanted to replace it with axiom PP2, which he found simpler and from which he proved P5. Book V does not have axioms, but rather two postulates; it also has an Appendix on logarithms which departs from the Euclidean text, and in which König added a *demande* stating that it is possible to make multiplications and divisions of any magnitude (something similar to axiom N37). In a second edition printed in 1762, the mathematician J.J. Blassière added a version of Books XI and XII in the style of König—without, however, adding any further principle.

- Abraham Gotthelf K$\ddot{\textsc {a}}$ stner, *Anfangsgründe der Arithmetik, Geometrie, ebenen und sphärischen Trigonometrie und Perspectiv*, Göttingen, Vandenhoeck, 1758.

- [Book I]
- *Forderungen*: P1–P2–S20, P3.
- *Grundsätze*: I17–P6*, I5–I6, CN4, T10–T12, P4, E6, C20, I38; P5; I33.

$\bullet$ Not an edition of Euclid’s *Elements*, but the most important German textbook on elementary mathematics in the eighteenth century. Kästner did not axiomatize arithmetic, but endorsed a highly composite system of axioms for geometry, which he took from several previous Euclidean editions (Kästner was a remarkable historian of mathematics). He also added a few practical “postulates”. The *Anfangsgründe* went through many editions in Germany and deeply influenced research on the foundations of geometry.

- Thomas Simpson, *Elements of Geometry*, London, Nourse, 1760.

- Book I
- *Axioms, or Self-evident Truths*: CN1, CN5, A4, CN2, CN3, CN6*–CN7*, P4, I17, PP11, E31.
- *Postulates, or Petitions*: P1, P2, P3, [I37–T26].

- [Book IV]
- *Axioms*: R63, R61, R60, R62, R8, R41, R10, R64, M37.

- [Book VII]
- *Axiom*: E34.
- *Postulate*: I39.

- [Book VIII]
- *Postulates*: AA1, AA6, E35.

$\bullet$ A deeply revised edition of Simpson’s work from 1747, this volume rearranges in eight books the geometrical results of the *Elements*. With respect to the first edition, Simpson slightly modified his version of the Parallel Postulate (now PP11), and while he still assumed rigid motion through postulate I37–T26, he deleted any reference to parallel lines in it, and even claimed that it can be proven with a standard Euclidean procedure. He also introduced the classical axiom I17. Simpson’s Book IV extended the axiomatic basis of the theory of proportions; in particular, his axiom M37 is the distributive property of addition and multiplication. Simpson’s Book VII is a reworking (with different principles) of his treatise on solid geometry, while his newly written Book VIII deals with the method of exhaustion and proves some results of *Elements* X. In it, axiom AA6 is a formulation of Archimedes’s Axiom, while axiom E35 is a modification of Lamy’s E19 and E20 (which refer to the areas of a circle and a polygon, while Simpson’s E35 refers to circumferences and perimeters). The volume also contains a few sections further developing elementary geometry with new methods, as well as many historical and technical notes on Euclid’s theorems.

- Johann Friedrich Lorenz, *Euklid’s Geometrie, oder die sechs ersten Bücher der Elemente nebst dem eilften und zwölften aus dem Griechichen übersetz*, Halle, Weisenhaus, 1773.

- Book I
- *Forderungen*: P1, P2, P3.
- *Grundsätze*: CN1, CN2, CN3, CN6*, CN7*, CN8*, CN9*, CN4, CN5, P4, P5, P6*; [PP12].

$\bullet$ Grynaeus’s system of principles. Translated into German directly from Grynaeus’s Greek text, Lorenz’s edition enjoyed a wide popularity in Germany by the end of the eighteenth and the beginning of the nineteenth century, and it was consistently referred to by several studies on the theory of parallels. In the preface to Lorenz’s edition, the mathematician Andreas Segner discussed the provability of P5, proposing a demonstration of it which implicitly relied on the new principle PP12. Lorenz, in his later *Grundriß der reinen und angewandten Mathematik, oder der erste Cursus der gesamten Mathematik* (Helmstädt, Fleckeisen, 1791), reworked the entire system of axioms, and explicitly included PP12 among the *Grundlehren*; the latter book, however, was not an edition of Euclid. Lorenz’s 1773 *Geometrie* went through many reprints and inspired the following German editions of Euclid by Michelsen (Berlin, Maßdorff 1791), Hauff (Marburg, Akademie 1797) and Hoffmann (Mainz, Kupferberg 1829), among others, which endorsed the same system of principles.

- John Bonnycastle, *Elements of Geometry*, London, Johnson, 1789.

- Book I
- *Postulates*: P1, P2, P3, PP2.
- *Axioms*: CN1, CN2, CN3, CN6*, CN7*, CN8*, CN9*, A4, CN4.

$\bullet$ A common English edition of the geometrical books of the *Elements* which alters from time to time the Euclidean deductive structure. Principle PP2 is a substitute for P5 and is, surprisingly, stated as a postulate rather than an axiom.

- Adrien-Marie Legendre, *Éléments de géométrie avec des notes*, Paris, Didot, 1794.

- Book I
- *Axiomes*: CN1, CN2, CN3, N30, N31, CN5, A4, I31, CN4, [P5]. (...)

$\bullet$ One of the most famous Euclidean editions ever, Legendre’s *Elements* does not follow the Greek text but develops the same results in a different order and with different proofs. Legendre did not include any postulate in his list of principles, and stated that the axioms which he gave at the beginning of the first book are merely illustrative of an infinity of other similar axioms. At later times, people complained that he had erased the distinction between axioms and postulates [see for instance the opinion of Grassmann and Houël in Voelke (2005), p. 63], even though this does not seem to have been Legendre’s aim. The book went through many editions, with several subsequent modifications, and in most of them Legendre attempted (in various ways) to prove the Parallel Postulate (P5). The system of axioms also changed since the second edition (see below). Legendre’s book was later translated into several languages.

- John Playfair, *Elements of Geometry, containing the first six books of Euclid with two books on the geometry of solids*, Edinburgh, Bell & Bradfute, 1795.

- Book I
- *Postulates*: P1, P2, P3.
- *Axioms*: CN1, CN2, CN3, CN6*, CN7*, CN8*, CN9*, CN4, CN5, P4, PP13.

- Book V
- *Axioms*: M3, M4, M34, M35.

$\bullet$ The edition of the *Elements* by Playfair is mainly based on Simson (1756), who had tried to restore a text closer to the Greek original. Playfair, however, allowed a few modifications (especially in the proofs of Book V) for the sake of clarity and rigor. Among these, the introduction of axiom PP13 (“Playfair’s Axiom”) as a substitute for the Parallel Postulate (P5) was especially successful. The latter principle had already been recognized as equivalent to P5 by Ibn al-Haytham and others, even though it had never been used explicitly to this effect in an edition of the *Elements*. On Playfair’s edition, see Ackerberg-Hastings (2002).

- Adrien-Marie Legendre, *Éléments de géométrie avec des notes*, Paris, Didot, 1799$^{2}$.

- Book I
- *Axiomes*: CN1, CN5, A4, I31, CN4, [P5]. (...)

$\bullet$ The second edition of Legendre’s celebrated *Éléments de géométrie* offered a significant simplification of the system of axioms employed in the first one from 1794. Further editions of this work followed the present list.

- Vincenzo Flauti, *I primi sei libri degli elementi di Euclide*, Napoli, Stamperia Reale, 1810.

- Book I
- *Postulati*: P1, P2, P3, P4, [P5].
- *Assiomi, o nozioni comuni*: CN1, CN2, CN3, CN6*, CN7*, CN8*, CN9*, CN4, CN5, P6*.

- Book V
- *Postulato*: R1.

$\bullet$ An edition of the *Elements* which enjoyed broad diffusion in Italy, and which went through many reprints as one volume of Flauti’s *Corso di geometria elementare e sublime*. It is very rich in historical and mathematical notes, and in some editions, Flauti claimed to have proven the Parallel Postulate (P5) using an old argument by Nasīr ad-Dīn (in further editions, however, he acknowledged his mistake here).

- François Peyrard, , *Euclidis quae supersunt*, *Les oeuvres d’Euclide*, Paris, Patris, 1814.

- Book I
, *Postulata*, *Demandes*: P1, P2, P3, P4, P5, P6*.
, *Notiones communes*, *Notions communes*: CN1, CN2, CN3, CN6*, CN7*, CN8*, CN9*, CN4, CN5.

$\bullet$ The crucially important edition of Euclid’s works by Peyrard gives the original Greek text and a translation into Latin and French. It was the first edition to contain a thoroughgoing discussion of several extant Greek manuscripts, and, in particular, it mainly followed the text of the Vatican manuscript **P** (see above), found by Peyrard himself, which appears to represent the oldest version of the *Elements* which survived into the modern age. Peyrard emended several mistakes in the previous Greek editions by Grynaeus (1533) and Gregory (1703), as well as in Commandino’s translation (still considered as counting among the best editions of Euclid); yet, in several places he preferred Grynaeus’s version to the text found in his newly discovered Greek manuscript. It was preceded by an incomplete edition of the *Elements* in 1804 by Peyrard which did not make use of the Vatican manuscript. The 1814 edition was prefaced by an appraisal of it by Lagrange, Legendre and Dalambre.

In the following years, several older editions were reprinted many times, but Peyrard’s text and system of principles gained a growing acceptance by the scholarly community. Another Latin edition based on Peyrard’s text was prepared by Niede in 1825, and in 1826 it was translated into English by Philips. In 1824, Camerer and Hauber published, in Germany, a very influential and heavily annotated edition of the *Elements* also based on Peyrard’s manuscript (and axioms). In 1826–1829, E.F. August published another edition of the Greek text, also following Peyrard’s manuscript and correcting the latter’s edition in a few places. Many years later (1883–1916), Heiberg and Menge built their edition of Euclid on the same Vatican manuscript found by Peyrard (even though they emended and integrated it in several places), and this edition is still the most authoritative reconstruction of the text of the *Elements* that we have.
